# Phage Genes Induce Quorum Sensing Signal Release through Membrane Vesicle Formation

**DOI:** 10.1264/jsme2.ME21067

**Published:** 2022-01-27

**Authors:** Marina Yasuda, Tatsuya Yamamoto, Toshiki Nagakubo, Kana Morinaga, Nozomu Obana, Nobuhiko Nomura, Masanori Toyofuku

**Affiliations:** 1 Graduate School of Life and Environmental Sciences, University of Tsukuba, Japan; 2 Faculty of Life and Environmental Sciences, University of Tsukuba, Japan; 3 Transborder Medical Research Center, Faculty of Medicine, University of Tsukuba, Japan; 4 Microbiology Research Center for Sustainability, University of Tsukuba, Japan; 5 Suntory Rising Stars Encouragement Program in Life Sciences (SunRiSE), Japan

**Keywords:** quorum sensing, phage, membrane vesicle, *N*-acyl-homoserine lactone

## Abstract

Membrane vesicles (MVs) released from the bacterium *Paracoccus denitrificans* Pd1222 are enriched with the quorum sensing (QS) signaling molecule *N*-hexadecanoyl-**l**-homoserine lactone (C16-HSL). However, the biogenesis of MVs in Pd1222 remains unclear. Investigations on MV formation are crucial for obtaining a more detailed understanding of the dynamics of MV-assisted signaling. In the present study, live-cell imaging showed that *P. denitrificans* Pd1222 produced MVs through cell lysis under DNA-damaging conditions. DNA sequencing of MVs and a transcriptome ana­lysis of cells indicated that the expression of a prophage region was up-regulated at the onset of MV formation under DNA-damaging conditions. A further sequence ana­lysis identified a putative endolysin (Pden_0381) and holin (Pden_0382) in the prophage region. The expression of these genes was regulated by RecA. Using gene knockout mutants, we showed that prophage-encoded endolysin was critical for MV formation by *P. denitrificans* Pd1222 under DNA-damaging conditions. MV triggering by endolysin was dependent on the putative holin, which presumably transported endolysin to the periplasmic space. C16-HSL quantification revealed that more signals were released into the milieu as a consequence of the effects of endolysin. Using a QS reporter strain, we found that the QS response in *P. denitrificans* was stimulated by inducing the expression of endolysin. Collectively, these results provide novel insights into the mechanisms by which a bacterial cell-to-cell communication system is manipulated by phage genes.

Quorum sensing (QS) is a cell-to-cell communication system that is utilized by various bacteria (Whiteley *et al.*, 2017). In this system, signaling molecules are released from cells and recognized by neighboring cells, which allows bacteria to dose-dependently regulate gene expression in the entire bacterial population. An essential process in QS is the transfer of signaling molecules between cells. The conventional QS model assumes that signaling molecules diffuse freely among cells. Consistent with this model, hydrophilic signaling molecules, such as *N*-butyryl-**l**-homoserine lactone, have been reported to diffuse freely into and out of cells (Pearson *et al.*, 1999). In contrast, a number of hydrophobic signaling molecules have been shown to accumulate in the cell envelope (Pearson *et al.*, 1999; Chan *et al.*, 2007; Buroni *et al.*, 2009). Therefore, the mode of diffusion of signaling molecules remains unclear.

Recent studies revealed that MVs contain hydrophobic signaling molecules and function as carriers in several bacteria (Mashburn and Whiteley, 2005; Schertzer and Whiteley, 2012; Brameyer *et al.*, 2018). *Paracoccus denitrificans* is a non-motile Gram-negative bacterium that produces and utilizes the *N*-acyl-homoserine lactone (AHL) QS signal, *N*-hexadecanoyl-**l**-homoserine lactone (C16-HSL). We previously demonstrated that this bacterium enriched C16-HSL in membrane vesicles (MVs) and transmitted them to other cells, which regulated cell aggregation (Toyofuku *et al.*, 2017b). Although C16-HSL alone does not diffuse well in aqueous environments due to its high hydrophobicity, MVs allow C16-HSL to disperse. This indicates that *P. denitrificans* employs a MV-based bacterial communication system. In systems in which signals are carried by MVs, MV formation is an important step in understanding how QS signals are released. However, the mechanisms underlying MV formation in *P. denitrificans* remain unclear.

MVs are formed in the cellular membranes of various bacteria. The canonical model for MV formation in Gram-negative bacteria is blebbing of the outer membrane (Jan, 2017). DNA damage was recently found to induce MV formation *via* explosive cell lysis in *P. aeruginosa* and through bubbling cell death in *Bacillus subtilis* and *Corynebacterium glutamicum* (Turnbull *et al.*, 2016; Toyofuku *et al.*, 2017a; Nagakubo *et al.*, 2021). Prophage genes, namely, *lys* and *hol*, play important roles in these processes. The *hol*-*lys* system is known to release phage particles from host bacteria through cell lysis during the phage lytic cycle upon the sensing of DNA-damaging stress by host bacteria. *lys* and *hol* encode endolysin and holin, respectively, and endolysin is transferred from the cytoplasm to the periplasm through pores created by holin in the inner membrane. Endolysin degrades the bacterial cell wall by enzymatic activity, which causes cell lysis and the release of phage particles. A previous study demonstrated that the DNA-damaging agent, mitomycin C (MMC) induced MV production in *P. denitrificans* Pd1222 (Toyofuku *et al.*, 2017b); however, the involvement of prophage genes has not yet been investigated.

In the present study, we showed that MV formation in *P. denitrificans* was stimulated by prophage gene expression, resulting in the release of C16-HSL. Our results revealed that cell-to-cell communication in *P. denitrificans* may be stimulated by a phage-derived gene *via* MV production, which indicates a new role for phage genes in bacterial communication.

## Materials and Methods

### Bacterial strains and growth conditions

The strains used in the present study are listed in Table S1. *P. denitrificans* was grown in tryptic soy broth (TSB) at 37°C according to standard procedures. *Escherichia coli* and *Chromobacterium violaceum* were grown in Luria broth at 37 and 30°C, respectively. The antibiotics used for selection were 50‍ ‍μg‍ ‍mL^–1^ kanamycin and 1‍ ‍μg mL^–1^ tetracycline. Cumate (Sigma-Aldrich) was added to the culture at OD_600_=0.1 to induce gene expression where necessary, and the culture was incubated for a further 8 h. MVs were isolated from *P. denitrificans* Pd1222 cultures that were inoculated at an initial OD_600_=0.01 after a 24-h incubation at 200‍ ‍rpm. To induce MV formation using MMC in *P. denitrificans* Pd1222, strains were inoculated at an initial OD_600_ of 0.1 and then incubated at 37°C and 200‍ ‍rpm. When growth reached OD_600_=0.35, MMC was added to the culture, which was incubated for a further 5 h under the same conditions.

### Construction of plasmids and targeted mutations

The primers used in the present study are listed in Table S2. *P. denitrificans* Pd1222 gene deletion mutants were generated by homologous recombination with pK18mobsacB, as described below (Shearer *et al.*, 1999). To generate a Pden_0381 mutant, the flanking regions of the target gene were amplified using the 0381F1/0381R1 and 0381F2/0381R2 primer sets. PCR products were mixed in equimolar proportions and used for overlap extension PCR with the 0381overF/0381overR primer sets. The PCR product and pK18mobsacB were treated with *Eco*RI and *Hin*dIII, assembled using NEBuilder Hifi DNA assembly (NEB), and delivered into *P. denitrificans* Pd1222 by conjugation using *E. coli* S17-1 (Simon *et al.*, 1986). Pden_0382 in WT was deleted using pK18mobsacB-ΔPden_0382_WT which was generated with the 0382wtF1/0382wtR1, 0382F2/0382R2, and 0382wtoverF/0382overR primer sets. Pden_0382 in ΔPden_0381 was deleted using pK18mobsacB-ΔPden_0382_ΔPden_0381 which was generated with the 0382 lysF1/0382 lysR1, 0382F2/0382R2, and 0382 lysoverF/0382overR primer sets. Cumate-inducible plasmids (pQF_0381, pQF_0382, and pQF_0381_0382) were constructed by cloning target genes derived from the Pd1222 genome into pQF using the Ind0381F/Ind0381R, Ind0382F/Ind0382R, and Ind0381_0382F/Ind0381_0382R primer sets, respectively. The amplified fragments and pQF were treated with *Xba*I and *Kpn*I and ligated using Mighty Mix (Takara Bio).

### MV isolation and quantification

MVs were isolated and quantified as previously described (Toyofuku *et al.*, 2017b). The cell culture was centrifuged at 6,000×*g* at 4°C for 20‍ ‍min, and the supernatant was filtered through a polyvinylidene difluoride filter with a pore size of 0.22‍ ‍μm (Merck Millipore) to remove cells. The supernatant was ultracentrifuged at 150,000×*g* at 4°C for 1 h using an MLA-50 rotor (Beckman Coulter). Regarding larger volumes, the supernatant was ultracentrifuged at 200,000×*g* at 4°C for 90‍ ‍min with a Type 45 Ti rotor (Beckman Coulter). The MV pellet was washed and suspended in 10‍ ‍mM HEPES/0.85% NaCl (pH 6.8).

Regarding density gradient centrifugation, a working solution (10–45% iodixanol/10‍ ‍mM HEPES [pH 6.8]/0.85% NaCl) was prepared using Optiprep (Alere Technology AS). The MV pellet was suspended in a 45% iodixanol solution and transferred to a centrifuge tube. An iodixanol solution (15–40%) was multilayered by concentration, followed by ultracentrifugation at 100,000×*g* at 4°C for 3 h using an MLS-50 rotor. After MV fractions were collected using pipette tips, MV samples were washed and suspended in 10‍ ‍mM HEPES/0.85% NaCl (pH 6.8). The amount of MVs was assessed using membrane and protein assays. In the membrane assay, a Varioskan Flash Reader (Thermo Scientific) was used to measure the relative fluorescent units (RFU) of FM4-64 (Ex/Em=558‍ ‍nm/734‍ ‍nm) in a 96-well black plate (Grainer Bio-one). In the protein assay, MVs were quantified using a BCA protein assay kit (Nacalai Tesque). A Varioskan Flash Reader (Thermo Scientific) was used to measure absorbance at 562‍ ‍nm in a 96-well plate (IWAKI).

### Microscopy

Regarding live-cell imaging, confocal laser microscopy was performed using an LSM780 system (Carl Zeiss) and a 100× oil objective lens (Alpha Plan-Apochromat 100×/1.46 Oil DIC M27 Elyra; Carl Zeiss) in the Airyscan mode. FM4-64 (2.5‍ ‍μg mL^–1^) was added to the cell culture at the same time as MMC (50‍ ‍ng‍ ‍mL^–1^), and the sample was incubated at 200‍ ‍rpm for 1‍ ‍h. Stained cells (2‍ ‍μL) were then placed on a thick agar pad containing TSB, 0.8% Bacto agar, 2.5‍ ‍μg mL^–1^ FM4-64, and 50‍ ‍ng mL^–1^ MMC. The sample was well dried and placed on a glass-bottomed dish, with the sample facing the glass bottom (IWAKI). Images were obtained every 30‍ ‍min, and Airyscan processing was performed using ZEN Black software (Carl Zeiss). The ImageJ plugin StackReg (Thevenaz *et al.*, 1998) was used to correct the XY-axis in the movie.

A 100× oil objective lens (EC Plan-Neofluar 100×/1.30 Oil Ph 3 M27; Carl Zeiss) was used to observe phase contrast. Cell samples (2‍ ‍μL) were placed on a 0.8% purified agar pad on a glass slide and covered with a glass coverslip. Image processing was performed using ZEN Blue software (Carl Zeiss).

### Genome data and software

*P. denitrificans* Pd1222 genome data were obtained from GenBank (GenBank assembly accession: GCA_000203895.1) and used as a reference for prophage region prediction, DNA sequencing (DNA-seq), and RNA sequencing (RNA-seq) ana­lyses. Docker (https://www.docker.com/) was used to run the bioinformatics software. Docker containers of software were obtained from the BioContainers Registry (Bai *et al.*, 2021).

### Prophage region prediction

The phage region in the *P. denitrificans* Pd1222 genome was predicted using *PhiSpy* software (version 4.2.19) (Akhter *et al.*, 2012) with the VOGDB hmm file (release number vog206, https://vogdb.org/). Prior to predictions, the downloaded hmm files were merged into a single file and formatted into a binary format for *PhiSpy* using the hmmpress command in HMMER (version 3.3.2) (Eddy, 2011).

### DNA extraction and sequencing

DNA was extracted from the isolated MV fraction using ISOPLANT (Nippon Gene) according to the manufacturer’s instructions. A Nextera XT DNA library preparation kit (Illumina) was used for paired-end libraries. DNA-seq was performed using an Illumina MiSeq^TM^ system.

Low-quality read removal and adapter trimming of the raw FASTQ file obtained were performed using fastp software (version 0.20.1) (Chen *et al.*, 2018) with the “-q 20 -3” options. The cleaned FASTQ file was mapped to the *P. denitrificans* Pd1222 genome using Bowtie2 (version 2.4.4). Prior to mapping, a FASTA file of the genome was indexed using the “Bowtie-build” command in Bowtie2. The resulting BAM files were indexed using SAMtools (version 1.12) (Li *et al.*, 2009). A GTF file of the *P. denitrificans* Pd1222 genome partitioned into 1,000-bp windows was created using R script, and the number of reads mapped to every 1,000 bp in the genome was counted using featureCounts software (version 2.0.1) (Liao *et al.*, 2014) with the GTF file and “-p -O -M -C -f -t region -g ID -s 0” options. The number of reads mapped to the gene was also counted with the GTF file of *P. denitrificans* Pd1222 and the “-p -O -M -C -f -t CDS -g gene_id -s 0” options.

### RNA extraction and sequencing

Total RNA was extracted using the SV Total RNA Isolation System (Promega) from two biological replicates of *P. denitrificans* Pd1222 WT and Δ*recA* cultured with or without MMC. Furthermore, we performed a DNase treatment to remove residual total DNA using DNase I, a recombinant, RNase-free solution (Roche). All processes were conducted according to the manufacturer’s instructions. RNA samples were sent to an outside facility (Macrogen) for library generation using the TruSeq Stranded Total RNA Library Preparation Kit (Illumina) and NEBNext rRNA Depletion Kit (Bacteria) (NEB), and were sequenced using the NovaSeq 6000 system (Illumina).

Low-quality read removal and adapter trimming of the raw FASTQ files obtained was performed using fastp software with the “-q 30 -3” options. A fasta file of the *P. denitrificans* Pd1222 genome was indexed with the GTF file of the genome using STAR software (version 2.7.5a) (Dobin *et al.*, 2013) with the “--runMode genomeGenerate --sjdbGTFfeatureExon gene --genomeSAindexNbases 10” options, and the cleaned FASTQ files were mapped to the index using STAR with the “--outSAMtype BAM SortedByCoordinate --alignIntronMax 1” options. The BAM files obtained were indexed using SAMtools, and the number of reads mapped to each gene was counted using featureCounts software with the GFT file of the *P. denitrificans* Pd1222 genome and the “-p -O -M -C -f -t CDS -g gene_id -s 2” options.

The batch effect between replicates was corrected using ComBat-seq (Zhang *et al.*, 2020) in the R package sva (version 3.38.0) (Leek *et al.*, 2012), and a differential gene expression ana­lysis was performed using the R package edgeR (version 3.32.1) with the corrected count data. Low expression levels of 14 genes were filtered out from the corrected count data using the filterByExpr function with default settings. Filtered count data were normalized using the trimmed mean of M-values method with the calcNormFactors function, and common, trended, and tagwise dispersions were estimated using the Cox-Reid profile-adjusted likelihood method with the estimateGLMCommonDisp, estimateGLMTrendedDisp, and estimateGLMTagwiseDisp functions, respectively. Differentially expressed genes were examined using the quasi-likelihood F-test with the glmQLFit and glmQLFTest functions, and *p*-values were adjusted using the Benjamini and Hochberg method with the topTags function.

### Sequence deposition

Sequence data obtained in the present study have been deposited in the DNA Data Bank of Japan (DDBJ) Sequence Read Archive (DRA) under accession numbers DRA004929 and DRA013013 for DNA-seq and RNA-seq, respectively.

### Protein identification

Proteins associated with MVs were identified as follows: Proteins were quantified with a BCA protein assay kit prior to SDS-PAGE, to adjust the amount of protein between samples. MV samples were mixed with an equivalent volume of a sample buffer containing 0.1 M Tris-HCl (pH 6.8), 20% (v/v) glycerol, 4% (w/v) SDS, 5% (v/v) 2-mercaptoethanol, and 0.2% bromophenol blue, and then incubated in a heat block (95°C, 5‍ ‍min). Five micrograms of protein was applied to each lane of an SDS-polyacrylamide gel comprising a 4.5% stacking gel and 12% separating gel. Gels were stained with Coomassie Brilliant Blue and imaged using FUSION SL (Vilber-Lourmat). Protein bands were excised from the gel and in-gel digested with trypsin, followed by the addition of a carbamidomethyl group to the -SH groups of the cysteine residues in the peptides using iodoacetamide (Nagakubo *et al.*, 2021). The resulting peptides were purified and concentrated using a Zip Tip (Merck Millipore). Peptides were then analyzed using matrix-assisted laser desorption/ionization time-of-flight mass spectrometry (MALDI-TOF MS). The mass spectra derived from these peptides were analyzed using the MASCOT database search (Matrix Science) against the proteobacteria protein database from the NCBI plot. Database searches were performed using trypsin cleavage specificity, with no possible miscleavage. The carbamidomethylation of cysteines was set as a fixed modification, and peptide tolerance was set as 100 ppm. The proteins derived from *P. denitrificans* Pd1222 that showed the highest MASCOT scores (above the significance threshold) were assigned to the bands.

### C16-HSL bioassay

In the C16-HSL bioassay, *C. violaceum* VIR24/pPROBE-P*vioA*-gfp (Toyofuku *et al.*, 2017b) and *P. denitrificans* Pd1222Δ*pdnI*Δ*pxm*/pPLlas were used as C16-HSL reporter strains. In Pd1222 Δ*pdnI*/pPLlas, gfp(ASV) expression is induced in response to exogenous C12- to C18-HSL (Toyofuku *et al.*, 2017b; Morinaga *et al.*, 2018). Pxm is involved in extracellular matrix production in *P. denitrificans* Pd1222 (K. Morinaga, unpublished). Ten microliters of the filtered supernatants of the *P. denitrificans* Pd1222 culture was added to 1‍ ‍mL of medium in each well of a 24-well flat-bottomed plate. Overnight cultures of the reporter strains were inoculated at an OD_600_ of 0.01. The supernatant and reporter strains were incubated at 400‍ ‍rpm at 30°C for 12 h. Synthetic C16-HSL (Cayman Chemical) was added at final concentrations of 0–10‍ ‍μM. After the incubation, the cell culture was centrifuged at 16,000×*g* at 4°C for 1‍ ‍min. Cells were then washed, dissolved in PBS, and placed in a 96-well black plate (Grainer Bio-one). QS promoter activity was calculated by normalizing RFU (Ex/Em=475‍ ‍nm/515‍ ‍nm) with the OD_600_ value, measured using a Varioskan Flash Reader (Thermo Scientific).

### C16-HSL quantification using liquid chromatography and mass spectrometry

Cell cultures were centrifuged at 6,000×*g* at 4°C for 20‍ ‍min, and the supernatant was filtered using a polyvinylidene fluoride filter with a pore size of 0.22‍ ‍μm to remove cells (resulting in the total supernatant). The total supernatant was ultracentrifuged at 150,000×*g* at 4°C for 1 h and further separated into MV pellets and the remaining supernatant. The pellet was dissolved and washed with 10‍ ‍mM HEPES/0.85% NaCl and then ultracentrifuged at 150,000×*g* at 4°C for 1 h. The resulting precipitate was then suspended in the same buffer (producing the MV sample). C16-HSL was extracted as previously described (Morinaga *et al.*, 2020). A three-fold volume of acidified ethyl acetate was added to each sample and mixed well. The solution was centrifuged at 6,000×*g* at 25°C for 1‍ ‍min. The organic layer was collected and dried in a draft chamber. C16-HSL samples were dissolved in dimethyl sulfoxide. C16-HSL was quantified using high-performance liquid chromatography-mass spectrometry (HPLC-MS) (Buddrus-Schiemann *et al.*, 2014). This ana­lysis was performed using the Nexera X2 system and LCMS-8050 (Shimadzu) equipped with a Kinetex EVO C8 column (PHENOMENEX). The conditions used were as follows: flow rate, 0.2‍ ‍mL‍ ‍min^–1^; temperature, 40°C; solvent A, 1% acetate; solvent B, acetonitrile. The sample was injected into the column, and C16-HSL was eluted from the column using the following gradient: 0–3‍ ‍min, 60–98% solvent B; 3–9‍ ‍min, 98% solvent B. After separation, C16-HSL was detected and quantified using the multiple reaction monitoring mode as follows: C16-HSL was fragmented into a product ion with an *m/z* value of 102.5, and this product ion was detected in the positive ion mode. The amount of C16-HSL in the sample was calculated using linear standard curves for the ion counts of the product ion with *m/z* 102.5, and the concentrations of synthetic C16-HSL (0–10‍ ‍μM). Samples were analyzed in triplicate. C16-HSL concentrations were calculated as the mean value of triplicates.

## Results and Discussion

### DNA-damaging stress triggers MV formation in *P. denitrificans* through cell lysis

**Fig. 1.**
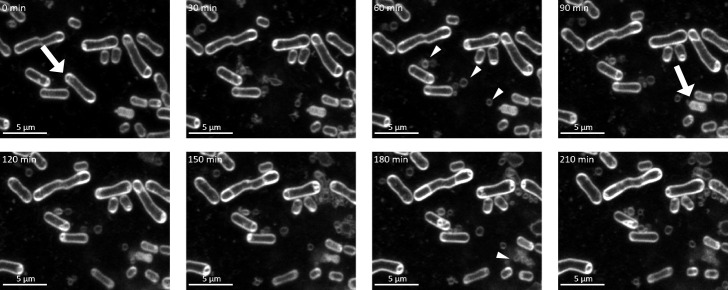
Time-lapse microscopy of *Paracoccus denitrificans* Pd1222 WT treated with 50‍ ‍ng mL^–1^ MMC. White arrows indicate lysed cells. White triangles indicate membrane vesicles (MVs) that were produced as a result of cell lysis. The numbers in the upper left corner indicate the elapsed time (min) when the time point at the beginning of imaging was 0. White: FM4-64 (membrane); Scale bar: 5‍ ‍μm.

We initially investigated the production of MVs under the MMC treatment using super-resolution live-cell imaging. Live-cell imaging is a powerful approach for investigating the process of MV formation (Pirbadian *et al.*, 2014; Turnbull *et al.*, 2016; Toyofuku *et al.*, 2017a). In the present study, MMC and FM4-64 were simultaneously added to the cell culture, and cells were placed on a TSB agar pad containing MMC and FM4-64 to stain the bacterial membrane. We observed that a subpopulation of cells produced MVs through cell lysis (Fig. 1, Fig. S1A, and Movie 1). A previous study reported that endolysin triggered explosive cell lysis in *P. aeruginosa* under DNA-damaging stress, during which cells became round in shape and exploded, resulting in a shattered membrane for the formation of MVs (Turnbull *et al.*, 2016). In contrast, round cells were only observed in *P. denitrificans* Pd1222 under the conditions used, and rod cells were found to explode and release MVs without forming round cells. When different fractions of ultracentrifuged MV samples were inspected using TEM (Fig. S1B), the upper fraction (fraction I) was found to contain many MVs, whereas the lower fraction (fraction II) contained phage-like particles (Fig. S1C).

### Prophage genes are enriched in *P. denitrificans* Pd1222 MVs

Previous studies demonstrated that holin and endolysin, which trigger MV formation, are encoded in the prophage region (Turnbull *et al.*, 2016; Toyofuku *et al.*, 2017a; Nagakubo *et al.*, 2021). Therefore, we searched the prophage regions of the *P. denitrificans* Pd1222 genome for holin and endolysin genes (*hol* and *lys)* using *PhiSpy*, a bioinformatics tool that searches for prophages within bacterial genomes (Akhter *et al.*, 2012). *P. denitrificans* has two chromosomes (CP000489.1: 2,852,282 bp and CP000490.1: 1,730,097 bp) and a large plasmid (CP000491.1: 653,815 bp). The *PhiSpy* ana­lysis revealed four putative prophage regions on the chromosomes (Table 1). The gene, Pden_0381, encoding a protein that possesses a peptidoglycan degradation domain, a characteristic function of endolysin, was observed in Region 1 (Fig. S2A). Pden_0381 encodes a putative peptidoglycan-binding domain (E-value=0.024) and an *N*-acetylmuramoyl-**l**-alanine amidase domain (E-value=8.1e-11), as identified using the KEGG sequence similarity database (Fig. S2B). Since *lys* and *hol* have often been found in pairs in the genome, we analyzed Pden_0382, the adjacent gene of Pden_0381, using TMHMM server version 2.0 (Moller *et al.*, 2001). Pden_0382 was estimated to have a single transmembrane domain (Fig. S2C), which is a typical feature of Class III holins (Young, 2002). These *lys* and *hol* sequences were not found in Regions 2-4.

**Fig. 2.**
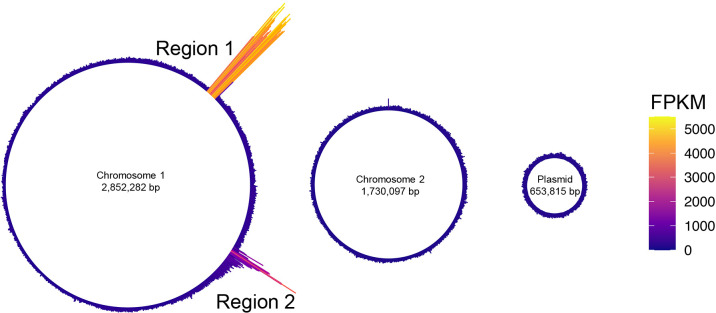
Circular plot of the distribution of averaged DNA-seq reads in MVs. Circular plot showing the distribution of mapped DNA-seq reads on two chromosomes and a plasmid of *Paracoccus denitrificans* Pd1222. Color indicates the height of FPKM.

**Fig. 3.**
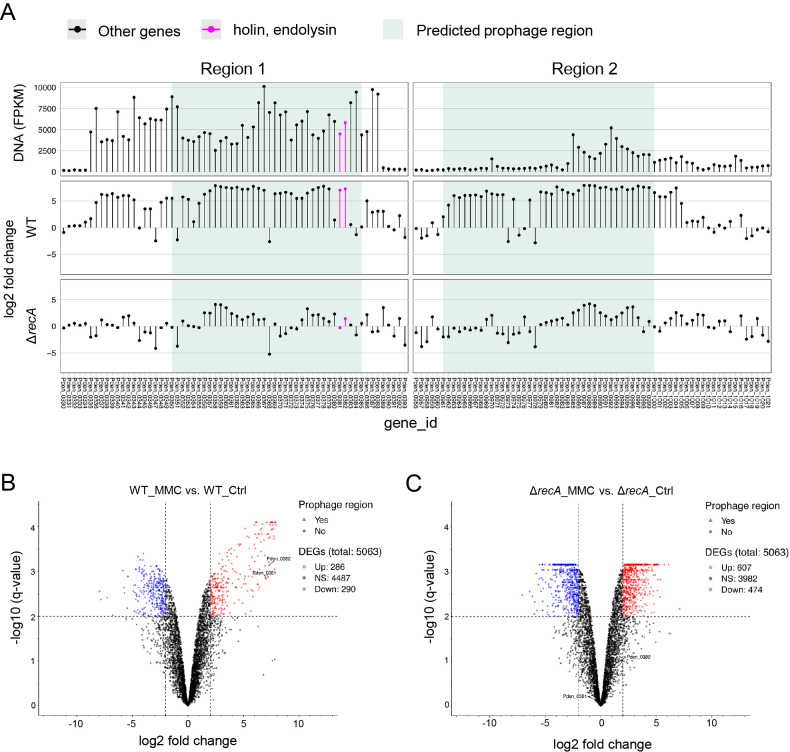
Induction of Pden_0381 and Pden_0382 revealed by DNA-seq and RNA-seq. (A) Green shading indicates regions 1 and 2 as prophage regions predicted using *PhiSpy*. Dots represent the transcripts of each gene. (B, C) Volcano plot showing the results of an ana­lysis of differential expression between (B) WT with MMC *vs.* WT without MMC and (C) Δ*recA* with MMC *vs.* Δ*recA* without MMC. The vertical axis (y-axis) corresponds to the mean expression value of log 10 (q-value), and the horizontal axis (x-axis) represents the log_2_ fold-change value. Genes above the thresholds have been highlighted in color: red and blue dots represent transcripts whose expression was up-regulated and down-regulated, respectively. The black dots indicate no significant change, positive x-values represent up-regulation, and negative x-values represent down-regulation. Lists of genes are provided in Tables S3 and S4.

Previous studies indicated that the content of MVs reflects the physiology of a cell (Toyofuku *et al.*, 2012; Turnbull *et al.*, 2016). Since prophage regions are often excised and replicated during the formation of phage particles, we hypothesized that by examining the DNA enriched in MVs, we may identify the phage region induced when MVs are formed (Gaudin *et al.*, 2014). The DNA-seq of the MVs isolated from MMC-treated cells revealed the accumulation of prophage Regions 1 and 2 in MVs (Fig. 2). The peak detected in Region 1 showed a continuous increase in the number of reads, covering all of the prophage regions predicted by *PhiSpy* (Fig. 3A). In contrast, only parts of Region 2 were enriched in MVs.

### Prophage genes are triggered in response to the MMC treatment

To identify the genes regulated upon DNA damage and those involved in MV formation, we conducted RNA-seq of cells exposed to MMC. RNA was isolated from cells treated with or without MMC. In total, 286 genes were up-regulated and 290 were down-regulated in response to MMC. As expected, many genes derived from the prophage region were up-regulated upon the addition of MMC (Table S3, Fig. 3A and B). We also found that the gene expression of Pden_0381 and Pden_0382, as described above, was significantly up-regulated.

**Fig. 4.**
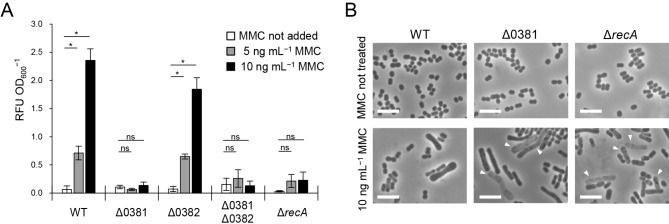
Genotoxic stress induces MV production and cell elongation. (A) MV production in *Paracoccus denitrificans* Pd1222 WT and isogenic mutants was analyzed after an incubation with or without MMC. *n*=3; mean±s.d. *: *P*<0.05, ns: *P*≥0.05 (Welch’s *t*-test). (B) Phase-contrast micrographs showing cell morphologies in response to MMC. White triangles indicate ghost cells with a decrease in phase contrast. Scale bar, 5‍ ‍μm.

*recA* is generally involved in prophage induction in other bacteria (Kidambi *et al.*, 1996; Simmons *et al.*, 2008; Binnenkade *et al.*, 2014), and Δ*recA* of *P. denitrificans* Pd1222 did not induce MV formation when treated with MMC (Toyofuku *et al.*, 2017b) (Fig. 4A). We also analyzed the transcriptome of Δ*recA* under the MMC treatment. Consistent with previous results, the expression of prophage genes, including Pden_0381 and Pden_0382, did not increase in Δ*recA* when treated with MMC (Table S4, Fig. 3A and C). Collectively, these results suggest that the expression of endolysin (Pden_0381) and holin (Pden_0382) was up-regulated by RecA upon the MMC treatment and triggered MV formation.

### Prophage genes are responsible for MV production under DNA-damaging stress

To establish whether Pden_0381 and Pden_0382 are involved in MV formation, we constructed mutants of these genes and measured their MV production. In contrast to WT, ΔPden_0381 did not show a dose-dependent increase in MV production (Fig. 4A). ΔPden_0382 showed an increase in MV production; however, the amount of MV in ΔPden_0382 was less than that in WT. These results indicate that Pden_0382 is partially functional — in contrast to Pden_0381, which is essential for MV formation under DNA-damaging stress. Nevertheless, the prophage genes that were crucial for MV induction under DNA-damaging stress, namely, ΔPden_0381, ΔPden_0382, and Δ*recA*, showed no significant change in MV production from that in WT when MMC was not added (*P*>0.05) (Fig. 4). These results suggest that MVs are formed in *P. denitrificans* through various mechanisms that depend on the surrounding conditions. Recent studies reported that multiple routes for the formation of MVs lead to differences in MV components (Mashburn and Whiteley, 2005; Perez-Cruz *et al.*, 2013; Turnbull *et al.*, 2016; Toyofuku *et al.*, 2017a). We analyzed the protein profiles of MVs produced with and without MMC using SDS-PAGE (Fig. S3) and attempted to identify characteristic protein bands that are specific to MMC-induced MVs by MALDI-TOF MS (Table S5). The results obtained showed that MMC-induced MVs contained typical inner membrane proteins associated with the respiratory chain (Fig. S3 and Table S5, Bands A, B, and C). The presence of inner membrane proteins is indicative of outer-inner MVs, which may also form upon the addition of a DNA-damaging agent in *Stenotrophomonas maltophilia* (Devos *et al.*, 2017), presumably through cell lysis.

Following the addition of MMC, ΔPden_0381 and Δ*recA* showed more “ghost” cells than WT (Fig. 4B, white arrows). Ghost cells are dead or dying cells characterized by a decrease in phase contrast due to the leakage of cytoplasmic components (Faridani *et al.*, 2006; Pospisil *et al.*, 2020). Along with the results shown in Fig. 1 and Movie 1, the absence of ghost cells in WT may be evidence for cell lysis by endolysin. In contrast, the ghost cells of ΔPden_0381 and Δ*recA* remained and, thus, did not produce many MVs under DNA-damaging stress. In addition, cell elongation was observed even in the Δ*recA* strain in response to MMC, which indicated that cell elongation is not regulated by RecA in this bacterium (Fig. 4B), in contrast to that in other bacteria whose cell elongation is regulated by RecA (Ogino *et al.*, 2008; Keyamura *et al.*, 2013).

### Pden_0381 and Pden_0382 are both required to trigger MV production

**Fig. 5.**
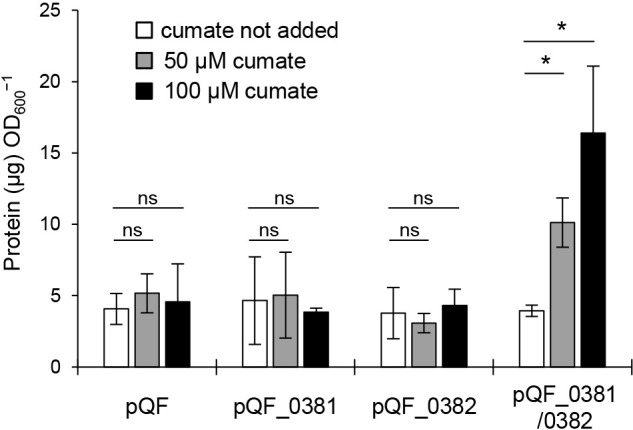
Gene expression of Pden_0381 and Pden_0382 leads to MV production. MV production was quantified after an incubation with or without cumate. *n*=3; mean±s.d. *: *P*<0.05, ns: *P*≥0.05 (Welch’s *t*-test).

To confirm that Pden_0381 and Pden_0382 induce MV production, we expressed these genes under an inducible promoter using pQF. pQF contains the cym/cmt system from *Pseudomonas putida* F1 and induces the expression of target genes upon the addition of cumate into the cell culture (Kaczmarczyk *et al.*, 2013). The induction of Pden_0381 or Pden_0382 alone did not increase MV production (Fig. 5). In contrast, MV production increased when the expression of Pden_0381 and Pden_0382 was induced in the same cell. These results indicate that holin is vital for endolysin to trigger MV formation. This is consistent with previous findings showing that holin was required for the translocation of endolysin into the periplasmic space (Young and Blasi, 1995). Although Pden_0381-deficient cells lacked the ability to induce MV production in response to the addition of MMC, Pden_0382-deficient cells only exhibited a partial reduction in their ability to induce MV production (Fig. 4A). This result implies that there are multiple holins in *P. denitrificans* Pd1222, which is consistent with previous findings on *P. aeruginosa* (Hynen *et al.*, 2021).

### C16-HSL is released by the prophage gene via MV formation

We previously revealed that C16-HSL accumulated in cells and MVs in *P. denitrificans* and was trafficked between cells *via* MVs (Toyofuku *et al.*, 2017b). Based on the results shown in Fig. 1 and Movie 1, *P. denitrificans* released MVs through cell lysis under DNA-damaging stress. Therefore, we hypothesized that increased MV production *via* prophage genes may promote C16-HSL release into the supernatant and further stimulate bacterial communication. To investigate this possibility, we measured the amount of C16-HSL in the supernatant after the addition of MMC and compared it with that in the mutants.

We initially performed a C16-HSL bioassay using the long-chain AHL reporter strain, *C. violaceum* VIR24/pPROBE-P*vioA*-gfp (Toyofuku *et al.*, 2017b). The cell-free supernatant was obtained from the final cell culture following the treatment with MMC and was then added to the reporter strains. ΔPden_0381 and Δ*recA* showed no significant changes in C16-HSL concentrations in the supernatant with or without MMC (Fig. S4). In contrast, WT released more C16-HSL into the supernatant in response to MMC. This increase in C16-HSL concentrations in the supernatant corresponded to MV production under DNA-damaging stress (Fig. 4A).

**Fig. 6.**
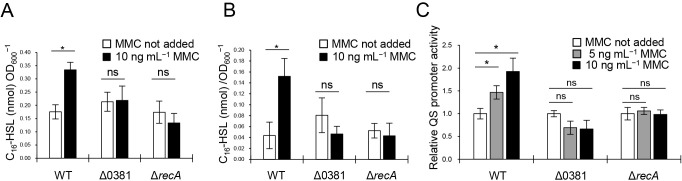
Genotoxic stress induces C16-HSL release into the supernatant through a prophage gene (A, B) HPLC-MS ana­lysis of C16-HSL extracted from (A) total supernatants or (B) MVs. *n*=3; mean±s.d. *: *P*<0.05, ns: *P*≥0.05 (Welch’s *t*-test). (C) Bioassay for QS promoter activity. The AHL biosensor *Paracoccus denitrificans* Pd1222Δ*pdnI*Δ*pxm*/pPLlas was cultivated using the total supernatant of *P. denitrificans* Pd1222 cultured with or without MMC. Results are expressed as a fold change in fluorescence intensity relative to that in MMC-untreated cells. *n*=3; mean±s.d. *: *P*<0.05, ns: *P*≥0.05 (Welch’s *t*-test).

We further fractionated the supernatant and examined the localization of C16-HSL by quantifying it with HPLC. After an incubation with or without MMC, supernatants were collected and MVs were isolated. In accordance with the results shown in Fig. S4, more C16-HSL was detected in the total supernatant (supernatant containing MVs) of WT treated with MMC than in that of untreated WT (Fig. 6A). An increase in C16-HSL levels was also observed in the MV fraction (Fig. 6B). This increase in C16-HSL was not detected in the ΔPden_0381 or Δ*recA* mutants, indicating that the release of C16-HSL into the supernatant was triggered by MV formation through endolysin. C16-HSL concentrations in the supernatant were lower than those in our previous study (Toyofuku *et al.*, 2017b), which may be attributed to the different culture conditions employed.

We also examined whether the QS promoter activity of *P. denitrificans* was stimulated by inducing MV formation. The supernatant of *P. denitrificans* was collected and added to an AHL reporter strain of *P. denitrificans*. Accordingly, QS promoter activity in the reporter strain increased with the addition of the WT supernatant in proportion to the amount of MMC (Fig. 6C). This was not observed when the supernatant was collected from ΔPden_0381 or Δ*recA*, which suggested that MVs released by the action of endolysin triggered the QS response in *P. denitrificans*.

Collectively, the present results demonstrated that the release of QS signals may be stimulated by a prophage gene through the formation of MVs. A recent study reported that virulent phages also induce the formation of MVs in *E. coli* (Mandal *et al.*, 2021). This has yet to be confirmed in *P. denitrificans* Pd1222, but implies that C16-HSL may be released not only by triggering the prophage gene, but also by phage infection *via* MVs. Recent studies reported that phages communicate through signaling molecules to control other phage populations. Silpe and Bassler demonstrated that VP882 influenced the lysis-lysogeny fate by recognizing host-produced QS signaling molecules in *Vibrio cholerae* (Silpe and Bassler, 2019). Furthermore, Erez *et al.* revealed a phage-to-phage communication system called the arbitrium system (Erez *et al.*, 2017). Although it currently remains unclear whether the promotion of the QS cascade by a prophage gene in *P. denitrificans* Pd1222 benefits bacteria or phages, the present study provides further insights into the interplay between phages and bacteria. Moreover, given their abundance in nature, our results imply that phages play an important role in MV-mediated bacterial interactions.

## Citation

Yasuda, M., Yamamoto, T., Nagakubo, T., Morinaga, K., Obana, N., Nomura, N., and Toyofuku, M. (2022) Phage Genes Induce Quorum Sensing Signal Release through Membrane Vesicle Formation. *Microbes Environ ***37**: ME21067.

https://doi.org/10.1264/jsme2.ME21067

## Supplementary Material

Supplementary Material

## Figures and Tables

**Table 1.** List of predicted prophage regions in *Paracoccus denitrificans* Pd1222.

Putative prophage region	Region length (bp)	Region position
Chromosome 1 (CP000489.1)		
Region 1	28,984	330,301 to 359,285 bp
Region 2	31,029	948,709 to 979,738 bp
Chromosome 2 (CP000490.1)		
Region 3	12,819	49,990 to 62,809 bp
Region 4	28,242	876,103 to 904,345 bp
