# Supplementary material for: Phage Genes Induce Quorum Sensing Signal Release through Membrane Vesicle Formation
Source: Microbes Environ. 2022 Jan 27;37(1):ME21067. doi: 10.1264/jsme2.ME21067 (PMC8958291; doi:10.1264/jsme2.ME21067)
Supplement: Supplementary file 1 — Supplementary Material [file 37_21067_s1.pdf]

Table S1. List of strains and plasmids used in this study.

Strains or Plasmids	Relevant characteristics	Source or Reference
**Strain**		
Paracoccus denitrificans		
Pd1222	Wild type	Devries *et. al.* 1989
ΔPden_0381	Pden_0381 deletion mutant	This study
ΔPden_0382	Pden_0382 deletion mutant	This study
ΔPden_0381ΔPden_0382	deletion mutant of Pden_0381 and Pden_0382	This study
Δ*recA*	*recA* (Pden_0597) deletion mutant	Toyofuku *et. al.* 2017
ΔPden_0381ΔPden_0382/pQF	ΔPden_0381ΔPden_0382 carrying pQF	This study
ΔPden_0381ΔPden_0382 /pQF_0381	ΔPden_0381ΔPden_0382 carrying pQF_0381	This study
ΔPden_0381ΔPden_0382 /pQF_0382	ΔPden_0381ΔPden_0382 carrying pQF_0382	This study
ΔPden_0381ΔPden_0382 /pQF_0381_0382	ΔPden_0381ΔPden_0382 carrying pQF_0381_0382	This study
Chromobacterium violaceum		
VIR24	*cviI* deletion mutant of ATCC12472	Someya *et. al.* 2009
VIR24/pPROBE-P*vioA*-gfp	VIR24 carrying pPROBE-P*vioA*-gfp	Toyofuku *et. al.* 2017
Escherichia coli		
DH5α	Cloning host	TaKaRa
S17-1	Mobilizer strain for conjugation	Simon *et. al.* 1986
**Plasmids**		
pK18mobsacB	Allelic exchange suicide vector; *sacB* Km^R	Schäfer *et. al.* 1994
pK18mobsacB-ΔPden_0381	Pden_0381 deletion cassette in pK18mobsacB	This study
pK18mobsacB-ΔPden_0382_WT	Pden_0382 deletion cassette for WT in pK18mobsacB	This study
pK18mobsacB-ΔPden_0382_ΔPden_0381	Pden_0382 deletion cassette for ΔPden_0381 in pK18mobsacB	This study
pQF	Cumate-inducible vector; Tet^R	Kaczmarczyk *et. al.* 2013
pQF_0381	pQF derivative to induce Pden_0381	This study
pQF_0382	pQF derivative to induce Pden_0382	This study

pQF_0381_0382	pQF derivative to induce Pden_0381 and Pden_0382	This study
pPROBE-P*vioA*-gfp	AHL reporter plasmid	Toyofuku *et. al.* 2017

Table S2. Sequence of primers used in this study.

Primer	Sequence 5'→ 3' (restriction enzyme sites are underlined)	Source or Reference
**For gene deletion**		
0381F1	ACTTTTGGCTCACGCCTCTA	This study
0381R1	CTGCATGATGATGCTCCTCAGTCGATTCCGTCGATAGGAC	This study
0381F2	GTCCTATCGACGGAATCGACTGAGGAGCATCATCATGCAG	This study
0381R2	GCCACCAAAGTCGAAAACAT	This study
0381overF	GCCAAGCTTCTTTGGGCCAAGTTCAATTC	This study
0381overR	TACGAATTCCAACTGATCGACGCCAACTG	This study
0382wtF1	AAGATCAAGACCAGCGCGAG	This study
0382wtR1	GCATCACGCCGCCCCTGCCGCGTTGATGATAGTCTGCA	This study
0382lysF1	GCAGTTCTTCGGGACCTTC	This study
0382lysR1	GCATCACGCCGCCCCTGCCGCGTTGATGATAGTCTGCA	This study
0382F2	TGCAGACTATCATCAACGCGGCAGGGGCGGCGTGATGC	This study
0382R2	ATCGTCTCGGATTCGCTCTG	This study
0382wtoverF	CCCAAGCTTTCGACCTCTGGCGATATTGG	This study
0382lysoverF	CCCAAGCTTCAGGATACCACCACCACGAC	This study
0382overR	CCGGAATTCAGCAGGGCGGTAAATGATCC	This study
**For inducible plasmid**		
Ind0381F	TGACTCTAGAGTGATTTTTTCATGGAGAGATGAC	This study
Ind0381R	GCTCGGTACCTCAGAAGGTGGAAAGCTCGC	This study
Ind0382F	TGACTCTAGAGTGAACCTTCTGAGGAGCATCATC	This study
Ind0381R	GCTCGGTACCTCACGCCGCCCCTGCCCGCC	This study
Ind0381_0382F	TGACTCTAGAGTGATTTTTTCATGGAGAGATGAC	This study
Ind0381_0382R	GCTCGGTACCTCACGCCGCCCCTGCCCGCC	This study

Table S3. Lists of differentially expressed genes between WT with MMC vs. WT without MMC.

gene_id	name	product	PhiSpy_phage_region	logFC	PValue	FDR
Pden_0002	*rho*	transcription termination factor Rho		1.90	7.81E-04	0.005794672
Pden_0003	*mnmE (trmE)*	tRNA modification GTPase trmE		2.39	2.60E-05	0.001142137
Pden_0010	*rph*	RNAse PH		1.39	4.32E-04	0.004092226
Pden_0011	*rdgB*	dITPase		1.98	0.001490927	0.008797857
Pden_0012		Endoribonuclease L-PSP		1.73	2.70E-04	0.003200601
Pden_0013		putative oxygen-independent coproporphyrinogen III oxidase		1.42	5.54E-04	0.004780002
Pden_0015	*gfa*	glutathione-dependent formaldehyde-activating, GFA		-2.03	0.001314887	0.008082965
Pden_0020		Pyrrolo-quinoline quinone		-5.77	1.40E-04	0.002302411
Pden_0021		hypothetical protein		-4.68	1.63E-05	8.87E-04
Pden_0022		amino acid ABC transporter substrate-binding protein, PAAT family		-3.98	4.74E-05	0.001471351
Pden_0023		rhodanese		-3.46	5.84E-05	0.001616929
Pden_0024		conserved hypothetical protein		-2.50	2.95E-05	0.001194403
Pden_0026		amino acid/amide ABC transporter substrate-binding protein, HAAT family		-4.39	2.09E-05	9.98E-04
Pden_0027		40-residue YVTN family beta-propeller repeat protein		-4.05	2.08E-05	9.98E-04
Pden_0028		hypothetical protein		-4.64	2.69E-04	0.003197585
Pden_0029		ABC transporter related protein		-3.27	1.08E-04	0.002099137
Pden_0030		ABC-2 type transporter		-2.63	1.92E-04	0.002717725
Pden_0031		conserved hypothetical protein		-1.61	5.76E-04	0.004898771
Pden_0048		AMP-dependent synthetase and ligase		-5.88	1.83E-05	9.57E-04
Pden_0049		amino acid/amide ABC transporter ATP-binding protein 1, HAAT family		-4.50	5.52E-06	4.66E-04
Pden_0050		amino acid/amide ABC transporter membrane protein 1, HAAT family		-3.53	1.12E-04	0.00214351
Pden_0051		amino acid/amide ABC transporter membrane protein 2, HAAT family		-3.21	6.62E-04	0.005254598
Pden_0052		amino acid/amide ABC transporter substrate-binding protein, HAAT family		-4.14	2.45E-05	0.001107056
Pden_0053		amino acid/amide ABC transporter ATP-binding protein 2, HAAT family		-3.94	1.78E-05	9.49E-04
Pden_0054		phenylacetate-CoA ligase, putative		-2.55	1.05E-04	0.002060708
Pden_0063	*msrP*	oxidoreductase, molybdopterin binding protein		-1.38	0.001265593	0.007871864
Pden_0067	*tnpB*	IS66 Orf2 family protein		4.28	1.21E-04	0.002215935
Pden_0068		transposase IS66		2.56	7.86E-05	0.001836392
Pden_0077		transposase		1.40	0.001570019	0.009043238
Pden_0087	*pseF*	acylneuraminate cytidylyltransferase		1.41	0.001210589	0.007644393
Pden_0088		flagellin modification protein FlmD		1.33	9.85E-04	0.006721522
Pden_0098		Conjugal transfer TraD family protein		-1.63	9.56E-04	0.006665173
Pden_0099		conserved hypothetical protein		-1.46	6.37E-04	0.005142188
Pden_0116	*catB*	transferase hexapeptide repeat containing protein		2.44	4.18E-05	0.001440403
Pden_0126		hypothetical protein		1.94	6.33E-04	0.005126214
Pden_0134		transposase IS116/IS110/IS902 family protein		-2.15	3.07E-05	0.001223584
Pden_0160		acriflavin resistance protein		1.34	5.36E-04	0.00465373
Pden_0171		monooxygenase, FAD-binding protein		-3.00	6.89E-04	0.005364018
Pden_0172		benzoate-CoA ligase family		-2.78	4.30E-04	0.004090524
Pden_0174	*maiA*	maleylacetoacetate isomerase		-2.45	9.11E-04	0.006432915
Pden_0176		amino acid/amide ABC transporter membrane protein 1, HAAT family		-2.32	0.001612855	0.009144328
Pden_0177		amino acid/amide ABC transporter membrane protein 2, HAAT family		-2.49	0.001348882	0.008228178
Pden_0179		amino acid/amide ABC transporter ATP-binding protein 2, HAAT family		-2.39	7.42E-04	0.005632016
Pden_0180		aldehyde dehydrogenase		-3.10	1.15E-04	0.002162073
Pden_0181		glucose-methanol-choline oxidoreductase		-3.29	4.39E-04	0.004136616
Pden_0182		RNAse Z		-3.36	1.77E-04	0.002605071
Pden_0185		conserved hypothetical protein		-1.28	7.43E-04	0.005632016
Pden_0187		transcriptional regulator, MarR family		-1.80	1.34E-04	0.002284871
Pden_0189		TRAP C4-dicarboxylate transport system permease DctM subunit		-2.58	1.81E-04	0.002630055
Pden_0190		Tripartite ATP-independent periplasmic transporter, DctQ component		-3.28	1.38E-04	0.002302411
Pden_0192		short-chain dehydrogenase/reductase SDR		-3.26	7.97E-05	0.001841466
Pden_0193		TRAP dicarboxylate transporter- DctP subunit		-4.50	5.27E-04	0.004592119
Pden_0194		conserved hypothetical protein		-2.26	8.85E-05	0.001940531
Pden_0195		L-carnitine dehydratase/bile acid-inducible protein F		-2.21	4.30E-04	0.004090524
Pden_0203		luciferase family protein		-2.71	1.52E-05	8.86E-04
Pden_0206		short-chain dehydrogenase/reductase SDR		-2.37	2.97E-04	0.003369347
Pden_0220		luciferase family protein		-1.66	6.20E-04	0.005085218
Pden_0224		sigma54 specific transcriptional regulator, Fis family		-2.61	6.41E-05	0.001689635
Pden_0233		membrane protein-like protein		2.00	7.33E-05	0.001802006
Pden_0235		oligopeptide/dipeptide ABC transporter, ATPase subunit		-2.40	7.86E-05	0.001836392
Pden_0236		oligopeptide/dipeptide ABC transporter, ATPase subunit		-2.64	2.64E-05	0.001142137
Pden_0237		binding-protein-dependent transport systems inner membrane component		-3.24	3.29E-04	0.003583012
Pden_0238		binding-protein-dependent transport systems inner membrane component		-3.68	1.01E-04	0.00205033
Pden_0239		extracellular solute-binding protein, family 5		-3.76	2.02E-04	0.002752118
Pden_0241		(R)-2-hydroxyacid dehydrogenase		-1.73	2.18E-04	0.002830827
Pden_0242		iron-containing alcohol dehydrogenase		-3.03	3.51E-05	0.001297726
Pden_0243		succinate semialdehyde dehydrogenase		-2.06	0.001108637	0.007205428
Pden_0244		Mandelate racemase/muconate lactonizing enzyme, C-terminal domain protein		-2.14	2.03E-04	0.002760804
Pden_0245		5-carboxymethyl-2-hydroxymuconate delta-isomerase		-1.93	3.40E-04	0.00362723
Pden_0270		gene transfer agent (GTA) like protein		1.14	0.00153829	0.008962304
Pden_0280	*ehuA*	hydroxyectoine ABC transporter ATP-binding protein / ectoine ABC transporter ATP-binding protein		-3.32	0.001180755	0.007510254
Pden_0281	*ehuB*	hydroxyectoine-binding protein / ectoine-binding protein		-3.32	1.83E-04	0.00264125
Pden_0282	*ehuC*	hydroxyectoine ABC transporter membrane protein / ectoine ABC transporter membrane protein		-3.60	1.15E-04	0.002162073
Pden_0283	*ehuD*	hydroxyectoine ABC transporter membrane protein / ectoine ABC transporter membrane protein		-2.96	2.63E-04	0.003161243
Pden_0284		Asp/Glu racemase		-2.41	0.001005012	0.006795278
Pden_0285	*eutB*	Pyridoxal-5'-phosphate-dependent enzyme, beta subunit		-2.33	5.31E-04	0.004618534
Pden_0286		Ornithine cyclodeaminase		-2.64	9.23E-04	0.006496172
Pden_0287	*doeA*	peptidase M24		-2.79	6.40E-04	0.005146542

Pden_0290		aminotransferase		-2.29	6.83E-04	0.005345115
Pden_0296		TonB-dependent receptor		-1.88	2.19E-04	0.002830827
Pden_0303		transmembrane pair domain protein		-3.53	0.00184495	0.009969029
Pden_0304		transcriptional regulator, LysR family		3.07	5.99E-04	0.004987227
Pden_0314		AmiS/UreI transporter		1.19	0.001051461	0.006987997
Pden_0319		conserved hypothetical protein		-2.06	0.001046144	0.006969248
Pden_0337		conserved hypothetical protein		6.24	1.33E-06	1.77E-04
Pden_0338		hypothetical protein		6.06	2.82E-06	3.04E-04
Pden_0339		hypothetical protein		6.38	9.15E-07	1.45E-04
Pden_0340		hypothetical protein		5.68	1.68E-04	0.002556934
Pden_0341		DNA methylase N-4/N-6 domain protein		6.02	7.92E-06	5.99E-04
Pden_0342		DNA polymerase III beta subunit family protein		5.98	2.12E-06	2.47E-04
Pden_0343		hypothetical protein		5.19	8.29E-05	0.001890849
Pden_0345		hypothetical protein		3.55	3.94E-06	3.76E-04
Pden_0346		hypothetical protein		3.54	2.01E-05	9.98E-04
Pden_0348		hypothetical protein		4.74	2.20E-06	2.47E-04
Pden_0349		hypothetical protein		5.52	2.92E-06	3.07E-04
Pden_0350		hypothetical protein	1	5.50	1.25E-06	1.72E-04
Pden_0352		hypothetical protein	1	5.76	5.90E-06	4.82E-04
Pden_0353		phage/plasmid primase, P4 family	1	5.32	1.12E-06	1.63E-04
Pden_0355		hypothetical protein	1	4.57	2.20E-06	2.47E-04
Pden_0356		HNH endonuclease	1	6.28	8.74E-05	0.001937938
Pden_0357		hypothetical protein	1	6.91	4.88E-06	4.34E-04
Pden_0358		Resolvase helix-turn-helix domain protein	1	7.86	2.16E-07	7.94E-05
Pden_0359		phage Terminase	1	7.70	4.78E-07	1.10E-04
Pden_0360		phage portal protein, HK97 family	1	7.53	1.72E-07	7.94E-05
Pden_0361		peptidase S14, ClpP	1	7.40	3.20E-07	9.06E-05
Pden_0362		phage major capsid protein, HK97 family	1	7.59	2.17E-07	7.94E-05
Pden_0363		hypothetical protein	1	7.21	3.65E-05	0.001320801
Pden_0364		hypothetical protein	1	7.23	9.42E-07	1.45E-04
Pden_0365		hypothetical protein	1	7.75	1.08E-06	1.61E-04
Pden_0366		phage head-tail adaptor, putative	1	7.37	1.26E-06	1.72E-04
Pden_0367		hypothetical protein	1	6.99	4.16E-05	0.001440403
Pden_0369		hypothetical protein	1	6.37	1.69E-04	0.002556934
Pden_0370		hypothetical protein	1	6.44	1.62E-05	8.87E-04
Pden_0371		hypothetical protein	1	6.66	8.95E-07	1.45E-04
Pden_0372		hypothetical protein	1	6.39	8.95E-07	1.45E-04
Pden_0373		hypothetical protein	1	5.49	3.28E-05	0.001268121
Pden_0374		hypothetical protein	1	5.51	1.06E-05	7.37E-04
Pden_0375		conserved hypothetical protein	1	6.46	1.30E-04	0.002284717
Pden_0376		hypothetical protein	1	7.15	7.81E-07	1.45E-04
Pden_0377		hypothetical protein	1	7.53	5.49E-07	1.15E-04
Pden_0378		hypothetical protein	1	7.76	1.46E-06	1.84E-04
Pden_0379		hypothetical protein	1	7.27	1.41E-06	1.83E-04
Pden_0381		hypothetical protein	1	7.02	4.51E-05	0.001444301
Pden_0382		hypothetical protein	1	7.28	1.08E-05	7.37E-04
Pden_0386		protein of unknown function DUF159		5.03	8.29E-05	0.001890849
Pden_0387		hypothetical protein		2.88	4.38E-06	3.97E-04
Pden_0388		hypothetical protein		3.10	1.23E-05	7.80E-04
Pden_0392		hypothetical protein		2.26	3.20E-04	0.003546894
Pden_0396	*mtaB*	MiaB-like tRNA modifying enzyme		1.19	0.001499277	0.008820336
Pden_0400	*dxs*	1-deoxy-D-xylulose-5-phosphate synthase		1.35	0.001403645	0.008430197
Pden_0407		glutathione S-transferase		1.84	0.001612539	0.009144328
Pden_0415		outer membrane protein		1.10	0.001708494	0.009526546
Pden_0462		IstB domain protein ATP-binding protein		-1.16	0.001340148	0.008184762
Pden_0464		transposase IS116/IS110/IS902 family protein		-1.55	5.88E-04	0.004960806
Pden_0472		5-oxoprolinase (ATP-hydrolyzing)		-2.50	4.32E-05	0.001441542
Pden_0473		Hydantoinase B/oxoprolinase		-2.31	6.50E-05	0.001696353
Pden_0474		short-chain dehydrogenase/reductase SDR		-2.22	4.41E-05	0.001441542
Pden_0475		hypothetical protein		-2.08	2.17E-04	0.002830827
Pden_0476		thioesterase superfamily protein		-1.66	0.00166566	0.009359866
Pden_0495		response regulator receiver protein		1.49	9.67E-04	0.006681372
Pden_0498		hypothetical protein		2.14	7.13E-04	0.005462944
Pden_0503		ribose ABC transporter ATP-binding protein / fructose ABC transporter ATP-binding protein / mannose ABC transporter ATP-binding protein		-3.73	2.87E-05	0.001183343
Pden_0504		fructose ABC transporter membrane protein / mannose ABC transporter membrane protein / ribose ABC transporter membrane protein		-4.43	1.34E-04	0.002284871
Pden_0505		mannose-binding protein / fructose-binding protein / ribose-binding protein		-4.16	2.30E-05	0.001068039
Pden_0528		nucleoside-binding protein		-2.18	1.57E-04	0.002451077
Pden_0538	*glyQ*	glycyl-tRNA synthetase alpha chain		1.75	3.46E-04	0.003644311
Pden_0539		signal transduction histidine kinase regulating citrate/malate metabolism		1.60	6.66E-04	0.005274631
Pden_0540	*glyS*	glycyl-tRNA synthetase beta chain		1.99	1.03E-04	0.002056763
Pden_0546		monooxygenase, FAD-binding protein		1.66	5.94E-04	0.004969545
Pden_0575	*gatC*	aspartyl/glutamyl-tRNA(Asn/Gln) amidotransferase subunit C		1.07	0.001726452	0.009573963
Pden_0576	*gatA*	aspartyl/glutamyl-tRNA(Asn/Gln) amidotransferase subunit A		1.28	0.001748308	0.009652871
Pden_0579	*rlmJ*	protein of unknown function DUF519		1.44	4.96E-04	0.0044545
Pden_0587	*murF*	UDP-N-acetylmuramoyl-tripeptide--D-alanyl-D-alanine ligase		1.44	9.57E-04	0.006665586
Pden_0588	*mraY*	Phospho-N-acetylmuramoyl-pentapeptide-transferase		1.34	5.37E-04	0.00465373
Pden_0589	*murD*	UDP-N-acetylmuramoylalanine--D-glutamate ligase		1.67	0.00183284	0.009924778
Pden_0590		membrane protein involved in aromatic hydrocarbon degradation		-3.97	9.85E-04	0.006721522
Pden_0592	*guaB*	inosine-5'-monophosphate dehydrogenase		1.24	0.00108132	0.00710081
Pden_0593		hypothetical protein		2.33	3.22E-05	0.001255634

Pden_0601		hypothetical protein		4.56	4.92E-05	0.001490678
Pden_0602		hypothetical protein		4.18	6.31E-04	0.005126214
Pden_0603	*purA*	Adenylosuccinate synthetase		1.77	2.09E-04	0.00277756
Pden_0604		conserved hypothetical protein		1.80	3.50E-04	0.003644311
Pden_0605		thiamine diphosphokinase		1.92	9.76E-05	0.00201788
Pden_0608		extracellular solute-binding protein, family 5		-2.02	4.80E-04	0.004348292
Pden_0610		amidohydrolase		-1.42	8.42E-04	0.00608831
Pden_0628		hypothetical protein		1.10	0.001158962	0.007427627
Pden_0632	*smpB*	SsrA-binding protein		1.59	1.62E-04	0.002501662
Pden_0645	*def*	peptide deformylase		1.39	0.001141422	0.007371199
Pden_0646	*def*	Peptide deformylase		1.30	6.54E-04	0.005207841
Pden_0654		hypothetical protein		1.49	4.09E-04	0.003981134
Pden_0659	*topA*	DNA topoisomerase I		1.69	9.38E-05	0.001988942
Pden_0660	*ggt*	gamma-glutamyltransferase 1, Threonine peptidase, MEROPS family T03		-2.47	5.77E-05	0.001613164
Pden_0661		protein of unknown function DUF488		2.20	3.66E-04	0.003728529
Pden_0665		phosphate transporter		1.85	3.93E-04	0.003899314
Pden_0677		putative hydantoin racemase protein		-1.95	0.001103207	0.007188594
Pden_0691		chorismate mutase		1.50	3.01E-04	0.003398128
Pden_0706		protein of unknown function DUF6, transmembrane		1.58	3.89E-04	0.003876944
Pden_0707		inositol monophosphatase		1.70	1.59E-04	0.002464835
Pden_0738		transferase hexapeptide repeat containing protein		2.49	1.40E-04	0.002302411
Pden_0739		ABC transporter related protein		2.19	2.30E-04	0.002922473
Pden_0740		hypothetical protein		2.68	8.99E-05	0.001961867
Pden_0742	*nusG*	transcription antitermination protein nusG		2.17	3.48E-05	0.001297726
Pden_0744	*rplA*	LSU ribosomal protein L1P		2.05	2.04E-04	0.002760804
Pden_0745	*rplJ*	LSU ribosomal protein L10P		3.56	7.20E-05	0.001785877
Pden_0746	*rplL*	LSU ribosomal protein L12P / LSU ribosomal protein L12P		3.18	9.15E-05	0.001979539
Pden_0749	*rpoC*	DNA-directed RNA polymerase subunit beta'		1.50	0.001118009	0.007229223
Pden_0759	*rplC*	LSU ribosomal protein L3P		2.47	4.39E-04	0.004136616
Pden_0760	*rplD*	LSU ribosomal protein L4P		3.11	5.75E-05	0.001613164
Pden_0765	*rpsC*	SSU ribosomal protein S3P		2.49	1.92E-04	0.002717725
Pden_0766	*rplP*	LSU ribosomal protein L16P		2.31	0.001176391	0.007497765
Pden_0776	*rpsE*	SSU ribosomal protein S5P		2.06	0.001690065	0.009465483
Pden_0778		conserved hypothetical protein		2.69	1.17E-04	0.002162707
Pden_0779	*rplO*	LSU ribosomal protein L15P		2.84	7.28E-05	0.001797426
Pden_0784	*rpoA*	DNA-directed RNA polymerase subunit alpha		2.55	0.001003841	0.006795278
Pden_0791		HAD-superfamily hydrolase, subfamily IA, variant 3		1.30	0.001075262	0.007079394
Pden_0794		L-asparagine ABC transporter membrane protein / L-glutamine ABC transporter membrane protein / L-glutamate ABC transporter membrane protein / L-aspartate ABC transporter membrane protein		-1.80	0.00116584	0.007452837
Pden_0810		extracellular solute-binding protein, family 5		-1.81	8.91E-04	0.006310843
Pden_0812	*serB*	phosphoserine phosphatase		1.58	5.94E-04	0.004969545
Pden_0813		phosphoserine aminotransferase apoenzyme		1.49	4.71E-04	0.004312253
Pden_0823		protein of unknown function DUF404		-1.74	1.06E-04	0.002072833
Pden_0824		protein of unknown function DUF403		-1.37	3.98E-04	0.003908229
Pden_0827	*mutS*	DNA mismatch repair protein MutS		1.70	0.001510105	0.008849146
Pden_0829		extracellular solute-binding protein, family 1		-1.85	2.33E-04	0.002932574
Pden_0850	*trhO*	Rhodanese domain protein		1.63	1.60E-04	0.002483517
Pden_0856		Lysine exporter protein (LYSE/YGGA)		1.05	0.0013187	0.008092821
Pden_0863	*ptsP*	PTSINtr with GAF domain, PtsP		1.45	0.001739641	0.009626016
Pden_0864		ABC transporter related protein		2.98	6.97E-05	0.001761223
Pden_0865		transport system permease protein		2.45	0.001376701	0.008325092
Pden_0866		periplasmic binding protein		1.46	4.60E-04	0.004272675
Pden_0867	*tsaA*	protein of unknown function UPF0066		1.40	3.33E-04	0.003583012
Pden_0868		hypothetical protein		1.13	0.001117699	0.007229223
Pden_0872	*rpmH*	LSU ribosomal protein L34P		1.72	1.95E-04	0.002717725
Pden_0873	*rnpA*	ribonuclease P protein component		1.92	8.56E-05	0.001917348
Pden_0874	*yidD*	protein of unknown function DUF37		1.66	4.31E-04	0.004090524
Pden_0876		diguanylate phosphodiesterase		2.37	1.45E-04	0.00231305
Pden_0877	*yidC*	protein translocase subunit yidC		1.61	2.64E-04	0.003162675
Pden_0878		MOSC domain containing protein		2.48	4.23E-05	0.001441542
Pden_0879	*engB*	small GTP-binding protein		2.61	7.38E-05	0.001805298
Pden_0887	*tig*	trigger factor		1.85	6.67E-04	0.005277599
Pden_0888		hypothetical protein		1.51	0.001210903	0.007644393
Pden_0889	*rplI*	LSU ribosomal protein L9P		4.32	1.28E-04	0.002273372
Pden_0894	*prfA*	bacterial peptide chain release factor 1 (bRF-1)		1.38	0.001090788	0.00713522
Pden_0895	*prmC*	[protein release factor]-glutamine N5-methyltransferase		2.39	3.41E-04	0.00363388
Pden_0896		conserved hypothetical protein		2.05	5.90E-05	0.001623803
Pden_0916		transcriptional regulator, TraR/DksA family		2.22	9.82E-04	0.006721522
Pden_0924		OsmC family protein		1.50	1.92E-04	0.002717725
Pden_0936		transcriptional regulator, MarR family		1.18	0.001844245	0.009969029
Pden_0939	*aspA*	aspartate ammonia-lyase		-1.53	0.001147932	0.007373321
Pden_0958	*phaC*	poly(R)-hydroxyalkanoic acid synthase, class I		-1.50	4.30E-04	0.004090524
Pden_0961		phage integrase family protein	2	2.04	1.84E-04	0.002652467
Pden_0962		conserved hypothetical protein	2	4.21	1.95E-05	9.86E-04
Pden_0963		conserved hypothetical protein	2	5.99	3.28E-07	9.06E-05
Pden_0964		hypothetical protein	2	5.63	1.61E-05	8.87E-04
Pden_0965		hypothetical protein	2	6.03	2.67E-05	0.001146189
Pden_0966		hypothetical protein	2	6.04	9.43E-07	1.45E-04
Pden_0967		hypothetical protein	2	6.12	1.84E-07	7.94E-05
Pden_0968		bacteriophage hypothetical protein	2	5.84	3.44E-04	0.003644311

Pden_0969		DNA methylase N-4/N-6 domain protein	2	6.85	1.62E-06	2.00E-04
Pden_0970		DNA polymerase III beta subunit family protein	2	6.34	7.65E-07	1.45E-04
Pden_0971		hypothetical protein	2	6.15	2.20E-07	7.94E-05
Pden_0972		hypothetical protein	2	6.18	7.63E-08	7.94E-05
Pden_0973		hypothetical protein	2	-2.60	0.001073263	0.007075428
Pden_0974		hypothetical protein	2	5.34	1.95E-04	0.002717725
Pden_0977		hypothetical protein	2	5.16	4.34E-07	1.10E-04
Pden_0979		hypothetical protein	2	6.72	2.60E-07	8.78E-05
Pden_0980		hypothetical protein	2	6.56	5.70E-07	1.15E-04
Pden_0981		hypothetical protein	2	6.31	5.16E-06	4.50E-04
Pden_0982		hypothetical protein	2	7.66	3.40E-07	9.06E-05
Pden_0983		phage/plasmid primase, P4 family	2	7.10	9.49E-08	7.94E-05
Pden_0984		hypothetical protein	2	6.57	3.66E-06	3.56E-04
Pden_0985		HNH endonuclease	2	6.28	8.75E-05	0.001937938
Pden_0986		hypothetical protein	2	6.95	1.11E-05	7.46E-04
Pden_0987		Resolvase helix-turn-helix domain protein	2	7.91	3.11E-07	9.06E-05
Pden_0988		phage Terminase	2	7.91	1.99E-07	7.94E-05
Pden_0989		phage portal protein, HK97 family	2	7.84	1.86E-07	7.94E-05
Pden_0990		peptidase S14, ClpP	2	7.45	1.82E-07	7.94E-05
Pden_0991		phage major capsid protein, HK97 family	2	7.60	2.09E-07	7.94E-05
Pden_0992		hypothetical protein	2	7.21	3.65E-05	0.001320801
Pden_0993		hypothetical protein	2	7.23	9.45E-07	1.45E-04
Pden_0994		hypothetical protein	2	7.63	2.10E-07	7.94E-05
Pden_0995		hypothetical protein	2	7.13	3.03E-05	0.001216131
Pden_0996		gene transfer agent	2	7.28	8.30E-06	6.18E-04
Pden_0997		phage protein, HK97 gp10 family	2	7.79	1.48E-05	8.86E-04
Pden_0998		hypothetical protein	2	7.62	5.36E-07	1.15E-04
Pden_0999		hypothetical protein	2	7.50	1.50E-07	7.94E-05
Pden_1000		phage tape measure protein	2	6.57	1.80E-06	2.17E-04
Pden_1001		hypothetical protein		5.83	1.96E-04	0.002717725
Pden_1002		hypothetical protein		5.80	3.78E-05	0.001337417
Pden_1003		conserved hypothetical protein		6.64	4.56E-07	1.10E-04
Pden_1004		hypothetical protein		7.43	2.06E-07	7.94E-05
Pden_1009		conserved hypothetical protein 698		1.91	0.001697942	0.009488612
Pden_1038		protein of unknown function DUF6, transmembrane		2.21	4.62E-05	0.00146383
Pden_1041	*nadX*	aspartate dehydrogenase		2.11	1.89E-04	0.002696005
Pden_1042		alpha/beta hydrolase fold protein		1.79	3.20E-04	0.003546894
Pden_1051		Aldehyde dehydrogenase (NAD(+))		1.95	4.61E-04	0.004272675
Pden_1056		hypothetical protein		1.58	5.12E-04	0.004509969
Pden_1057		N-isopropylammelide isopropylaminohydrolase		-2.59	4.13E-04	0.004008472
Pden_1058		transcriptional regulator, LysR family		1.44	8.03E-04	0.005892484
Pden_1077		carbohydrate ABC transporter ATP-binding protein, CUT1 family		3.10	5.58E-05	0.001611959
Pden_1079		binding-protein-dependent transport systems inner membrane component		3.19	1.37E-04	0.002292537
Pden_1080		beta-lactamase domain protein		2.82	1.94E-04	0.002717725
Pden_1109		rhodanese-like protein		-1.63	1.22E-04	0.002225558
Pden_1111		NCS1 nucleoside transporter family		-3.56	0.001700764	0.009493899
Pden_1112	*hydA*	dihydropyrimidinase		-3.99	0.001562951	0.009040019
Pden_1119	*npdG*	reduced coenzyme F420:NADP oxidoreductase		-4.00	2.69E-04	0.003197585
Pden_1120		methylenetetrahydromethanopterin reductase		-3.98	0.001406503	0.008437355
Pden_1132		PRC-barrel containing protein		2.99	1.30E-04	0.002284717
Pden_1134		short-chain dehydrogenase/reductase SDR		1.09	0.001660125	0.009345864
Pden_1136		protein of unknown function DUF892		3.70	3.28E-06	3.32E-04
Pden_1142		ABC transporter related protein		1.89	0.001360127	0.008247094
Pden_1144		conserved hypothetical protein		-2.23	0.001776894	0.009725855
Pden_1145		intracellular protease, PfpI family		-2.28	4.18E-04	0.004027362
Pden_1147		amino acid ABC transporter substrate-binding protein, PAAT family		-4.17	2.32E-04	0.002927786
Pden_1148		amino acid ABC transporter membrane protein 1, PAAT family		-3.54	5.65E-05	0.001613164
Pden_1149		amino acid ABC transporter membrane protein 2, PAAT family		-3.19	5.76E-04	0.004898771
Pden_1150		amino acid ABC transporter ATP-binding protein, PAAT family		-2.72	1.33E-04	0.002284717
Pden_1151		agmatinase		-1.64	4.19E-04	0.0040289
Pden_1163		NUDIX hydrolase		2.46	6.75E-04	0.005311929
Pden_1167		molybdenum ABC transporter, periplasmic molybdate-binding protein		2.46	2.42E-05	0.001105881
Pden_1172		TOBE domain protein		-1.63	2.16E-04	0.002830827
Pden_1175	*catC*	muconolactone delta-isomerase		-3.07	3.95E-04	0.003907767
Pden_1176	*catA*	catechol 1,2-dioxygenase		-4.31	0.00127546	0.007904108
Pden_1186	*fdhA*	glutathione-independent formaldehyde dehydrogenase		-2.85	1.03E-04	0.002055734
Pden_1199		amidohydrolase		-3.95	2.85E-05	0.001181612
Pden_1200		conserved hypothetical protein		-3.40	2.72E-05	0.001152121
Pden_1201		conserved hypothetical protein		-2.70	1.17E-04	0.002162707
Pden_1221		transposase, IS4 family		2.00	1.78E-04	0.002605071
Pden_1222		transposase-like protein		2.08	2.59E-04	0.00312771
Pden_1225		hypothetical protein		-1.42	0.001475236	0.008715426
Pden_1232		HI0933 family protein		1.52	4.79E-04	0.004348292
Pden_1255		short-chain dehydrogenase/reductase SDR		-2.67	2.65E-04	0.003174839
Pden_1275	*tkt*	transketolase		1.59	2.51E-04	0.003045163
Pden_1277		uracil-xanthine permease		-2.41	1.46E-04	0.00231305
Pden_1298	*gmd*	GDP-mannose 4,6-dehydratase		2.10	3.18E-05	0.001249299
Pden_1305		Antibiotic biosynthesis monooxygenase		-2.38	1.45E-04	0.00231305
Pden_1306		Cobyrinic acid a,c-diamide synthase		-1.25	7.05E-04	0.005423503
Pden_1314		periplasmic glucan biosynthesis protein, MdoG		1.14	0.001784956	0.009748901
Pden_1315	*mdoH*	glycosyl transferase, family 2		1.51	3.51E-04	0.003644311

Pden_1316		conserved hypothetical protein	2.15	2.91E-04	0.003349127
Pden_1323		pyridoxamine 5'-phosphate oxidase-related, FMN-binding protein	-1.14	9.91E-04	0.006753469
Pden_1327	*oppB*	binding-protein-dependent transport systems inner membrane component	-1.41	7.72E-04	0.005770981
Pden_1338		cobalamin synthesis protein, P47K	1.35	0.001037432	0.006920316
Pden_1341		extracellular solute-binding protein, family 5	2.11	1.65E-04	0.002535748
Pden_1342		binding-protein-dependent transport systems inner membrane component	2.66	4.13E-04	0.004008472
Pden_1346	*prpB*	2,3-dimethylmalate lyase	2.03	0.001723333	0.009573963
Pden_1348		2-methylcitrate dehydratase	2.62	1.37E-04	0.002292537
Pden_1349		thioesterase superfamily protein	3.47	6.82E-05	0.001743521
Pden_1351		LysR, substrate-binding protein	3.18	0.001560994	0.009040019
Pden_1374		metallophosphoesterase	1.76	2.15E-04	0.002823334
Pden_1377		Redoxin domain protein	3.44	1.24E-04	0.002256524
Pden_1420	*dusA*	tRNA-U16,U17-dihydrouridine synthase	1.76	3.52E-04	0.003644311
Pden_1431		transcriptional regulator, GntR family	-1.43	0.001356109	0.008232592
Pden_1432		BioY protein	-1.63	0.001205562	0.007629702
Pden_1433	*bioB*	biotin synthase	-2.53	2.02E-05	9.98E-04
Pden_1466		helix-turn-helix- domain containing protein, AraC type	-1.42	7.49E-04	0.005671257
Pden_1492		ABC transporter related protein	-1.40	4.61E-04	0.004272675
Pden_1497		transcriptional regulator, AraC family	-3.90	9.33E-05	0.001988942
Pden_1510		conjugal transfer protein precursor	2.27	0.001517436	0.008881825
Pden_1530		hypothetical protein	-1.61	0.001441672	0.008597392
Pden_1544		domain of unknown function DUF1738	2.95	4.86E-04	0.004381203
Pden_1545		histone family protein nucleoid-structuring protein H-NS	-1.23	0.001540036	0.008962304
Pden_1551		protein of unknown function DUF481	-1.94	0.001567675	0.009040019
Pden_1563		TRAP dicarboxylate transporter- DctP subunit	-3.46	7.78E-05	0.001836392
Pden_1570	*oppB*	binding-protein-dependent transport systems inner membrane component	-1.41	7.71E-04	0.005770981
Pden_1574		pyridoxamine 5'-phosphate oxidase-related, FMN-binding protein	-1.14	9.81E-04	0.006721522
Pden_1581		conserved hypothetical protein	2.15	2.90E-04	0.003349127
Pden_1582	*mdoH*	glycosyl transferase, family 2	1.52	3.31E-04	0.003583012
Pden_1583		periplasmic glucan biosynthesis protein, MdoG	1.14	0.001784207	0.009748901
Pden_1590		plasmid segregation oscillating ATPase ParF	-1.25	7.04E-04	0.005422813
Pden_1591		Antibiotic biosynthesis monooxygenase	-2.38	1.45E-04	0.00231305
Pden_1595		ABC transporter related protein	1.91	7.56E-04	0.005693964
Pden_1605		flavoprotein	-1.40	0.001775922	0.009725855
Pden_1641		conserved hypothetical protein 698	1.92	0.0017226	0.009573963
Pden_1647		TRAP dicarboxylate transporter, DctP subunit	-1.52	6.23E-04	0.005101564
Pden_1662		Hemolysin-type calcium-binding region	-2.07	7.09E-04	0.005447488
Pden_1670		binding-protein-dependent transport systems inner membrane component	-1.84	2.92E-04	0.003349127
Pden_1671		extracellular solute-binding protein, family 5	-4.11	0.00105622	0.006990384
Pden_1672	*iolB*	5-deoxyglucuronate isomerase	-1.36	8.55E-04	0.006160708
Pden_1674	*iolE*	2-keto-myo-inositol dehydratase	-1.81	0.001572919	0.009049647
Pden_1678	*iolG*	myo-inositol 2-dehydrogenase	-1.95	7.23E-04	0.005522182
Pden_1680		oxidoreductase domain protein	-2.31	2.74E-04	0.003242033
Pden_1681		monosaccharide ABC transporter substrate-binding protein, CUT2 family	-3.22	6.32E-04	0.005126214
Pden_1682		monosaccharide ABC transporter membrane protein, CUT2 family	-3.05	6.58E-05	0.001699507
Pden_1683	*rbsA*	monosaccharide ABC transporter ATP-binding protein, CUT2 family	-3.94	1.27E-04	0.0022686
Pden_1684		monosaccharide ABC transporter substrate-binding protein, CUT2 family	-4.00	3.18E-05	0.001249299
Pden_1692		creatinase	-1.77	0.001395237	0.008389647
Pden_1693		major facilitator superfamily MFS_1	-3.05	3.27E-04	0.003573445
Pden_1695	*fbp*	D-fructose 1,6-bisphosphatase	-2.98	9.20E-04	0.006484226
Pden_1697	*tkt*	transketolase	-2.62	1.69E-04	0.002556934
Pden_1698		fructose-bisphosphate aldolase	-3.16	8.44E-04	0.006098745
Pden_1699	*cbbL*	ribulose-1,5-bisphosphate carboxylase/oxygenase large subunit	-2.80	7.41E-04	0.005632016
Pden_1701	*cbbX*	AAA ATPase, central domain protein	-1.49	0.001117141	0.007229223
Pden_1703	*peaA*	quinohemoprotein amine dehydrogenase, 60 kDa subunit	-4.01	0.001817538	0.009884204
Pden_1704	*peaB*	Radical SAM domain protein	-4.52	0.001107394	0.007205428
Pden_1705	*qhpC*	conserved hypothetical protein	-4.82	0.001225191	0.007696203
Pden_1706	*peaD*	quinohemoprotein amine dehydrogenase, subunit beta	-4.67	0.001352545	0.008232592
Pden_1707		conserved hypothetical protein	-4.45	0.00145228	0.008637796
Pden_1709		transcriptional regulator, AraC family	-2.20	0.001198387	0.007593785
Pden_1710		Betaine-aldehyde dehydrogenase	-6.78	0.001726189	0.009573963
Pden_1712		glucose-methanol-choline oxidoreductase	-2.49	8.82E-05	0.001940458
Pden_1713		amino acid/amide ABC transporter membrane protein 2, HAAT family	-3.13	2.06E-05	9.98E-04
Pden_1714		amino acid/amide ABC transporter membrane protein 1, HAAT family	-3.40	4.48E-05	0.001444301
Pden_1715		amino acid/amide ABC transporter ATP-binding protein 2, HAAT family	-3.30	5.60E-05	0.001611959
Pden_1716		amino acid/amide ABC transporter ATP-binding protein 1, HAAT family	-3.90	1.07E-05	7.37E-04
Pden_1717		amino acid/amide ABC transporter substrate-binding protein, HAAT family	-4.29	6.97E-06	5.52E-04
Pden_1719		AMP-dependent synthetase and ligase	-1.90	4.64E-05	0.00146383
Pden_1721		transporter, UIT1 family	-3.23	2.12E-05	0.001002638
Pden_1735	*efeB*	Dyp-type peroxidase family	-1.26	5.98E-04	0.004987227
Pden_1736	*efeO*	protein of unknown function DUF451	-2.37	1.33E-04	0.002284717
Pden_1742	*dtd*	D-tyrosyl-tRNA(Tyr) deacylase	1.81	0.001143421	0.007373321
Pden_1747		ABC transporter related protein	1.94	7.79E-04	0.00579407
Pden_1761		protein of unknown function DUF6, transmembrane	1.14	0.001684206	0.009443117
Pden_1783		signal transduction histidine kinase, glucose-6-phosphate specific	-1.95	7.67E-04	0.005759382
Pden_1784		two component transcriptional regulator, LuxR family	-1.96	2.01E-04	0.002747242
Pden_1792		ribonuclease	1.33	3.25E-04	0.003563965
Pden_1793	*yacG*	protein of unknown function DUF329	1.81	2.09E-04	0.00277756
Pden_1795		Allergen V5/Tpx-1 family protein	-1.32	0.001315497	0.008082965
Pden_1814		Patatin	2.24	0.001272614	0.007904108
Pden_1815	*prfB*	bacterial peptide chain release factor 2 (bRF-2)	1.39	3.32E-04	0.003583012

Pden_1839	*lolD*	ABC transporter related protein	2.06	6.76E-04	0.005314314
Pden_1860	*plsX*	phosphate:acyl-[acyl carrier protein] acyltransferase	1.78	5.43E-04	0.004701006
Pden_1861	*fabH*	3-oxoacyl-[acyl-carrier-protein] synthase III	2.65	1.04E-04	0.002056763
Pden_1863	*ihfA*	integration host factor, alpha subunit	-1.15	0.001548992	0.008983446
Pden_1879	*carB*	carbamoyl-phosphate synthase large subunit	2.31	4.83E-05	0.0014723
Pden_1899	*choX*	Substrate-binding region of ABC-type glycine betaine transport system	-1.49	0.001566845	0.009040019
Pden_1901	*choV*	ABC transporter related protein	-1.59	5.85E-04	0.004954272
Pden_1922		hypothetical protein	1.41	3.94E-04	0.003899314
Pden_1923		ATP phosphoribosyltransferase catalytic subunit	2.31	6.19E-04	0.005085057
Pden_1924		ATP phosphoribosyltransferase regulatory subunit	1.91	1.96E-04	0.002717725
Pden_1925	*hisS*	histidyl-tRNA synthetase	1.33	0.001579552	0.009052351
Pden_1926		conserved hypothetical protein	-1.69	0.001466317	0.008690206
Pden_1929	*gatB*	aspartyl/glutamyl-tRNA(Asn/Gln) amidotransferase subunit B	1.37	0.001254743	0.007826119
Pden_1933		O-acetylhomoserine sulfhydrolase	-1.72	6.08E-04	0.005040533
Pden_1954		SAM-dependent methyltransferase	1.97	4.83E-04	0.004368669
Pden_1956		putative PAS/PAC sensor protein	1.73	3.63E-04	0.003710722
Pden_1962		hypothetical protein	5.68	0.001218577	0.007673704
Pden_1975		hypothetical protein	2.67	1.44E-04	0.00231305
Pden_1985		pterin-4-alpha-carbinolamine dehydratase	-1.44	0.001582363	0.009052351
Pden_2005		AMP-dependent synthetase and ligase	-1.90	1.71E-04	0.002579539
Pden_2026		acetyl-CoA acetyltransferase	-1.70	8.08E-04	0.005905824
Pden_2034		toluene tolerance family protein	-1.18	0.001149032	0.007373321
Pden_2040		GCN5-related N-acetyltransferase	1.89	3.53E-04	0.003644311
Pden_2041	*argD*	acetylornithine aminotransferase apoenzyme	1.93	6.73E-05	0.001728981
Pden_2046		TonB-dependent receptor	-1.74	1.77E-04	0.002605071
Pden_2047	*rocF*	arginase	-1.60	3.50E-04	0.003644311
Pden_2058		conserved hypothetical protein	1.85	1.27E-04	0.0022686
Pden_2064		phage terminase, small subunit, putative, P27 family	2.16	6.16E-04	0.005082021
Pden_2068		ParB domain protein nuclease	2.52	7.16E-05	0.00178563
Pden_2072		hypothetical protein	2.82	2.12E-04	0.002800179
Pden_2078		hypothetical protein	-2.21	0.001334795	0.008161915
Pden_2082		conserved hypothetical protein	2.34	0.001493028	0.008800002
Pden_2093		3-deoxy-D-manno-octulosonic-acid transferase-like protein	1.90	6.52E-04	0.005207841
Pden_2101		DNA methylase N-4/N-6 domain protein	-1.52	9.95E-04	0.006767899
Pden_2119	*rfbA*	Glucose-1-phosphate thymidylyltransferase	1.67	0.001230011	0.007708806
Pden_2120	*rfbD*	dTDP-4-dehydrorhamnose reductase	1.95	3.80E-04	0.00382151
Pden_2121	*rfbB*	dTDP-glucose 4,6-dehydratase	1.08	0.001066507	0.007049252
Pden_2125		ABC-2 type transporter	1.74	0.001310916	0.008074414
Pden_2127		ABC transporter related protein	1.75	9.73E-04	0.00671291
Pden_2131		GCN5-related N-acetyltransferase	1.85	8.36E-04	0.006066726
Pden_2142	*lexA*	SOS-response transcriptional repressor, LexA	1.81	5.04E-04	0.004472377
Pden_2147		beta-lactamase domain protein	1.11	0.001023976	0.006875847
Pden_2164		GatB/Yqey domain protein	1.54	2.31E-04	0.002927163
Pden_2165	*carA*	carbamoyl-phosphate synthase small subunit	1.63	3.36E-04	0.003607393
Pden_2166		General secretory system II, protein E domain protein	2.57	1.21E-04	0.002215935
Pden_2172		peptidase M48, Ste24p	-1.21	0.001149015	0.007373321
Pden_2183	*aspS*	aspartyl-tRNA synthetase	1.66	4.99E-04	0.0044545
Pden_2204		conserved hypothetical protein	-1.16	8.63E-04	0.006186303
Pden_2225		propionyl-CoA synthetase	-2.37	2.23E-04	0.002861957
Pden_2226	*aroA*	3-phosphoshikimate 1-carboxyvinyltransferase	2.03	2.04E-04	0.002760804
Pden_2251		conserved hypothetical protein	2.20	2.49E-04	0.003045163
Pden_2252	*uvrA*	Excinuclease ABC subunit A	1.76	8.43E-05	0.0019055
Pden_2255		conserved hypothetical protein	-1.27	6.35E-04	0.005138409
Pden_2257		succinate semialdehyde dehydrogenase	-1.88	2.29E-04	0.002907856
Pden_2262	*pheT*	phenylalanyl-tRNA synthetase beta subunit	1.88	1.49E-04	0.002343273
Pden_2272		cytochrome P450	2.13	3.30E-04	0.003583012
Pden_2275		hypothetical protein	2.24	6.57E-05	0.001699507
Pden_2276	*pyk*	pyruvate kinase	1.82	1.35E-04	0.002284871
Pden_2285	*choX*	Substrate-binding region of ABC-type glycine betaine transport system	-2.24	8.52E-04	0.006144644
Pden_2287		membrane protein of uknown function UCP014873	1.67	0.001600246	0.009093208
Pden_2292		conserved hypothetical protein	1.79	0.001029438	0.006894242
Pden_2293		glycosyl transferase, family 14	2.27	5.29E-05	0.001560115
Pden_2294		conserved hypothetical protein	1.83	1.72E-04	0.002586479
Pden_2297		UDP-glucose pyrophosphorylase	-1.25	0.001455272	0.008637796
Pden_2327		protein of unknown function DUF1428	-2.48	0.001052695	0.006987997
Pden_2328		Glyoxalase/bleomycin resistance protein/dioxygenase	-1.93	1.33E-04	0.002284871
Pden_2336	*mnmD*	protein of unknown function DUF752	1.83	7.92E-04	0.005842821
Pden_2338		4-aminobutyrate aminotransferase apoenzyme	-1.94	8.68E-04	0.006205608
Pden_2351		conserved hypothetical protein	-1.46	4.71E-04	0.004312253
Pden_2354		two component transcriptional regulator, LuxR family	-3.35	2.06E-04	0.002764361
Pden_2359	*pqqE*	coenzyme PQQ biosynthesis protein E	-2.06	9.61E-04	0.006680258
Pden_2360	*pqqD*	coenzyme PQQ synthesis D	-2.60	7.14E-05	0.00178563
Pden_2361	*pqqC*	coenzyme PQQ biosynthesis protein C	-3.02	2.63E-05	0.001142137
Pden_2362	*pqqB*	coenzyme PQQ biosynthesis protein B	-2.93	1.17E-04	0.002162707
Pden_2366	*adh*	Aldehyde dehydrogenase (NAD(+))	-4.07	0.001661323	0.009345864
Pden_2367	*adhP*	Alcohol dehydrogenase GroES domain protein	-3.17	2.92E-04	0.003349127
Pden_2368		protein of unknown function DUF779	-3.33	0.001584117	0.009052351
Pden_2380		metallophosphoesterase	1.47	4.02E-04	0.003919705
Pden_2386		2,3-dihydroxybenzoate-AMP ligase	-1.48	0.001274921	0.007904108
Pden_2393	*gndA*	6-phosphogluconate dehydrogenase (decarboxylating)	2.34	0.001381129	0.008334513
Pden_2394		hypothetical protein	3.63	4.29E-05	0.001441542

Pden_2395		hypothetical protein	1.30	0.001213854	0.00765348
Pden_2406	*ddl*	D-alanine--D-alanine ligase	-1.82	0.001087746	0.007124523
Pden_2414		hypothetical protein	-2.56	6.42E-04	0.005154003
Pden_2428		hypothetical protein	2.53	3.92E-04	0.003894981
Pden_2443	*tssB*	Uncharacterized conserved protein UCP028301	-2.57	2.82E-04	0.003317154
Pden_2444	*tssC*	protein of unknown function DUF877	-2.70	3.62E-04	0.003710685
Pden_2459		OmpA/MotB domain protein	-2.32	0.001189961	0.007559311
Pden_2468		cation diffusion facilitator family transporter	2.72	3.77E-04	0.003809005
Pden_2469		carbohydrate ABC transporter ATP-binding protein, CUT1 family	-1.64	3.31E-04	0.003583012
Pden_2470		carbohydrate ABC transporter membrane protein 2, CUT1 family	-2.46	7.75E-04	0.005785047
Pden_2471		carbohydrate ABC transporter membrane protein 1, CUT1 family	-2.39	2.89E-04	0.003349127
Pden_2472		carbohydrate ABC transporter substrate-binding protein, CUT1 family	-2.50	5.25E-05	0.001560115
Pden_2483		nitric oxide reductase, NorB subunit apoprotein	-1.93	6.01E-05	0.001626459
Pden_2486	*nirI*	FMN-binding domain protein	3.07	0.00102084	0.006863897
Pden_2502		protein of unknown function DUF81	2.75	1.77E-04	0.002605071
Pden_2506	*gpmI*	phosphoglycerate mutase	1.46	4.98E-04	0.0044545
Pden_2547		short-chain dehydrogenase/reductase SDR	2.54	1.45E-04	0.00231305
Pden_2565		putative esterase	-1.41	0.001748119	0.009652871
Pden_2579		Glutathione S-transferase, N-terminal domain	2.23	0.0012438	0.007774516
Pden_2591	*rimP*	protein of unknown function DUF150	1.93	5.93E-05	0.001623926
Pden_2592	*nusA*	NusA antitermination factor	1.42	3.64E-04	0.003718072
Pden_2593		protein of unknown function DUF448	1.66	7.93E-04	0.005842821
Pden_2594	*infB*	bacterial translation initiation factor 2 (bIF-2)	1.71	2.25E-04	0.00286665
Pden_2629		hypothetical protein	3.24	0.001502047	0.008822351
Pden_2649	*fliF*	flagellar M-ring protein FliF	1.55	0.001306885	0.008059387
Pden_2663		acetyl-CoA acetyltransferase	-2.34	2.09E-05	9.98E-04
Pden_2664		putative transcriptional regulator, ModE family	-1.94	3.10E-04	0.003479584
Pden_2680		glucokinase	3.01	2.75E-05	0.001152121
Pden_2681		hypothetical protein	2.27	3.51E-04	0.003644311
Pden_2682	*ahcY*	adenosylhomocysteinase	1.11	0.001827499	0.009906452
Pden_2703		transposase, IS4 family	2.00	1.49E-04	0.002343273
Pden_2704		transposase-like protein	2.08	1.35E-04	0.002284871
Pden_2711		conserved hypothetical protein	-1.21	0.001753833	0.009672829
Pden_2718	*purH*	IMP cyclohydrolase	1.72	5.68E-04	0.0048638
Pden_2732		protein of unknown function DUF81	-1.19	0.001094611	0.007146149
Pden_2741		hypothetical protein	2.88	0.001683368	0.009443117
Pden_2743	*efp*	translation elongation factor P (EF-P)	2.92	1.31E-04	0.002284717
Pden_2759	*pnp*	3' exoribonuclease	2.76	7.90E-05	0.001836392
Pden_2775	*asd*	aspartate semialdehyde dehydrogenase	1.80	3.49E-04	0.003644311
Pden_2803		beta-lactamase domain protein	-3.10	2.60E-04	0.003136054
Pden_2830		formate dehydrogenase (quinone-dependent) catalytic subunit	-2.47	2.90E-04	0.003349127
Pden_2831		ferredoxin	-2.70	6.96E-05	0.001761223
Pden_2832		Rieske (2Fe-2S) domain protein	-2.83	1.32E-04	0.002284717
Pden_2852	*pckA*	Phosphoenolpyruvate carboxykinase (ATP)	-3.58	1.54E-04	0.002418481
Pden_2856	*fdhF*	formate dehydrogenase alpha subunit	-2.21	4.73E-05	0.001471351
Pden_2871		3-hydroxyacyl-CoA dehydrogenase	-1.91	9.65E-04	0.006681372
Pden_2876	*atpF*	H+-transporting two-sector ATPase, B/B' subunit	2.23	6.38E-04	0.005142188
Pden_2877	*atpF*	H+-transporting two-sector ATPase, B/B' subunit	1.51	0.001587755	0.009052708
Pden_2878	*atpE*	ATP synthase F0 subcomplex C subunit	1.89	1.31E-04	0.002284717
Pden_2906		acyl-CoA dehydrogenase domain protein	-1.84	0.001377924	0.008325092
Pden_2908		short-chain dehydrogenase/reductase SDR	-2.12	6.90E-04	0.005364018
Pden_2925		iron-sulfur cluster binding protein	-2.20	0.001438767	0.00859365
Pden_2926		protein of unknown function DUF224, cysteine-rich region domain protein	-3.84	0.00145438	0.008637796
Pden_2927		L-lactate transport	-3.87	3.79E-04	0.003819736
Pden_2941		efflux transporter, RND family, MFP subunit	2.87	4.22E-04	0.004038296
Pden_2942		transporter, hydrophobe/amphiphile efflux-1 (HAE1) family	2.65	9.68E-05	0.002008388
Pden_2943		RND efflux system, outer membrane lipoprotein, NodT family	2.98	1.10E-04	0.002126235
Pden_2948		antifreeze protein, type I	-1.69	0.001114057	0.007229223
Pden_2970	*rpsI*	SSU ribosomal protein S9P	3.03	1.39E-04	0.002302411
Pden_2985		transcriptional regulator, XRE family	2.43	9.35E-04	0.006564078
Pden_2990		two component transcriptional regulator, LuxR family	-2.67	1.78E-04	0.002605071
Pden_3032		ABC transporter related protein	1.47	5.76E-04	0.004898771
Pden_3036		sulphate transporter	2.45	6.47E-05	0.001696353
Pden_3049		Transketolase, central region	-1.35	6.25E-04	0.005101822
Pden_3067		conserved hypothetical protein	1.88	6.71E-04	0.005289506
Pden_3071		aconitase	-1.67	4.18E-04	0.004027362
Pden_3073		protein of unknown function DUF112, transmembrane	-3.79	2.42E-05	0.001105881
Pden_3074		putative tricarboxylic transport membrane protein	-5.47	9.46E-04	0.006621591
Pden_3075		Uncharacterized protein UPF0065	-5.41	3.43E-05	0.001296365
Pden_3077		gene transfer agent (GTA) like protein	2.20	0.00152342	0.008897814
Pden_3098		nickel-dependent hydrogenase, large subunit	-3.00	0.001083457	0.007105623
Pden_3099		HupE/UreJ protein	-2.18	1.02E-04	0.002055734
Pden_3108	*hypB*	hydrogenase accessory protein HypB	-1.78	2.19E-04	0.002830827
Pden_3109		two component, sigma54 specific, transcriptional regulator, Fis family	-1.41	7.79E-04	0.00579407
Pden_3111	*hypD*	hydrogenase expression/formation protein HypD	-1.62	2.97E-04	0.003369347
Pden_3113		hypothetical protein	1.43	6.95E-04	0.005377582
Pden_3148		protease Do	-1.23	7.83E-04	0.005805533
Pden_3149		Phytanoyl-CoA dioxygenase	3.07	7.56E-04	0.005693964
Pden_3169		hypothetical protein	1.74	2.20E-04	0.002838625
Pden_3170		hypothetical protein	1.92	6.93E-04	0.005373648
Pden_3181		conserved hypothetical protein	3.04	4.35E-05	0.001441542

Pden_3182		C-5 cytosine-specific DNA methylase	1.88	6.27E-04	0.005114959
Pden_3211		aldehyde dehydrogenase	-1.30	0.001054816	0.006990226
Pden_3219	*dctP*	TRAP dicarboxylate transporter- DctP subunit	-2.00	6.89E-04	0.005364018
Pden_3221		Uncharacterized protein UPF0065	-2.07	0.001439347	0.00859365
Pden_3233		transposase IS116/IS110/IS902 family protein	-1.74	3.09E-04	0.003477827
Pden_3235		conserved hypothetical protein	-2.55	0.001771505	0.009725855
Pden_3260		aldehyde dehydrogenase	-1.86	1.01E-04	0.00205033
Pden_3266		Uncharacterized protein UPF0065	-2.94	9.05E-05	0.001966924
Pden_3267		amidohydrolase 2	-2.16	5.80E-05	0.001613164
Pden_3268		protein of unknown function DUF112, transmembrane	-2.09	3.21E-04	0.003546894
Pden_3271		transcriptional regulator, LysR family	-2.56	1.85E-05	9.57E-04
Pden_3286		cytochrome B561	-1.17	0.001823154	0.009896267
Pden_3294		Heavy metal transport/detoxification protein	-1.37	5.92E-04	0.004969545
Pden_3295		heavy metal translocating P-type ATPase	-1.99	1.11E-04	0.00212942
Pden_3302		transcriptional regulator, TetR family	2.78	0.001035658	0.006917597
Pden_3327		hypothetical protein	3.10	2.85E-04	0.003327115
Pden_3344		sulphate transporter	3.11	6.77E-06	5.44E-04
Pden_3345		protein of unknown function DUF1127	3.17	2.19E-04	0.002830827
Pden_3351		Methyltransferase type 11	1.80	1.82E-04	0.002639452
Pden_3357		DNA methylase N-4/N-6 domain protein	2.49	1.14E-04	0.002162073
Pden_3377		conserved hypothetical protein	1.66	0.001774624	0.009725855
Pden_3386		cyclase family protein	-1.97	3.35E-04	0.003605638
Pden_3387		TRAP dicarboxylate transporter, DctP subunit	-2.48	0.001027972	0.006893537
Pden_3400		molybdenum cofactor synthesis domain	3.41	1.08E-05	7.37E-04
Pden_3407	*ugpC*	carbohydrate ABC transporter ATP-binding protein, CUT1 family	1.63	8.39E-04	0.006076562
Pden_3411		hypothetical protein	2.13	2.38E-04	0.00295528
Pden_3424		dihydrodipicolinate synthetase	-1.19	0.001053099	0.006987997
Pden_3425		branched chain amino acid: 2-keto-4-methylthiobutyrate aminotransferase	-1.30	0.001325057	0.008112171
Pden_3428		Tripartite ATP-independent periplasmic transporter, DctQ component	-2.27	3.99E-04	0.003909759
Pden_3429		TRAP dicarboxylate transporter, DctM subunit	-2.37	5.52E-05	0.001611959
Pden_3430	*pxpB*	Allophanate hydrolase subunit 1	-1.71	9.46E-04	0.006621591
Pden_3447		efflux transporter, RND family, MFP subunit	-1.73	1.69E-04	0.002560706
Pden_3448		transporter, hydrophobe/amphiphile efflux-1 (HAE1) family	-1.68	2.46E-04	0.003014888
Pden_3450		beta-lactamase	-2.99	4.65E-05	0.00146383
Pden_3463		FAD-dependent pyridine nucleotide-disulfide oxidoreductase	1.28	8.28E-04	0.006025401
Pden_3464		ferredoxin	-1.49	0.001070733	0.007067956
Pden_3465		Rieske (2Fe-2S) domain protein	-2.34	5.80E-04	0.004918563
Pden_3480		TRAP dicarboxylate transporter, DctP subunit	-3.75	6.08E-04	0.005040533
Pden_3481		Tripartite ATP-independent periplasmic transporter, DctQ component	-2.58	2.93E-05	0.001194403
Pden_3482		TRAP dicarboxylate transporter, DctM subunit	-2.00	2.35E-04	0.002938416
Pden_3497		TRAP dicarboxylate transporter- DctP subunit	-2.31	8.38E-05	0.001901969
Pden_3513		transcriptional regulator, LysR family	1.37	9.81E-04	0.006721522
Pden_3521		TonB-dependent siderophore receptor	-2.31	5.19E-04	0.00455594
Pden_3537		short-chain dehydrogenase/reductase SDR	-3.71	7.87E-05	0.001836392
Pden_3538		TRAP-T family transporter, DctQ (4 TMs) subunit	-3.94	2.18E-04	0.002830827
Pden_3539		TRAP dicarboxylate transporter, DctM subunit	-3.86	8.98E-04	0.006350061
Pden_3540		TRAP dicarboxylate transporter- DctP subunit	-4.67	2.91E-04	0.003349127
Pden_3541		short-chain dehydrogenase/reductase SDR	-3.99	1.26E-04	0.00226507
Pden_3542		short-chain dehydrogenase/reductase SDR	-3.61	2.78E-04	0.003283054
Pden_3543		fumarate reductase/succinate dehydrogenase flavoprotein domain protein	-3.42	3.88E-04	0.003876944
Pden_3544		hypothetical protein	-2.80	6.12E-04	0.005066185
Pden_3550	*pseF*	acylneuraminate cytidylyltransferase	1.49	7.53E-04	0.005688481
Pden_3551		flagellin modification protein FlmD	1.36	7.95E-04	0.005849634
Pden_3557	*pseC*	DegT/DnrJ/EryC1/StrS aminotransferase	1.54	6.46E-04	0.005176075
Pden_3568		integral membrane sensor signal transduction histidine kinase	-2.07	3.53E-04	0.003644311
Pden_3574		amino acid/amide ABC transporter substrate-binding protein, HAAT family	-4.28	1.94E-04	0.002717725
Pden_3575		amino acid/amide ABC transporter membrane protein 1, HAAT family	-4.70	4.48E-04	0.004203157
Pden_3577		3-hydroxyacyl-CoA dehydrogenase, NAD-binding protein	-2.43	8.79E-04	0.006260085
Pden_3578		protein of unknown function DUF849	-2.70	0.001772721	0.009725855
Pden_3582		response regulator receiver protein	-1.92	0.001372627	0.008312932
Pden_3583		short-chain dehydrogenase/reductase SDR	-2.05	4.86E-04	0.004381203
Pden_3584		FAD-linked oxidoreductase	-1.97	2.67E-04	0.003191705
Pden_3585	*eutH*	Ethanolamine utilization protein, EutH	-2.45	5.12E-04	0.004509969
Pden_3586		transglutaminase domain protein	-1.47	4.99E-04	0.0044545
Pden_3601		Fibronectin, type III domain protein	1.67	6.87E-04	0.005364018
Pden_3604		hypothetical protein	1.48	9.67E-04	0.006681372
Pden_3608		hypothetical protein	4.03	1.52E-05	8.86E-04
Pden_3615		Glyoxalase/bleomycin resistance protein/dioxygenase	3.06	3.14E-04	0.003513638
Pden_3633		isovaleryl-CoA dehydrogenase	-1.52	0.00158145	0.009052351
Pden_3643		major facilitator superfamily MFS_1	4.92	6.29E-04	0.005116599
Pden_3652		ABC transporter related protein	1.64	8.67E-04	0.006205608
Pden_3662		FMN adenylyltransferase / riboflavin kinase	1.80	4.46E-04	0.004192833
Pden_3674		NUDIX hydrolase	2.08	8.77E-05	0.001937938
Pden_3675		conserved hypothetical protein	1.72	0.001003319	0.006795278
Pden_3678		homoserine dehydrogenase	1.49	5.69E-04	0.004864184
Pden_3695		GCN5-related N-acetyltransferase	-1.28	6.70E-04	0.005289506
Pden_3698	*trmJ*	RNA methyltransferase, TrmH family, group 1	2.72	1.15E-04	0.002162707
Pden_3701	*miaB*	tRNA-i(6)A37 thiotransferase enzyme MiaB	2.00	3.60E-04	0.00370427
Pden_3712	*secG*	protein translocase subunit secG	2.32	9.50E-05	0.001988942
Pden_3714		short-chain dehydrogenase/reductase SDR	1.64	0.00171384	0.009545843
Pden_3720		diguanylate cyclase	1.31	0.001467534	0.008690206

Pden_3725	*tsf*	translation elongation factor Ts (EF-Ts)		1.94	7.33E-04	0.005580508
Pden_3746		putative transcriptional regulator, XRE family	4	-1.58	0.001472743	0.008710862
Pden_3748		hypothetical protein	4	2.61	1.36E-04	0.002288892
Pden_3750		hypothetical protein	4	1.73	8.76E-04	0.006243388
Pden_3751		conserved hypothetical protein	4	2.21	0.001392111	0.008380808
Pden_3758		hypothetical protein	4	1.72	4.22E-04	0.004038296
Pden_3784	*cmk*	cytidylate kinase		2.47	8.90E-04	0.006310843
Pden_3785		2-nitropropane dioxygenase, NPD		2.38	5.49E-04	0.004745206
Pden_3796		Xanthine/uracil/vitamin C permease		-1.97	4.32E-04	0.004092226
Pden_3800		extracellular solute-binding protein, family 5		-2.08	9.41E-05	0.001988942
Pden_3801		binding-protein-dependent transport systems inner membrane component		-1.15	0.001597963	0.009090436
Pden_3802		binding-protein-dependent transport systems inner membrane component		-1.24	0.001763187	0.009703278
Pden_3811	*mfd*	transcription-repair coupling factor		1.49	4.64E-04	0.004286287
Pden_3812	*clpA*	ATP-dependent Clp protease, ATP-binding subunit clpA		-1.87	4.01E-04	0.003919705
Pden_3819	*atpC*	ATP synthase F1 subcomplex epsilon subunit		1.37	8.60E-04	0.006174412
Pden_3824		CoA-binding domain protein		-2.87	3.98E-04	0.003908229
Pden_3828		hypothetical protein		4.59	4.71E-04	0.004312253
Pden_3829		hypothetical protein		3.90	2.97E-06	3.07E-04
Pden_3834		hypothetical protein		3.13	4.49E-04	0.004204226
Pden_3849		phage integrase family protein		-2.89	0.001850915	0.0099906
Pden_3851		hypothetical protein		2.84	5.00E-04	0.0044545
Pden_3852		hypothetical protein		2.69	1.74E-04	0.002604995
Pden_3853		hypothetical protein		2.10	5.01E-04	0.004457229
Pden_3855		hypothetical protein		5.18	0.001527064	0.008907286
Pden_3856		Substrate-binding region of ABC-type glycine betaine transport system		1.34	7.67E-04	0.005759382
Pden_3857		binding-protein-dependent transport systems inner membrane component		1.99	6.97E-04	0.005387495
Pden_3860		NUDIX hydrolase		2.80	3.20E-04	0.003546894
Pden_3867		DedA family protein		2.32	0.001761605	0.009703278
Pden_3868		zinc metallopeptidase putative		-1.62	0.001298928	0.008020086
Pden_3871		conserved hypothetical protein		2.34	6.19E-04	0.005085057
Pden_3872	*gdhA*	glutamate dehydrogenase (NADP)		2.94	9.34E-04	0.006564078
Pden_3874		conserved hypothetical protein		1.82	2.91E-04	0.003349127
Pden_3887		chromosome partitioning protein		1.43	8.18E-04	0.005967453
Pden_3895		conserved hypothetical protein		-1.50	0.001355938	0.008232592
Pden_3916		amidohydrolase		-2.78	6.31E-05	0.001673359
Pden_3917		uracil-xanthine permease		-3.27	2.50E-04	0.003045163
Pden_3918	*bhcD*	ornithine cyclodeaminase		-1.70	8.23E-04	0.005993794
Pden_3926	*purM*	phosphoribosylformylglycinamidine cyclo-ligase		1.74	9.63E-04	0.006681372
Pden_3927	*purN*	formyltetrahydrofolate-dependent phosphoribosylglycinamide formyltransferase		1.49	2.38E-04	0.00295528
Pden_3932		amino acid/amide ABC transporter substrate-binding protein, HAAT family		-2.82	7.81E-05	0.001836392
Pden_3936	*gmk*	guanylate kinase		1.45	0.001005266	0.006795278
Pden_3951		Radical SAM domain protein		3.64	4.77E-05	0.00147144
Pden_3971	*purL*	phosphoribosylformylglycinamidine synthase subunit II		1.65	2.84E-04	0.003327115
Pden_3976		conserved hypothetical protein		2.14	4.14E-04	0.004011589
Pden_3988		VWA containing CoxE family protein		1.36	4.75E-04	0.004327427
Pden_3989		Ppx/GppA phosphatase		1.89	0.001823665	0.009896267
Pden_3997	*dxr*	1-deoxy-D-xylulose 5-phosphate reductoisomerase		1.58	8.69E-04	0.006205608
Pden_4002		Penicillin amidase		2.29	1.03E-04	0.002055734
Pden_4003		3-hydroxyisobutyrate dehydrogenase		-1.34	3.73E-04	0.003774183
Pden_4025	*typA*	GTP-binding protein TypA		1.84	1.41E-04	0.00230248
Pden_4027		ferredoxin		1.60	0.001353816	0.008232592
Pden_4029	*purD*	phosphoribosylamine--glycine ligase		2.05	7.02E-04	0.005417368
Pden_4032	*mreB*	rod shape-determining protein MreB		1.79	2.12E-04	0.002800179
Pden_4033	*mreC*	rod shape-determining protein MreC		1.98	1.00E-04	0.00205033
Pden_4049		protein of unknown function DUF1498		-1.69	3.61E-04	0.00370427
Pden_4051	*glcB*	Malate synthase		1.36	5.89E-04	0.004960806
Pden_4055	*proB*	glutamate 5-kinase		2.46	8.34E-04	0.006055957
Pden_4074		transcriptional regulator, TetR family		2.40	5.91E-04	0.004969545
Pden_4078	*trmFO*	gid protein		2.45	4.45E-05	0.001443847
Pden_4101		GCN5-related N-acetyltransferase		1.56	6.53E-04	0.005207841
Pden_4122		TRAP dicarboxylate transporter, DctP subunit		-3.97	0.001590823	0.009059996
Pden_4136		hypothetical protein		-1.79	7.18E-04	0.005491965
Pden_4140		periplasmic solute binding protein		1.57	0.001582917	0.009052351
Pden_4141		hypothetical protein		-2.61	8.08E-04	0.005905824
Pden_4143	*tadA*	CMP/dCMP deaminase, zinc-binding protein		1.55	4.91E-04	0.004414551
Pden_4149		thioredoxin SoxW		-1.33	7.77E-04	0.005793374
Pden_4150	*soxX*	monoheme cytochrome SoxX (sulfur oxidation)		-3.34	1.19E-05	7.68E-04
Pden_4151	*soxY*	thiosulfate-binding protein SoxY		-3.18	1.58E-04	0.002456618
Pden_4153	*soxA*	diheme cytochrome SoxA (sulfur oxidation)		-3.09	1.39E-04	0.002302411
Pden_4154	*soxB*	sulfate thiol esterase SoxB		-1.53	0.001586503	0.009052708
Pden_4167		ABC transporter related protein		1.76	9.78E-04	0.006721522
Pden_4169		putative sulfonate/nitrate transport system substrate-binding protein		2.39	4.73E-04	0.004322322
Pden_4177		Mannitol dehydrogenase, C-terminal domain		-2.37	3.98E-04	0.003908229
Pden_4178		mannitol ABC transporter ATP-binding protein / sorbitol ABC transporter ATP-binding protein		-2.58	1.66E-04	0.002544325
Pden_4179		sorbitol ABC transporter membrane protein / mannitol ABC transporter membrane protein		-2.67	8.07E-05	0.001856381
Pden_4180		mannitol ABC transporter membrane protein / sorbitol ABC transporter membrane protein		-3.42	1.99E-04	0.002746744
Pden_4181		mannitol-binding protein / sorbitol-binding protein		-3.05	5.30E-05	0.001560115
Pden_4182	*queF*	GTP cyclohydrolase I		3.43	7.48E-05	0.001812039
Pden_4183	*queE*	preQ(0) biosynthesis protein QueE		4.41	5.50E-06	4.66E-04
Pden_4184		6-pyruvoyl tetrahydropterin synthase and hypothetical protein		4.20	3.41E-06	3.39E-04
Pden_4185	*queC*	exsB protein		4.37	2.25E-05	0.001055027

Pden_4187	*ugpC*	carbohydrate ABC transporter ATP-binding protein, CUT1 family	-1.33	0.001645176	0.009296344
Pden_4188	*ugpE*	carbohydrate ABC transporter membrane protein 2, CUT1 family	-2.42	0.001449945	0.008636553
Pden_4189	*ugpA*	carbohydrate ABC transporter membrane protein 1, CUT1 family	-2.66	4.28E-05	0.001441542
Pden_4190	*ugpB*	carbohydrate ABC transporter substrate-binding protein, CUT1 family	-2.83	1.13E-05	7.46E-04
Pden_4210		response regulator receiver protein	-2.20	0.001321453	0.0080999
Pden_4211		SSS sodium solute transporter superfamily	-2.78	6.54E-04	0.005207841
Pden_4213	*acs*	acetyl-coenzyme A synthetase	-2.83	1.12E-05	7.46E-04
Pden_4221		hypothetical protein	3.24	0.001095282	0.007146149
Pden_4226		3-octaprenyl-4hydroxybenzoate decarboxylase	2.53	0.00181163	0.009873285
Pden_4227		conserved hypothetical protein	3.27	4.60E-04	0.004272675
Pden_4228	*ubiU*	peptidase U32	2.33	3.68E-04	0.003739575
Pden_4229	*ubiV*	peptidase U32	1.73	5.77E-04	0.004898771
Pden_4241		drug resistance transporter, Bcr/CflA subfamily	2.99	0.001409382	0.008444617
Pden_4253		ATP:cob(I)alamin adenosyltransferase	-2.59	3.89E-04	0.003876944
Pden_4255		molecular chaperone, DnaK	4.03	7.82E-05	0.001836392
Pden_4257	*xdhA*	molybdopterin dehydrogenase, FAD-binding protein	-2.80	8.87E-04	0.006300586
Pden_4258	*xdhB*	xanthine dehydrogenase, molybdenum binding subunit apoprotein / Xanthine oxidase	-3.17	4.71E-04	0.004312253
Pden_4259	*xdhC*	molybdenum cofactor sulfurylase	-2.82	1.36E-04	0.002288892
Pden_4260	*guaD*	guanine deaminase	-2.83	5.08E-04	0.004507426
Pden_4261		acetone carboxylase, gamma subunit	-7.27	3.86E-04	0.003865259
Pden_4262		acetone carboxylase, alpha subunit	-7.83	4.57E-04	0.004269771
Pden_4263		acetone carboxylase, beta subunit	-7.96	2.11E-04	0.002796602
Pden_4273	*tpa*	aminotransferase	-4.77	2.39E-04	0.002957052
Pden_4274		transcriptional regulator, GntR family	-3.45	2.09E-04	0.00277756
Pden_4276		conserved hypothetical protein 698	1.91	0.001697942	0.009488612
Pden_4289		conserved hypothetical protein	5.09	4.83E-05	0.0014723
Pden_4290		conserved hypothetical protein	4.83	1.32E-04	0.002284717
Pden_4291	*dnaE2*	error-prone DNA polymerase, DnaE-like protein	3.52	5.62E-06	4.66E-04
Pden_4295		ATP-dependent DNA helicase, Rep family	1.33	3.45E-04	0.003644311
Pden_4303	*rpsD*	SSU ribosomal protein S4P	3.04	4.67E-04	0.00430718
Pden_4312		FAD linked oxidase domain protein	1.87	0.001148076	0.007373321
Pden_4313		GCN5-related N-acetyltransferase	2.16	0.001639944	0.009279383
Pden_4323	*dprA*	DNA protecting protein DprA	2.35	1.24E-04	0.002256524
Pden_4330		phosphate ABC transporter substrate-binding protein, PhoT family	1.62	2.24E-04	0.002864856
Pden_4334		amino acid/polyamine/organocation transporter, APC superfamily	-2.73	1.41E-04	0.002302411
Pden_4338		PE-PGRS family protein	-1.78	0.001008111	0.006805424
Pden_4341	*cysA*	sulfate ABC transporter, ATPase subunit	2.70	6.39E-04	0.005142504
Pden_4342	*cysW*	sulfate ABC transporter, inner membrane subunit CysW	2.27	4.62E-04	0.004272675
Pden_4347		Radical SAM domain protein	-1.74	1.82E-04	0.002636701
Pden_4352		5-oxoprolinase (ATP-hydrolyzing)	-1.73	1.86E-04	0.002661543
Pden_4353		5-oxoprolinase (ATP-hydrolyzing)	-1.45	0.001162792	0.007442751
Pden_4366		DEAD/DEAH box helicase domain protein	2.98	1.59E-05	8.87E-04
Pden_4368		sodium:dicarboxylate symporter	-4.87	1.01E-04	0.00205033
Pden_4373		TonB-dependent siderophore receptor	-2.23	6.05E-05	0.001630492
Pden_4382		TonB-dependent receptor	2.17	3.49E-05	0.001297726
Pden_4383		periplasmic binding protein	2.73	4.02E-05	0.001414323
Pden_4384		transport system permease protein	3.46	2.23E-04	0.002861702
Pden_4385		ABC transporter related protein	3.62	7.65E-05	0.001836392
Pden_4387		carbohydrate ABC transporter substrate-binding protein, CUT1 family	-2.90	4.41E-05	0.001441542
Pden_4388		conserved hypothetical protein	-1.53	3.23E-04	0.003558939
Pden_4397	*glcF*	protein of unknown function DUF224, cysteine-rich region domain protein	-1.94	3.50E-04	0.003644311
Pden_4401		Uncharacterized protein UPF0065	-4.38	3.27E-04	0.003573445
Pden_4402		protein of unknown function DUF112, transmembrane	-4.61	3.32E-04	0.003583012
Pden_4403		protein of unknown function DUF1468	-4.92	2.01E-04	0.002747242
Pden_4404		Uncharacterized protein UPF0065	-5.72	2.51E-04	0.003045163
Pden_4410		transcriptional regulator, AraC family	-1.52	3.47E-04	0.003644311
Pden_4417	*lepA*	GTP-binding protein LepA	1.40	2.82E-04	0.003316655
Pden_4422		alkylhydroperoxidase like protein, AhpD family	2.08	3.07E-04	0.003457321
Pden_4427	*glgC*	glucose-1-phosphate adenylyltransferase	-1.53	3.96E-04	0.003908229
Pden_4435		aminodeoxychorismate synthase, subunit I	3.56	3.20E-04	0.003546894
Pden_4441		carbohydrate ABC transporter membrane protein 1, CUT1 family	3.55	7.93E-04	0.005842821
Pden_4442		carbohydrate ABC transporter substrate-binding protein, CUT1 family	3.32	9.84E-04	0.006721522
Pden_4447		extracellular solute-binding protein, family 1	-2.05	6.78E-04	0.005324
Pden_4458		RNA binding S1 domain protein	1.78	1.94E-04	0.002717725
Pden_4463		Adenylosuccinate lyase	1.43	8.07E-04	0.005905824
Pden_4464		flagellar motor switch protein FliG	3.10	5.59E-04	0.004802439
Pden_4480		acyl-CoA dehydrogenase domain protein	-1.48	2.93E-04	0.003351226
Pden_4491	*murB*	UDP-N-acetylmuramate dehydrogenase	2.10	5.96E-04	0.004981631
Pden_4502		ABC polyamine transporter, periplasmic substrate-binding protein	-1.90	9.55E-05	0.001988942
Pden_4510	*ubiB*	2-octaprenylphenol hydroxylase	1.34	3.16E-04	0.00352866
Pden_4511	*ubiE*	2-octaprenyl-6-methoxy-1,4-benzoquinone methylase	1.20	0.001078959	0.007094506
Pden_4515		3-demethylubiquinone-9 3-methyltransferase	-2.34	0.001002301	0.006795278
Pden_4516		3-demethylubiquinone-9 3-methyltransferase	-2.06	1.06E-04	0.002072833
Pden_4519		Activator of Hsp90 ATPase 1 family protein	-2.23	6.99E-05	0.001761223
Pden_4520		Glyoxalase/bleomycin resistance protein/dioxygenase	-1.48	2.04E-04	0.002760804
Pden_4522		Glyoxalase/bleomycin resistance protein/dioxygenase	1.19	0.001193501	0.007572298
Pden_4526		Methyltransferase type 11	1.25	0.001236921	0.007741079
Pden_4527		transcriptional regulator, MarR family	1.54	3.55E-04	0.003665149
Pden_4529		Na+/solute symporter	-2.51	3.73E-05	0.001329308
Pden_4530		ketopantoate reductase	-2.24	1.14E-04	0.002162073
Pden_4531		protein of unknown function DUF849	-2.95	0.001798904	0.009814496

Pden_4543		N-formylglutamate amidohydrolase	-1.83	0.001814479	0.009878182
Pden_4544		Tripartite ATP-independent periplasmic transporter, DctQ component	-2.45	0.00103384	0.006914571
Pden_4545		TRAP C4-dicarboxylate transport system permease DctM subunit	-2.00	1.41E-04	0.002302411
Pden_4547		L-glutamine synthetase	-2.31	6.25E-04	0.005101822
Pden_4550	*acs*	acetyl-coenzyme A synthetase	-2.84	1.20E-05	7.68E-04
Pden_4552		Na+/solute symporter	-4.38	1.31E-05	8.08E-04
Pden_4553		cyclic nucleotide-binding protein	-4.12	1.60E-05	8.87E-04
Pden_4566		protein of unknown function DUF6, transmembrane	2.31	2.51E-05	0.001123538
Pden_4568		hypothetical protein	2.21	1.18E-04	0.002186091
Pden_4569		hypothetical protein	2.48	9.25E-05	0.001988331
Pden_4578		Cupin 2, conserved barrel domain protein	1.81	3.73E-04	0.003774183
Pden_4591		Rhodanese domain protein	-1.68	1.31E-04	0.002284717
Pden_4592		uncharacterized peroxidase-related enzyme	-2.20	6.80E-04	0.005331091
Pden_4603		putative ABC transporter binding protein component	-3.12	2.36E-04	0.002938416
Pden_4604		transcriptional regulator, IclR family	-1.32	0.001565968	0.009040019
Pden_4606		Radical SAM domain protein	3.89	4.39E-06	3.97E-04
Pden_4607		Uracil-DNA glycosylase superfamily	3.95	9.25E-06	6.69E-04
Pden_4608		putative general stress protein 26	1.65	2.42E-04	0.002976217
Pden_4609		phospholipase D/Transphosphatidylase	1.92	5.14E-04	0.004513649
Pden_4616	*hydA*	dihydropyrimidinase	-1.70	4.75E-04	0.004327427
Pden_4625		Glu/Leu/Phe/Val dehydrogenase, dimerization region	-2.84	1.96E-04	0.002717725
Pden_4626		protein of unknown function DUF161	-3.08	1.76E-04	0.002605071
Pden_4638		hypothetical protein	1.80	2.91E-04	0.003349127
Pden_4641		conserved hypothetical protein	-2.38	0.001224623	0.007696203
Pden_4642		conserved hypothetical protein	-2.24	1.15E-04	0.002162073
Pden_4649		extracellular solute-binding protein, family 1	-2.62	1.47E-05	8.86E-04
Pden_4650		binding-protein-dependent transport systems inner membrane component	-2.32	3.49E-04	0.003644311
Pden_4667		amino acid ABC transporter substrate-binding protein, PAAT family	-2.75	7.70E-04	0.005770981
Pden_4670		amino acid ABC transporter membrane protein 1, PAAT family	-2.07	0.001508479	0.008849146
Pden_4677		2OG-Fe(II) oxygenase	-1.50	9.66E-04	0.006681372
Pden_4688		YbaK/prolyl-tRNA synthetase associated region	1.81	6.02E-04	0.005000875
Pden_4689		hypothetical protein	1.51	0.001545068	0.008981262
Pden_4691	*pssA*	CDP-diacylglycerol--serine O-phosphatidyltransferase	1.09	0.001579159	0.009052351
Pden_4692	*psd*	phosphatidylserine decarboxylase related protein	1.72	0.001391469	0.008380808
Pden_4693		uracil-xanthine permease	2.69	2.62E-05	0.001142137
Pden_4721	*napA*	periplasmic nitrate reductase subunit NapA apoprotein	-2.07	6.91E-04	0.005364018
Pden_4723		periplasmic nitrate reductase subunit NapC	-2.36	4.47E-04	0.004200412
Pden_4726		MscS Mechanosensitive ion channel	2.91	1.79E-04	0.002619004
Pden_4730	*mauB*	methylamine dehydrogenase heavy chain	-3.14	1.65E-04	0.002535748
Pden_4751		FAD dependent oxidoreductase	-1.92	5.57E-05	0.001611959
Pden_4752		aldehyde dehydrogenase	-2.20	1.73E-04	0.0025947
Pden_4753		dihydrodipicolinate synthetase	-2.52	1.72E-05	9.27E-04
Pden_4754		conserved hypothetical protein	1.97	3.49E-04	0.003644311
Pden_4780		phosphonate metabolism	-1.09	0.001256693	0.007826119
Pden_4784	*phnE*	phosphonate ABC transporter, inner membrane subunit	2.29	4.15E-04	0.004014627
Pden_4785	*phnE*	phosphonate ABC transporter, inner membrane subunit	3.79	0.001427143	0.008540929
Pden_4786	*phnD*	phosphonate ABC transporter, periplasmic phosphonate-binding protein	2.00	8.57E-04	0.006160708
Pden_4793		amino acid/amide ABC transporter ATP-binding protein 2, HAAT family	-2.62	7.91E-05	0.001836392
Pden_4794		amino acid/amide ABC transporter ATP-binding protein 1, HAAT family	-2.80	1.04E-04	0.002056763
Pden_4795		amino acid/amide ABC transporter membrane protein 2, HAAT family	-3.31	3.64E-05	0.001320801
Pden_4796		amino acid/amide ABC transporter membrane protein 1, HAAT family	-4.04	1.75E-04	0.002605071
Pden_4797		amino acid/amide ABC transporter substrate-binding protein, HAAT family	-4.74	9.27E-05	0.001988331
Pden_4798	*paaN*	phenylacetic acid degradation protein paaN	-1.94	2.33E-04	0.002932574
Pden_4799	*paaB*	Enoyl-CoA hydratase / short chain enoyl-CoA hydratase	-2.48	2.96E-04	0.003369347
Pden_4801	*paaF*	phenylacetate-CoA ligase	-3.60	9.50E-05	0.001988942
Pden_4806	*paaK*	phenylacetate-CoA oxygenase/reductase, PaaK subunit	-1.52	0.001548283	0.008983446
Pden_4807	*paaJ*	phenylacetate-CoA oxygenase, PaaJ subunit	-1.88	3.38E-04	0.003618025
Pden_4809	*paaB*	phenylacetate-CoA oxygenase, PaaH subunit	-2.70	0.001523683	0.008897814
Pden_4810	*paaA*	phenylacetate-CoA oxygenase, PaaG subunit	-2.19	1.25E-04	0.002256524
Pden_4813		Penicillin amidase	-3.96	3.37E-05	0.001281971
Pden_4814		conserved hypothetical protein	-2.51	7.44E-05	0.00180991
Pden_4815		conserved hypothetical protein	-2.15	5.99E-05	0.001626459
Pden_4816		TRAP C4-dicarboxylate transport system permease DctM subunit	-2.53	2.00E-04	0.002746744
Pden_4817		TRAP dicarboxylate transporter, DctP subunit	-4.07	7.12E-06	5.55E-04
Pden_4818		transcriptional regulator, IclR family	-2.84	8.87E-04	0.006300586
Pden_4819		Thiolase	-2.92	9.95E-05	0.002048237
Pden_4820		AMP-dependent synthetase and ligase	-2.34	5.09E-04	0.004509758
Pden_4821		conserved hypothetical protein	-2.13	9.47E-04	0.006621591
Pden_4822		short-chain dehydrogenase/reductase SDR	-2.32	0.001020549	0.006863897
Pden_4829		periplasmic binding protein	2.21	5.26E-04	0.004589558
Pden_4830		transport system permease protein	3.14	3.78E-04	0.003809005
Pden_4859	*hypF*	proline racemase	-1.60	0.001499962	0.008820336
Pden_4861		amino acid ABC transporter ATP-binding protein, PAAT family	-1.20	0.00117731	0.007497765
Pden_4862		amino acid ABC transporter membrane protein 2, PAAT family	-1.83	1.07E-04	0.002081794
Pden_4863		amino acid ABC transporter membrane protein 1, PAAT family	-2.45	6.16E-04	0.005082021
Pden_4864		amino acid ABC transporter substrate-binding protein, PAAT family	-1.95	5.77E-05	0.001613164
Pden_4871	*proC*	pyrroline-5-carboxylate reductase	2.62	0.001650719	0.00931727
Pden_4872		FAD linked oxidase domain protein	1.79	0.001298054	0.008020086
Pden_4881		Substrate-binding region of ABC-type glycine betaine transport system	-2.21	5.86E-04	0.004954272
Pden_4888		Substrate-binding region of ABC-type glycine betaine transport system	-1.69	8.01E-04	0.005884409
Pden_4889		Nitrilase/cyanide hydratase and apolipoprotein N-acyltransferase	-1.12	0.001640341	0.009279383

Pden_4911		extracellular solute-binding protein	-3.38	3.22E-04	0.003554006
Pden_4941		Betaine-aldehyde dehydrogenase	-2.55	5.11E-05	0.001530504
Pden_4942		thiamine pyrophosphate enzyme domain protein TPP-binding protein	-2.14	6.10E-05	0.001632877
Pden_4943		extracellular solute-binding protein, family 1	-3.53	2.41E-04	0.002970463
Pden_4944		ABC transporter related protein	-3.47	5.23E-04	0.004572565
Pden_4945		binding-protein-dependent transport systems inner membrane component	-2.44	6.14E-05	0.001635888
Pden_4946		binding-protein-dependent transport systems inner membrane component	-2.28	2.41E-04	0.002970463
Pden_4976	*argE*	acetylornithine deacetylase / acetylornithine deacetylase ArgE, Metallo peptidase. MEROPS family M20A	-1.67	0.001016066	0.006849989
Pden_4992		Enoyl-CoA hydratase/isomerase	-2.21	5.22E-04	0.004571911
Pden_4994		amino acid/amide ABC transporter substrate-binding protein, HAAT family	-3.09	8.49E-05	0.001909873
Pden_4996		CoA-binding domain protein	-3.35	4.40E-05	0.001441542
Pden_4997		amino acid/amide ABC transporter ATP-binding protein 2, HAAT family	-4.69	1.62E-05	8.87E-04
Pden_4998		amino acid/amide ABC transporter ATP-binding protein 1, HAAT family	-4.19	5.70E-05	0.001613164
Pden_4999		amino acid/amide ABC transporter membrane protein 2, HAAT family	-4.38	1.49E-05	8.86E-04
Pden_5000		amino acid/amide ABC transporter membrane protein 1, HAAT family	-4.02	4.36E-06	3.97E-04
Pden_5002		Rhodanese domain protein	1.16	0.001293446	0.008005764
Pden_5004	*ssuB2*	ABC transporter related protein	2.54	1.28E-04	0.002273372
Pden_5005		binding-protein-dependent transport systems inner membrane component	3.24	1.42E-04	0.002302984
Pden_5006		aliphatic sulfonates family ABC transporter, periplasmic ligand-binding protein	3.15	1.26E-05	7.88E-04
Pden_5007		monooxygenase, NtaA/SnaA/SoxA family	3.67	7.26E-06	5.57E-04
Pden_5010		ABC transporter related protein	3.03	0.001172131	0.007483606
Pden_5011		binding-protein-dependent transport systems inner membrane component	2.47	9.52E-05	0.001988942
Pden_5012		ABC transporter substrate-binding protein	3.22	2.97E-04	0.003369347
Pden_5013		Alkanesulfonate monooxygenase	3.24	3.70E-05	0.001328541
Pden_5014		Acyl-CoA dehydrogenase, type 2, C-terminal domain	3.63	2.05E-04	0.002762396
Pden_5016		serine O-acetyltransferase	2.00	0.001256603	0.007826119
Pden_5017		conserved hypothetical protein	2.64	4.13E-05	0.001440375
Pden_5021		3-oxoadipate CoA-transferase beta subunit	1.66	7.28E-04	0.005551687
Pden_5022	*pcaF*	3-oxoadipyl-CoA thiolase	1.83	1.87E-04	0.002675066
Pden_5023		NAD-dependent epimerase/dehydratase	1.49	6.61E-04	0.005252026
Pden_5024		hypothetical protein	2.39	9.53E-04	0.006658331
Pden_5037		ABC transporter related protein	1.77	9.83E-04	0.006721522
Pden_5039		ABC-2 type transporter	1.78	0.001230242	0.007708806
Pden_5041		hypothetical protein	1.65	4.77E-04	0.004336923
Pden_5044		aldo/keto reductase	-1.04	0.001733671	0.009603475
Pden_5048		ABC transporter related protein	-2.17	5.56E-04	0.004791174
Pden_5049		binding-protein-dependent transport systems inner membrane component	-1.55	7.91E-04	0.005842821
Pden_5050		binding-protein-dependent transport systems inner membrane component	-1.59	2.50E-04	0.003045163
Pden_5051		polysaccharide deacetylase	-1.63	2.35E-04	0.002938416
Pden_5081		amino acid ABC transporter substrate-binding protein, PAAT family	-2.16	2.06E-04	0.002762396
Pden_5101		conserved hypothetical protein	2.79	9.02E-06	6.62E-04
Pden_5117		protein of unknown function DUF1234	3.11	5.12E-04	0.004509969
Pden_5118		conserved hypothetical protein	3.11	1.45E-04	0.00231305
Pden_5119		NADPH-dependent FMN reductase	2.42	5.07E-05	0.001528498
Pden_5120		hypothetical protein	3.26	1.83E-05	9.57E-04
Pden_5121		hypothetical protein	3.37	1.91E-05	9.76E-04
Pden_5124		hypothetical protein	3.38	5.66E-04	0.004855209
Pden_5125		putative monooxygenase protein	4.63	2.41E-06	2.65E-04
Pden_5126		binding-protein-dependent transport systems inner membrane component	5.15	3.31E-05	0.001270903
Pden_5127		transcriptional regulator, Fis family	4.70	2.75E-05	0.001152121
Pden_5128		ABC transporter related protein	4.35	1.47E-04	0.002325272
Pden_5132		efflux transporter, RND family, MFP subunit	2.63	7.13E-04	0.005462944
Pden_5133	*macB3*	ABC transporter related protein	2.79	0.001659813	0.009345864

Table S4. Lists of differentially expressed genes between Δ*recA* with MMC vs. Δ*recA* without MMC.

gene_id	name	annotation	PhiSpy_phage_region	logFC	PValue	FDR
Pden_0003	*mnmE (trmE)*	tRNA modification GTPase trmE		2.32	3.05E-05	7.09E-04
Pden_0010	*rph*	RNAse PH		1.29	6.49E-04	0.003220189
Pden_0011	*rdgB*	dITPase		1.94	0.001666094	0.00593209
Pden_0012		Endoribonuclease L-PSP		1.92	1.45E-04	0.001413281
Pden_0013		putative oxygen-independent coproporphyrinogen III oxidase		2.00	8.13E-05	0.00102897
Pden_0017	*clpP*	ATP-dependent Clp protease proteolytic subunit ClpP		-1.75	0.00224824	0.007287348
Pden_0018		GCN5-related N-acetyltransferase		-1.72	7.95E-04	0.003649359
Pden_0019	*fghA*	Carboxylesterase		-1.95	0.001383444	0.005219358
Pden_0020		Pyrrolo-quinoline quinone		-4.03	7.15E-04	0.00340186
Pden_0021		hypothetical protein		-3.02	1.51E-04	0.001447668
Pden_0022		amino acid ABC transporter substrate-binding protein, PAAT family		-2.53	4.84E-04	0.002708578
Pden_0024		conserved hypothetical protein		-2.98	1.12E-05	6.87E-04
Pden_0026		amino acid/amide ABC transporter substrate-binding protein, HAAT family		-4.03	3.19E-05	7.09E-04
Pden_0027		40-residue YVTN family beta-propeller repeat protein		-4.16	1.81E-05	6.87E-04
Pden_0028		hypothetical protein		-4.70	2.55E-04	0.001883264
Pden_0029		ABC transporter related protein		-3.66	6.06E-05	9.28E-04
Pden_0030		ABC-2 type transporter		-2.98	9.77E-05	0.001134352
Pden_0031		conserved hypothetical protein		-1.38	0.001295901	0.004974334
Pden_0042	*thiD*	hydroxymethylpyrimidine kinase / phosphomethylpyrimidine kinase		2.69	3.78E-04	0.002311838
Pden_0046		two component transcriptional regulator, winged helix family		-2.09	3.12E-05	7.09E-04
Pden_0047		PAS/PAC sensor hybrid histidine kinase		-2.55	1.06E-05	6.87E-04
Pden_0048		AMP-dependent synthetase and ligase		-4.73	5.05E-05	9.00E-04
Pden_0049		amino acid/amide ABC transporter ATP-binding protein 1, HAAT family		-4.65	4.73E-06	6.87E-04
Pden_0050		amino acid/amide ABC transporter membrane protein 1, HAAT family		-4.10	5.20E-05	9.01E-04
Pden_0051		amino acid/amide ABC transporter membrane protein 2, HAAT family		-4.11	1.98E-04	0.001639291
Pden_0052		amino acid/amide ABC transporter substrate-binding protein, HAAT family		-5.27	7.51E-06	6.87E-04
Pden_0053		amino acid/amide ABC transporter ATP-binding protein 2, HAAT family		-5.03	5.22E-06	6.87E-04
Pden_0054		phenylacetate-CoA ligase, putative		-3.61	1.61E-05	6.87E-04
Pden_0056		N-formylglutamate amidohydrolase		-1.10	0.001287242	0.004956126
Pden_0057		SPFH domain, Band 7 family protein		-1.66	9.08E-04	0.00396488
Pden_0067	*tnpB*	IS66 Orf2 family protein		6.11	2.25E-05	6.88E-04
Pden_0068		transposase IS66		4.22	5.36E-06	6.87E-04
Pden_0072		CHAD domain containing protein		1.88	1.14E-04	0.001205609
Pden_0074		adenylate cyclase		1.16	0.002547446	0.007991152
Pden_0077		transposase		2.74	3.81E-05	7.91E-04
Pden_0084		hypothetical protein		2.98	0.002334265	0.007479989
Pden_0085		hypothetical protein		3.05	0.001176029	0.004659024
Pden_0087	*pseF*	acylneuraminate cytidylyltransferase		1.44	0.001049593	0.004359385
Pden_0088		flagellin modification protein FlmD		1.57	3.91E-04	0.002370473
Pden_0097		hypothetical protein		-2.00	1.62E-04	0.001494676
Pden_0098		Conjugal transfer TraD family protein		-3.07	3.11E-05	7.09E-04
Pden_0099		conserved hypothetical protein		-2.57	2.69E-05	7.01E-04
Pden_0100	*traA*	plasmid mobilization system relaxase		1.53	0.002473962	0.007804156
Pden_0108		aldo/keto reductase		2.05	0.001435982	0.005357684
Pden_0116	*catB*	transferase hexapeptide repeat containing protein		1.94	1.52E-04	0.001450721
Pden_0117		hypothetical protein		-1.10	0.002680201	0.008264226
Pden_0120		DNA methylase N-4/N-6 domain protein		3.30	2.94E-04	0.002042562
Pden_0126		hypothetical protein		3.94	1.17E-05	6.87E-04
Pden_0127		conserved hypothetical protein		4.11	3.51E-04	0.002253976
Pden_0128		hypothetical protein		1.86	4.66E-04	0.002638816
Pden_0130		transcriptional regulator, XRE family		1.12	0.00217453	0.00714912
Pden_0131		hypothetical protein		5.03	7.26E-06	6.87E-04
Pden_0132		putative replication protein A		3.62	2.63E-05	7.00E-04
Pden_0133		conserved hypothetical protein		2.95	2.00E-05	6.87E-04
Pden_0134		transposase IS116/IS110/IS902 family protein		-1.94	5.50E-05	9.01E-04
Pden_0136		protein of unknown function DUF736		-1.23	0.0020275	0.006816224
Pden_0138		hypothetical protein		-1.51	3.31E-04	0.002212274
Pden_0157		transcriptional regulator, LysR family		-1.99	7.42E-04	0.003490386
Pden_0160		acriflavin resistance protein		2.37	1.89E-05	6.87E-04
Pden_0171		monooxygenase, FAD-binding protein		-2.60	0.001413867	0.005302526
Pden_0172		benzoate-CoA ligase family		-2.95	3.18E-04	0.002153186
Pden_0173		fumarylacetoacetate (FAA) hydrolase		-3.33	0.001590682	0.005776426
Pden_0174	*maiA*	maleylacetoacetate isomerase		-2.89	3.99E-04	0.002394246
Pden_0175		amino acid/amide ABC transporter substrate-binding protein, HAAT family		-4.26	0.001322502	0.00504584
Pden_0176		amino acid/amide ABC transporter membrane protein 1, HAAT family		-2.03	0.003114475	0.009239266
Pden_0177		amino acid/amide ABC transporter membrane protein 2, HAAT family		-2.18	0.002603158	0.00810067
Pden_0179		amino acid/amide ABC transporter ATP-binding protein 2, HAAT family		-2.15	0.001280585	0.004941769
Pden_0180		aldehyde dehydrogenase		-3.38	7.30E-05	9.81E-04
Pden_0181		glucose-methanol-choline oxidoreductase		-3.46	3.43E-04	0.002238614
Pden_0182		RNAse Z		-2.31	0.001187224	0.004688702
Pden_0187		transcriptional regulator, MarR family		-2.83	1.06E-05	6.87E-04
Pden_0188		regulatory protein, IclR		-1.57	0.002344826	0.007499592
Pden_0189		TRAP C4-dicarboxylate transport system permease DctM subunit		-2.58	1.82E-04	0.001544858
Pden_0190		Tripartite ATP-independent periplasmic transporter, DctQ component		-3.65	7.96E-05	0.001019694
Pden_0192		short-chain dehydrogenase/reductase SDR		-3.36	6.76E-05	9.57E-04
Pden_0193		TRAP dicarboxylate transporter- DctP subunit		-3.73	0.001233407	0.004829652
Pden_0194		conserved hypothetical protein		-1.46	0.001001808	0.004219763
Pden_0195		L-carnitine dehydratase/bile acid-inducible protein F		-2.02	6.81E-04	0.003308863
Pden_0196		short-chain dehydrogenase/reductase SDR		-1.83	8.46E-04	0.003801247
Pden_0201		3-hydroxyacyl-CoA dehydrogenase		2.57	0.001993578	0.006751497
Pden_0203		luciferase family protein		-3.15	6.59E-06	6.87E-04
Pden_0204	*ribB*	3,4-dihydroxy-2-butanone 4-phosphate synthase		-1.81	0.002977608	0.008946962
Pden_0205		hypothetical protein		-2.48	0.001354214	0.00513587
Pden_0206		short-chain dehydrogenase/reductase SDR		-4.03	1.81E-05	6.87E-04
Pden_0207		alpha/beta hydrolase fold protein		-1.57	0.00253914	0.007979929

Pden_0211		transcriptional regulator, MerR family		-1.32	0.001075397	0.004419427
Pden_0218		transcriptional regulator, LysR family		-1.72	1.96E-04	0.001631407
Pden_0219		hypothetical protein		-1.27	0.001082511	0.004432197
Pden_0220		luciferase family protein		-2.56	5.51E-05	9.01E-04
Pden_0221		conserved hypothetical protein		-1.41	3.58E-04	0.002285725
Pden_0224		sigma54 specific transcriptional regulator, Fis family		-2.94	3.37E-05	7.29E-04
Pden_0233		membrane protein-like protein		1.69	1.92E-04	0.001609815
Pden_0234		hypothetical protein		1.85	0.001449441	0.005395971
Pden_0235		oligopeptide/dipeptide ABC transporter, ATPase subunit		-2.25	1.13E-04	0.001195179
Pden_0236		oligopeptide/dipeptide ABC transporter, ATPase subunit		-2.34	5.15E-05	9.01E-04
Pden_0237		binding-protein-dependent transport systems inner membrane component		-3.18	3.62E-04	0.002298137
Pden_0238		binding-protein-dependent transport systems inner membrane component		-3.57	1.19E-04	0.001240013
Pden_0239		extracellular solute-binding protein, family 5		-3.45	3.10E-04	0.002120136
Pden_0242		iron-containing alcohol dehydrogenase		-2.72	6.31E-05	9.35E-04
Pden_0243		succinate semialdehyde dehydrogenase		-2.09	0.001035715	0.004326587
Pden_0244		Mandelate racemase/muconate lactonizing enzyme, C-terminal domain protein		-2.18	1.85E-04	0.001572457
Pden_0245		5-carboxymethyl-2-hydroxymuconate delta-isomerase		-1.88	3.92E-04	0.002370473
Pden_0248		monosaccharide ABC transporter ATP-binding protein, CUT2 family		1.26	0.002386871	0.00759091
Pden_0255		hypothetical protein		-3.17	2.78E-04	0.001990943
Pden_0270		gene transfer agent (GTA) like protein		1.84	9.92E-05	0.001137092
Pden_0279		transcriptional regulator, GntR family		-1.71	0.001470674	0.005462966
Pden_0280	*ehuA*	hydroxyectoine ABC transporter ATP-binding protein / ectoine ABC transporter ATP-binding protein		-4.07	4.65E-04	0.002634998
Pden_0281	*ehuB*	hydroxyectoine-binding protein / ectoine-binding protein		-4.32	4.92E-05	8.92E-04
Pden_0282	*ehuC*	hydroxyectoine ABC transporter membrane protein / ectoine ABC transporter membrane protein		-4.17	5.54E-05	9.01E-04
Pden_0283	*ehuD*	hydroxyectoine ABC transporter membrane protein / ectoine ABC transporter membrane protein		-3.25	1.67E-04	0.001505274
Pden_0284		Asp/Glu racemase		-2.73	5.45E-04	0.002899708
Pden_0285	*eutB*	Pyridoxal-5'-phosphate-dependent enzyme, beta subunit		-2.79	2.09E-04	0.001676926
Pden_0286		Ornithine cyclodeaminase		-3.08	4.35E-04	0.002539593
Pden_0287	*doeA*	peptidase M24		-3.34	2.63E-04	0.00192913
Pden_0288	*doeB*	Succinylglutamate desuccinylase/aspartoacylase		-2.66	0.002072324	0.006920961
Pden_0289		succinate semialdehyde dehydrogenase		-2.95	5.93E-04	0.003059768
Pden_0290		aminotransferase		-3.40	8.77E-05	0.001067537
Pden_0291		Alcohol dehydrogenase, zinc-binding domain protein		-2.40	0.001081179	0.004432197
Pden_0293		hypothetical protein		2.01	0.003371994	0.009800462
Pden_0294		Hemolysin-type calcium-binding region		1.43	5.69E-04	0.002981835
Pden_0296		TonB-dependent receptor		-1.24	0.002057742	0.006885889
Pden_0300		periplasmic binding protein		-1.36	7.21E-04	0.00341488
Pden_0304		transcriptional regulator, LysR family		2.98	6.87E-04	0.00331005
Pden_0310		two component transcriptional regulator, winged helix family		-1.55	0.001024247	0.00429285
Pden_0318		transcriptional regulator, GntR family		-1.38	0.001033855	0.004323912
Pden_0319		conserved hypothetical protein		-2.65	2.81E-04	0.002001287
Pden_0326		luciferase family protein		-1.66	0.00110441	0.004476884
Pden_0342		DNA polymerase III beta subunit family protein		1.95	0.001117084	0.004513404
Pden_0344		hypothetical protein		-2.69	1.56E-05	6.87E-04
Pden_0345		hypothetical protein		-1.08	0.002912148	0.008790037
Pden_0347		hypothetical protein		-4.15	0.003327307	0.009722909
Pden_0357		hypothetical protein	1	2.47	0.001056745	0.004364031
Pden_0358		Resolvase helix-turn-helix domain protein	1	4.07	6.58E-06	6.87E-04
Pden_0359		phage Terminase	1	4.01	1.32E-05	6.87E-04
Pden_0360		phage portal protein, HK97 family	1	3.49	1.19E-05	6.87E-04
Pden_0361		peptidase S14, ClpP	1	2.35	1.68E-04	0.001505274
Pden_0362		phage major capsid protein, HK97 family	1	1.86	3.71E-04	0.002309828
Pden_0364		hypothetical protein	1	1.75	0.002132275	0.00705609
Pden_0365		hypothetical protein	1	2.23	0.001053999	0.004364031
Pden_0368		hypothetical protein	1	-5.23	0.002212077	0.007225642
Pden_0376		hypothetical protein	1	2.08	4.93E-04	0.002738506
Pden_0377		hypothetical protein	1	2.16	4.53E-04	0.002599236
Pden_0380		hypothetical protein	1	2.28	5.87E-04	0.003045698
Pden_0387		hypothetical protein		-1.04	0.001659001	0.005923996
Pden_0391		hypothetical protein		-1.86	0.002038343	0.006848131
Pden_0393		hypothetical protein		-3.56	7.42E-05	9.87E-04
Pden_0396	*mtaB*	MiaB-like tRNA modifying enzyme		1.04	0.002946992	0.008870761
Pden_0400	*dxs*	1-deoxy-D-xylulose-5-phosphate synthase		1.26	0.002002197	0.006771624
Pden_0404	*amn*	AMP nucleosidase		-2.11	5.52E-05	9.01E-04
Pden_0417		protein of unknown function DUF1491		1.37	3.77E-04	0.002311838
Pden_0425		Electron-transferring-flavoprotein dehydrogenase		-1.42	3.54E-04	0.002264122
Pden_0439		hypothetical protein		-1.40	0.001522886	0.00560347
Pden_0440	*leuB*	3-isopropylmalate dehydrogenase		-1.29	0.002968734	0.008925594
Pden_0444		conserved hypothetical protein		-1.80	0.002811521	0.008560656
Pden_0446		zinc finger, CHC2-family protein		-2.59	0.002284619	0.007362843
Pden_0447		hypothetical protein		-3.05	2.71E-04	0.001969565
Pden_0448		conserved hypothetical protein		-3.55	0.001235838	0.004835431
Pden_0449		Exonuclease, RNase T and DNA polymerase III		-3.61	0.001132008	0.004557929
Pden_0450		conserved hypothetical protein		-1.08	0.002201684	0.007205642
Pden_0461	*istA*	Integrase, catalytic region		-2.50	2.88E-05	7.03E-04
Pden_0462		IstB domain protein ATP-binding protein		-2.73	1.13E-05	6.87E-04
Pden_0463		Recombinase		-1.47	0.001227351	0.004813383
Pden_0464		transposase IS116/IS110/IS902 family protein		-2.32	6.37E-05	9.35E-04
Pden_0467		putative spermidine/putrescine-binding protein		-4.37	0.001568718	0.005718083
Pden_0468		binding-protein-dependent transport systems inner membrane component		-4.50	3.43E-04	0.002238614
Pden_0469		binding-protein-dependent transport systems inner membrane component		-3.90	0.001607477	0.005813326
Pden_0471		protein of unknown function DUF849		-3.12	0.001837734	0.006359843
Pden_0472		5-oxoprolinase (ATP-hydrolyzing)		-3.37	8.29E-06	6.87E-04
Pden_0473		Hydantoinase B/oxoprolinase		-3.53	6.26E-06	6.87E-04
Pden_0474		short-chain dehydrogenase/reductase SDR		-3.28	4.94E-06	6.87E-04
Pden_0475		hypothetical protein		-2.99	2.93E-05	7.06E-04
Pden_0476		thioesterase superfamily protein		-2.72	1.20E-04	0.00124867

Pden_0477		transcriptional regulator, XRE family	-2.08	0.001814883	0.006297979
Pden_0494		NUDIX hydrolase	3.78	8.64E-04	0.003825889
Pden_0495		response regulator receiver protein	2.26	8.00E-05	0.001019694
Pden_0507		chaperone DnaJ domain protein	-1.10	0.001610262	0.005813604
Pden_0514		Sarcosine oxidase, delta subunit, heterotetrameric	-1.30	6.63E-04	0.003258025
Pden_0515		sarcosine oxidase, alpha subunit family	-1.32	0.001578469	0.005745354
Pden_0524		purine nucleotide phosphorylase	-2.65	1.35E-04	0.001332887
Pden_0525		nucleoside ABC transporter membrane protein	-2.79	4.41E-05	8.44E-04
Pden_0526		nucleoside ABC transporter membrane protein	-2.56	5.56E-04	0.002936497
Pden_0527		nucleoside ABC transporter ATP-binding protein	-2.83	2.18E-04	0.001704793
Pden_0528		nucleoside-binding protein	-3.89	6.56E-06	6.87E-04
Pden_0540	*glyS*	glycyl-tRNA synthetase beta chain	1.19	0.001770983	0.006171016
Pden_0543		dihydroneopterin aldolase	1.19	0.001294413	0.004972392
Pden_0548		ATPase associated with various cellular activities, AAA_5	-1.24	8.28E-04	0.003758457
Pden_0551	*lpdA*	dihydrolipoamide dehydrogenase	-1.40	6.24E-04	0.003147257
Pden_0554	*odhB*	2-oxoglutarate dehydrogenase E2 component	-1.04	0.001915719	0.006553571
Pden_0560		Hemolysin-type calcium-binding region	-1.46	5.73E-04	0.002995825
Pden_0564		NnrUfamily protein	-1.20	8.60E-04	0.003825716
Pden_0565		conserved hypothetical protein	-1.66	1.95E-04	0.001628537
Pden_0566		L-erythro-3-methylmalyl-CoA dehydratase	-1.43	2.24E-04	0.00171547
Pden_0570		conserved hypothetical protein	-1.38	0.001089632	0.004446969
Pden_0574		beta-lactamase domain protein	-3.16	2.11E-04	0.001679183
Pden_0579	*rlmJ*	protein of unknown function DUF519	1.92	9.62E-05	0.001121864
Pden_0590		membrane protein involved in aromatic hydrocarbon degradation	-4.42	6.02E-04	0.003082284
Pden_0592	*guaB*	inosine-5'-monophosphate dehydrogenase	1.10	0.002044273	0.006863498
Pden_0593		hypothetical protein	2.91	8.83E-06	6.87E-04
Pden_0594		Fmu (Sun) domain protein	1.93	0.003106892	0.009239266
Pden_0595		multi-sensor hybrid histidine kinase	3.11	4.61E-04	0.002628348
Pden_0596		conserved hypothetical protein	1.23	0.001639218	0.005877734
Pden_0600		putative glutamine amidotransferase	-1.82	1.31E-04	0.001314735
Pden_0601		hypothetical protein	3.04	3.72E-04	0.002309828
Pden_0605		thiamine diphosphokinase	1.40	5.75E-04	0.003000752
Pden_0606		binding-protein-dependent transport systems inner membrane component	-3.40	2.15E-04	0.001694513
Pden_0607		binding-protein-dependent transport systems inner membrane component	-3.66	7.78E-05	0.001009788
Pden_0608		extracellular solute-binding protein, family 5	-3.64	2.00E-05	6.87E-04
Pden_0609		ABC transporter related protein	-2.67	5.31E-05	9.01E-04
Pden_0610		amidohydrolase	-2.69	2.34E-05	6.88E-04
Pden_0611	*lpdA*	dihydrolipoamide dehydrogenase	-2.84	8.35E-05	0.001043896
Pden_0612		protein of unknown function DUF924	-1.68	2.85E-04	0.002013301
Pden_0615		putative bacterioferritin-associated ferredoxin	-2.60	0.003429049	0.009903205
Pden_0628		hypothetical protein	1.59	1.49E-04	0.001433489
Pden_0630		aromatic amino acid aminotransferase apoenzyme	-1.79	8.55E-05	0.001060694
Pden_0632	*smpB*	SsrA-binding protein	1.84	6.77E-05	9.57E-04
Pden_0633		thiamine pyrophosphate enzyme TPP binding domain protein	-2.13	0.0016626	0.005923996
Pden_0645	*def*	peptide deformylase	2.05	1.31E-04	0.001314195
Pden_0646	*def*	Peptide deformylase	1.38	4.56E-04	0.002608497
Pden_0648		aminotransferase, class I and II	-1.37	2.82E-04	0.002004634
Pden_0649		ErfK/YbiS/YcfS/YnhG family protein	-1.26	0.002052369	0.006881553
Pden_0656		phosphoadenylylsulfate reductase (thioredoxin)	-1.58	0.001624115	0.005840986
Pden_0659	*topA*	DNA topoisomerase I	1.33	3.61E-04	0.002294387
Pden_0660	*ggt*	gamma-glutamyltransferase 1, Threonine peptidase, MEROPS family T03	-2.31	8.31E-05	0.00104193
Pden_0661		protein of unknown function DUF488	1.62	0.00174496	0.006122271
Pden_0663		TRAP transporter, 4TM/12TM fusion protein	-2.26	2.07E-05	6.87E-04
Pden_0664		TRAP transporter solute receptor, TAXI family	-2.67	1.35E-05	6.87E-04
Pden_0665		phosphate transporter	1.37	0.001944579	0.00663437
Pden_0666		amino acid carrier protein	-1.23	0.002654426	0.008219791
Pden_0688	*cpoB*	conserved hypothetical protein	-1.65	3.37E-04	0.002227917
Pden_0690	*ftsH*	membrane protease FtsH catalytic subunit	-1.40	0.001231974	0.004827776
Pden_0691		chorismate mutase	1.61	1.99E-04	0.001641009
Pden_0702		multisubunit potassium/proton antiporter, PhaG subunit	-1.08	0.00331867	0.009712385
Pden_0706		protein of unknown function DUF6, transmembrane	1.94	1.25E-04	0.001283565
Pden_0707		inositol monophosphatase	1.64	1.97E-04	0.001637428
Pden_0716		amino acid ABC transporter substrate-binding protein, PAAT family	-2.02	0.001158529	0.004629544
Pden_0717		amino acid ABC transporter membrane protein 1, PAAT family	-2.16	3.53E-04	0.00226395
Pden_0718		amino acid ABC transporter membrane protein 2, PAAT family	-1.68	1.00E-04	0.001137092
Pden_0719		amino acid ABC transporter ATP-binding protein, PAAT family	-1.10	0.001094949	0.00445637
Pden_0721		Patatin	-2.54	7.63E-04	0.003564054
Pden_0725		TonB family protein	-2.40	0.001900571	0.00651055
Pden_0726	*exbD*	outer membrane transport energization protein ExbD	-3.19	2.73E-05	7.03E-04
Pden_0727	*exbB*	outer membrane transport energization protein ExbB	-2.24	6.16E-04	0.003133449
Pden_0738		transferase hexapeptide repeat containing protein	1.83	7.47E-04	0.003509395
Pden_0739		ABC transporter related protein	2.05	3.31E-04	0.002212274
Pden_0740		hypothetical protein	3.50	2.07E-05	6.87E-04
Pden_0742	*nusG*	transcription antitermination protein nusG	2.56	1.37E-05	6.87E-04
Pden_0744	*rplA*	LSU ribosomal protein L1P	1.69	5.99E-04	0.003074387
Pden_0745	*rplJ*	LSU ribosomal protein L10P	2.75	2.77E-04	0.001988805
Pden_0746	*rplL*	LSU ribosomal protein L12P / LSU ribosomal protein L12P	2.32	4.81E-04	0.002695821
Pden_0751		protein of unknown function DUF6, transmembrane	1.39	3.69E-04	0.002309828
Pden_0752		conserved hypothetical protein	1.39	0.001509555	0.005578741
Pden_0757		hypothetical protein	-2.78	1.93E-05	6.87E-04
Pden_0760	*rplD*	LSU ribosomal protein L4P	1.81	0.001073939	0.004417022
Pden_0765	*rpsC*	SSU ribosomal protein S3P	1.49	0.002846725	0.008646051
Pden_0778		conserved hypothetical protein	5.21	3.04E-06	6.87E-04
Pden_0779	*rplO*	LSU ribosomal protein L15P	2.50	1.47E-04	0.001416878
Pden_0787		N-acyl-L-homoserine lactone synthetase-like protein	-2.59	4.17E-04	0.002469493
Pden_0791		HAD-superfamily hydrolase, subfamily IA, variant 3	1.10	0.002647632	0.008218861

Pden_0793		L-aspartate-binding protein / L-glutamate-binding protein / L-glutamine-binding protein / L-asparagine-binding protein		-2.98	9.56E-05	0.001120928
Pden_0794		L-asparagine ABC transporter membrane protein / L-glutamine ABC transporter membrane protein / L-glutamate ABC transporter membrane protein / L-aspartate ABC transporter membrane protein		-2.92	8.85E-05	0.001071179
Pden_0795		L-glutamate ABC transporter membrane protein / L-asparagine ABC transporter membrane protein / L-glutamine ABC transporter membrane protein / L-aspartate ABC transporter membrane protein		-2.58	2.49E-04	0.001844661
Pden_0799		Citrate (pro-3S)-lyase		-2.00	6.52E-04	0.003231196
Pden_0801		binding-protein-dependent transport systems inner membrane component		-1.73	2.56E-04	0.001890373
Pden_0809		Lytic transglycosylase, catalytic		-1.13	0.002277759	0.00735009
Pden_0810		extracellular solute-binding protein, family 5		-2.75	9.39E-05	0.001108203
Pden_0821		hypothetical protein		1.75	1.03E-04	0.001157656
Pden_0823		protein of unknown function DUF404		-1.60	1.72E-04	0.001509512
Pden_0824		protein of unknown function DUF403		-1.54	2.07E-04	0.00167015
Pden_0829		extracellular solute-binding protein, family 1		-3.34	8.53E-06	6.87E-04
Pden_0830		binding-protein-dependent transport systems inner membrane component		-2.96	2.37E-04	0.001782039
Pden_0831		binding-protein-dependent transport systems inner membrane component		-2.60	4.09E-05	8.15E-04
Pden_0832		ABC transporter related protein		-1.96	1.30E-04	0.001314195
Pden_0841		hypothetical protein		-2.71	2.85E-04	0.00201284
Pden_0848		Malate dehydrogenase (oxaloacetate-decarboxylating) (NADP(+)), Phosphate acetyltransferase		-1.53	0.001491409	0.005527823
Pden_0851	*pncA*	Nicotinamidase		-2.44	7.56E-04	0.003539627
Pden_0852	*pncB*	nicotinate phosphoribosyltransferase		-2.65	6.40E-06	6.87E-04
Pden_0855	*gpt*	Xanthine phosphoribosyltransferase		-1.42	2.23E-04	0.00171547
Pden_0858	*pdxH*	Pyridoxamine 5'-phosphate oxidase		-1.96	1.06E-04	0.001171118
Pden_0864		ABC transporter related protein		3.66	1.81E-05	6.87E-04
Pden_0865		transport system permease protein		3.39	2.62E-04	0.001926306
Pden_0866		periplasmic binding protein		2.35	3.04E-05	7.09E-04
Pden_0867	*tsaA*	protein of unknown function UPF0066		2.48	1.21E-05	6.87E-04
Pden_0868		hypothetical protein		2.26	2.05E-05	6.87E-04
Pden_0869		conserved hypothetical protein		2.20	5.07E-05	9.00E-04
Pden_0872	*rpmH*	LSU ribosomal protein L34P		1.48	4.61E-04	0.002628348
Pden_0873	*rnpA*	ribonuclease P protein component		1.88	9.84E-05	0.001137092
Pden_0874	*yidD*	protein of unknown function DUF37		2.01	1.46E-04	0.00141608
Pden_0875	*ttcA*	PP-loop domain protein		1.79	0.001746111	0.006122271
Pden_0876		diguanylate phosphodiesterase		3.46	1.82E-05	6.87E-04
Pden_0877	*yidC*	protein translocase subunit yidC		1.14	0.001697137	0.006011301
Pden_0878		MOSC domain containing protein		2.39	5.21E-05	9.01E-04
Pden_0879	*engB*	small GTP-binding protein		2.59	7.64E-05	0.001002704
Pden_0886		hypothetical protein		3.14	0.001256538	0.004886217
Pden_0895	*prmC*	[protein release factor]-glutamine N5-methyltransferase		2.57	2.28E-04	0.001730572
Pden_0896		conserved hypothetical protein		1.57	2.71E-04	0.001970419
Pden_0909	*tatC*	Sec-independent protein translocase, TatC subunit		-1.04	0.002388697	0.007591949
Pden_0911		penicillin amidase, Cysteine peptidase, MEROPS family C59		-2.10	4.02E-04	0.00240555
Pden_0922	*prfC*	bacterial peptide chain release factor 3 (bRF-3)		1.95	0.001090882	0.004446969
Pden_0926		protein of unknown function DUF305		-2.82	0.001345336	0.005116549
Pden_0930		hypothetical protein		-2.50	1.68E-05	6.87E-04
Pden_0931		extracellular solute-binding protein, family 5		-1.39	0.001291564	0.004968989
Pden_0932		3-hydroxybutyrate dehydrogenase		-2.64	0.002190673	0.007174243
Pden_0935		Glyoxalase/bleomycin resistance protein/dioxygenase		-1.61	3.89E-04	0.002358731
Pden_0939	*aspA*	aspartate ammonia-lyase		-2.42	9.32E-05	0.001105227
Pden_0945		Substrate-binding region of ABC-type glycine betaine transport system		-1.84	0.001317477	0.005034736
Pden_0946		binding-protein-dependent transport systems inner membrane component		-1.92	5.26E-04	0.002843317
Pden_0947		glycine betaine/L-proline ABC transporter, ATPase subunit		-2.09	0.002115075	0.007017446
Pden_0954		cold-shock DNA-binding protein family		-2.38	0.00267388	0.008249759
Pden_0957	*phaZ*	polyhydroxyalkanoate depolymerase, intracellular		-3.80	0.001038143	0.00432914
Pden_0958	*phaC*	poly(R)-hydroxyalkanoic acid synthase, class I		-2.89	1.06E-05	6.87E-04
Pden_0959		conserved hypothetical protein		1.75	2.00E-04	0.001641009
Pden_0961		phage integrase family protein	2	-1.99	2.16E-04	0.001694513
Pden_0962		conserved hypothetical protein	2	-1.96	0.001364217	0.005166067
Pden_0970		DNA polymerase III beta subunit family protein	2	2.05	4.78E-04	0.002686491
Pden_0971		hypothetical protein	2	-1.32	0.001631432	0.005853961
Pden_0972		hypothetical protein	2	-1.44	3.38E-04	0.002227917
Pden_0973		hypothetical protein	2	-3.09	4.55E-04	0.0026076
Pden_0978		hypothetical protein	2	-3.84	4.73E-04	0.002669604
Pden_0983		phage/plasmid primase, P4 family	2	1.49	4.62E-04	0.002630851
Pden_0986		hypothetical protein	2	3.01	8.28E-04	0.003758078
Pden_0987		Resolvase helix-turn-helix domain protein	2	3.94	1.12E-05	6.87E-04
Pden_0988		phage Terminase	2	4.19	4.90E-06	6.87E-04
Pden_0989		phage portal protein, HK97 family	2	3.90	7.67E-06	6.87E-04
Pden_0990		peptidase S14, ClpP	2	2.54	6.41E-05	9.38E-04
Pden_0991		phage major capsid protein, HK97 family	2	1.88	3.45E-04	0.002238614
Pden_0993		hypothetical protein	2	1.75	0.002138223	0.007066463
Pden_0994		hypothetical protein	2	2.51	1.25E-04	0.001286187
Pden_0996		gene transfer agent	2	3.61	0.001052328	0.004364031
Pden_1003		conserved hypothetical protein		1.45	0.002372865	0.007565375
Pden_1004		hypothetical protein		2.55	5.89E-05	9.13E-04
Pden_1008	*pta*	phosphate acetyltransferase		2.20	0.001330838	0.005073821
Pden_1009		conserved hypothetical protein 698		2.08	0.00104852	0.004358501
Pden_1017		transposase IS116/IS110/IS902 family protein		-2.41	0.001209419	0.004754106
Pden_1020		transcriptional regulator, LysR family		-1.68	2.00E-04	0.001641009
Pden_1021		spermidine/putrescine ABC transporter, periplasmic binding protein, putative		-2.82	7.94E-04	0.003647678
Pden_1023		binding-protein-dependent transport systems inner membrane component		-2.13	0.00209069	0.006968507
Pden_1024		ABC transporter related protein		-2.23	0.001881956	0.006469312
Pden_1032		monosaccharide ABC transporter substrate-binding protein, CUT2 family		-2.34	6.28E-05	9.34E-04
Pden_1033		monosaccharide ABC transporter ATP-binding protein, CUT2 family		-2.40	5.59E-05	9.01E-04
Pden_1037		amidohydrolase		-1.18	0.001346088	0.005116549
Pden_1038		protein of unknown function DUF6, transmembrane		1.70	2.03E-04	0.001651967
Pden_1056		hypothetical protein		2.24	6.53E-05	9.42E-04
Pden_1057		N-isopropylammelide isopropylaminohydrolase		-2.68	3.48E-04	0.002243683

Pden_1062		flavin reductase domain protein, FMN-binding protein	-2.10	5.54E-05	9.01E-04
Pden_1077		carbohydrate ABC transporter ATP-binding protein, CUT1 family	3.19	2.38E-05	6.88E-04
Pden_1079		binding-protein-dependent transport systems inner membrane component	4.11	2.15E-05	6.88E-04
Pden_1080		beta-lactamase domain protein	4.17	1.62E-05	6.87E-04
Pden_1090		carbohydrate ABC transporter substrate-binding protein, CUT1 family	-1.58	0.002543101	0.007983175
Pden_1092		acetolactate synthase, large subunit	-3.06	1.79E-04	0.001533089
Pden_1094		conserved hypothetical protein	-2.24	1.67E-04	0.001505274
Pden_1100		major facilitator superfamily MFS_1	1.74	6.27E-04	0.003151095
Pden_1130		conserved hypothetical protein	4.42	5.90E-04	0.003052963
Pden_1131		conserved hypothetical protein	3.04	7.73E-04	0.003597541
Pden_1132		PRC-barrel containing protein	2.51	3.28E-04	0.002205051
Pden_1134		short-chain dehydrogenase/reductase SDR	1.00	0.002601094	0.008099224
Pden_1136		protein of unknown function DUF892	2.82	1.41E-05	6.87E-04
Pden_1138		transcriptional regulator, AraC family	-1.77	8.11E-04	0.003696149
Pden_1142		ABC transporter related protein	1.96	0.001053199	0.004364031
Pden_1143		ABC transporter related protein	1.53	0.003075685	0.009170902
Pden_1147		amino acid ABC transporter substrate-binding protein, PAAT family	-3.04	0.001056221	0.004364031
Pden_1148		amino acid ABC transporter membrane protein 1, PAAT family	-2.85	1.74E-04	0.0015113
Pden_1149		amino acid ABC transporter membrane protein 2, PAAT family	-3.08	6.84E-04	0.00331005
Pden_1150		amino acid ABC transporter ATP-binding protein, PAAT family	-2.29	3.38E-04	0.002227917
Pden_1151		agmatinase	-1.35	0.001218587	0.004786429
Pden_1157		periplasmic binding protein	2.38	9.45E-04	0.004070411
Pden_1158		conserved hypothetical protein	2.74	4.87E-05	8.89E-04
Pden_1161		D-isomer specific 2-hydroxyacid dehydrogenase, NAD-binding protein	1.36	0.002899917	0.008760311
Pden_1162		Arginase/agmatinase/formiminoglutamase	1.85	0.003217526	0.009468264
Pden_1163		NUDIX hydrolase	2.50	5.92E-04	0.00305499
Pden_1167		molybdenum ABC transporter, periplasmic molybdate-binding protein	1.75	1.61E-04	0.001491729
Pden_1168		periplasmic binding protein	1.73	8.57E-04	0.003825716
Pden_1169		transport system permease protein	2.27	0.003117722	0.009239266
Pden_1170		ABC transporter related protein	3.16	2.72E-04	0.001970419
Pden_1171		TonB-dependent receptor	1.56	7.92E-04	0.003644887
Pden_1172		TOBE domain protein	-3.54	2.77E-06	6.87E-04
Pden_1176	*catA*	catechol 1,2-dioxygenase	-4.06	0.001685714	0.005980919
Pden_1186	*fdhA*	glutathione-independent formaldehyde dehydrogenase	-3.03	7.40E-05	9.86E-04
Pden_1188		ferredoxin	-1.53	0.001591569	0.005776426
Pden_1193		glycine betaine/L-proline ABC transporter, ATPase subunit	-2.15	0.002400089	0.007623371
Pden_1194		binding-protein-dependent transport systems inner membrane component	-1.92	5.26E-04	0.00284302
Pden_1195		Substrate-binding region of ABC-type glycine betaine transport system	-1.84	0.001318043	0.005034736
Pden_1197	*hpbA*	NADH:flavin oxidoreductase/NADH oxidase	-1.16	0.002429284	0.007696786
Pden_1199		amidohydrolase	-3.22	8.18E-05	0.0010329
Pden_1200		conserved hypothetical protein	-2.62	1.12E-04	0.001193485
Pden_1201		conserved hypothetical protein	-1.78	0.00110413	0.004476884
Pden_1204	*ddl*	D-alanine--D-alanine ligase	-2.16	9.81E-05	0.001136331
Pden_1212	*ureD*	Urease accessory protein UreD	2.33	0.002026337	0.006816224
Pden_1214		transcriptional regulator, ArsR family	-1.84	0.001311362	0.005018463
Pden_1215		Activator of Hsp90 ATPase 1 family protein	-2.13	2.51E-04	0.001854347
Pden_1221		transposase, IS4 family	1.49	8.97E-04	0.003925429
Pden_1222		transposase-like protein	1.46	0.001716465	0.006050604
Pden_1223		phage integrase family protein	1.30	0.002793323	0.00852433
Pden_1225		hypothetical protein	-1.94	2.82E-04	0.002004634
Pden_1229		hypothetical protein	-2.20	0.002831053	0.00860878
Pden_1247		glutathionylspermidine synthase	-1.41	0.001969504	0.006683376
Pden_1248		extracellular solute-binding protein, family 5	-2.13	1.30E-04	0.001314195
Pden_1250		binding-protein-dependent transport systems inner membrane component	-1.49	0.003114626	0.009239266
Pden_1252		ABC transporter related protein	-1.30	0.002714476	0.008329327
Pden_1255		short-chain dehydrogenase/reductase SDR	-2.91	1.69E-04	0.00150607
Pden_1256		ABC-3 protein	-2.27	3.61E-04	0.002294387
Pden_1257		ABC-3 protein	-2.60	5.86E-05	9.13E-04
Pden_1258		ABC transporter related protein	-2.70	7.58E-05	0.001002205
Pden_1259		periplasmic solute binding protein	-3.57	4.80E-05	8.86E-04
Pden_1265	*clpP*	ATP-dependent Clp protease proteolytic subunit ClpP	-1.98	0.002994665	0.008987544
Pden_1271	*accC*	acetyl-CoA carboxylase carboxyltransferase subunit alpha	-1.35	0.003420113	0.009889224
Pden_1277		uracil-xanthine permease	-3.56	1.76E-05	6.87E-04
Pden_1284		multisubunit potassium/proton antiporter, PhaG subunit	-1.05	0.002299629	0.007387705
Pden_1297	*fcl*	NAD-dependent epimerase/dehydratase	3.21	1.23E-04	0.001274131
Pden_1298	*gmd*	GDP-mannose 4,6-dehydratase	2.65	7.86E-06	6.87E-04
Pden_1305		Antibiotic biosynthesis monooxygenase	-3.32	2.36E-05	6.88E-04
Pden_1306		Cobyrinic acid a,c-diamide synthase	-1.87	7.19E-05	9.81E-04
Pden_1308	*uvrB*	Excinuclease ABC subunit B	-1.66	0.003140266	0.009292324
Pden_1315	*mdoH*	glycosyl transferase, family 2	1.08	0.002205847	0.007214602
Pden_1316		conserved hypothetical protein	1.84	6.88E-04	0.00331005
Pden_1322		conserved hypothetical protein	1.89	0.002798302	0.008529684
Pden_1323		pyridoxamine 5'-phosphate oxidase-related, FMN-binding protein	-1.69	1.07E-04	0.001176884
Pden_1324		oligopeptide/dipeptide ABC transporter, ATPase subunit	-1.44	5.67E-04	0.00297487
Pden_1325		oligopeptide/dipeptide ABC transporter, ATPase subunit	-1.39	0.001270728	0.004922492
Pden_1326		binding-protein-dependent transport systems inner membrane component	-2.17	5.12E-05	9.00E-04
Pden_1327	*oppB*	binding-protein-dependent transport systems inner membrane component	-3.49	4.86E-06	6.87E-04
Pden_1328		extracellular solute-binding protein, family 5	-3.91	3.31E-04	0.002212274
Pden_1341		extracellular solute-binding protein, family 5	3.58	8.17E-06	6.87E-04
Pden_1342		binding-protein-dependent transport systems inner membrane component	3.80	5.01E-05	9.00E-04
Pden_1343		binding-protein-dependent transport systems inner membrane component	3.94	2.14E-04	0.001694513
Pden_1344		ABC transporter related protein	2.55	8.10E-04	0.003693153
Pden_1346	*prpB*	2,3-dimethylmalate lyase	-2.44	6.20E-04	0.003143656
Pden_1347	*prpC*	2-methylcitrate synthase	-2.51	7.36E-04	0.003470891
Pden_1348		2-methylcitrate dehydratase	-1.92	6.84E-04	0.00331005
Pden_1355		methionine synthase (B12-independent)	-2.83	8.05E-04	0.003682344
Pden_1362		periplasmic binding protein	-1.29	0.001406434	0.00527856

Pden_1372		phosphoesterase or phosphohydrolase-like protein	2.33	0.002098161	0.006983413
Pden_1373		ATP-dependent Lon protease	3.35	1.22E-04	0.00126941
Pden_1374		metallophosphoesterase	2.71	1.70E-05	6.87E-04
Pden_1376	*lipA*	lipoic acid synthetase	-2.26	0.001169221	0.004646598
Pden_1377		Redoxin domain protein	3.81	7.26E-05	9.81E-04
Pden_1380		protein of unknown function DUF853, NPT hydrolase putative	-1.84	6.97E-04	0.003346308
Pden_1382		protease Do	-2.17	0.001038891	0.00432914
Pden_1383		hypothetical protein	-2.01	9.95E-04	0.004206003
Pden_1384		ribonucleoside-diphosphate reductase class II	-2.40	2.77E-04	0.001988805
Pden_1395		aminotransferase, class V	-1.54	0.001962537	0.006668949
Pden_1397		L-glutamine synthetase	-1.93	0.001756559	0.006137651
Pden_1420	*dusA*	tRNA-U16,U17-dihydrouridine synthase	1.51	8.10E-04	0.003693153
Pden_1427		TonB-dependent receptor	1.80	5.12E-04	0.002788261
Pden_1428		transcriptional regulator, AraC family	1.82	0.002019802	0.006808426
Pden_1431		transcriptional regulator, GntR family	-2.60	5.11E-05	9.00E-04
Pden_1432		BioY protein	-1.96	4.40E-04	0.00254936
Pden_1433	*bioB*	biotin synthase	-2.55	1.95E-05	6.87E-04
Pden_1436		MOSC domain containing protein	-1.32	0.002270739	0.007336792
Pden_1443		conserved hypothetical protein	-1.89	0.001166275	0.004645832
Pden_1449		conserved hypothetical protein	1.60	0.001855333	0.00641198
Pden_1455		major facilitator superfamily MFS_1	-3.15	7.83E-04	0.003626579
Pden_1461		GCN5-related N-acetyltransferase	-2.45	3.97E-04	0.002393936
Pden_1463		transcriptional regulator, LysR family	-2.43	0.00187387	0.00645841
Pden_1466		helix-turn-helix- domain containing protein, AraC type	-2.49	3.15E-05	7.09E-04
Pden_1468		protein of unknown function DUF1234	-1.44	8.09E-04	0.003693153
Pden_1482		conjugal transfer protein trbB	2.29	8.65E-04	0.003825889
Pden_1492		ABC transporter related protein	-1.84	9.86E-05	0.001137092
Pden_1496		major facilitator superfamily MFS_1	1.47	0.001153375	0.00461259
Pden_1497		transcriptional regulator, AraC family	-5.24	2.40E-05	6.88E-04
Pden_1506		TRAG family protein	1.70	6.15E-04	0.003131842
Pden_1510		conjugal transfer protein precursor	2.68	3.69E-04	0.002309828
Pden_1512		replication protein A	2.45	0.003284787	0.009635503
Pden_1518		protein of unknown function DUF736	3.96	6.85E-04	0.00331005
Pden_1519		hypothetical protein	3.17	4.42E-04	0.00255247
Pden_1525		hypothetical protein	-2.61	0.000362817	0.002299054
Pden_1526		helicase domain protein	-2.49	3.08E-05	7.09E-04
Pden_1527		DEAD/DEAH box helicase domain protein	-2.53	5.88E-05	9.13E-04
Pden_1528		protein of unknown function DUF262	-2.80	5.62E-04	0.002961881
Pden_1529		conserved hypothetical protein	-1.69	9.26E-05	0.001100653
Pden_1530		hypothetical protein	-1.92	5.66E-04	0.00297487
Pden_1534		filamentation induced by cAMP protein Fic	-1.85	0.002048127	0.006871879
Pden_1541		ParB family protein	3.04	1.04E-04	0.001161644
Pden_1543		conserved hypothetical protein	4.77	4.89E-04	0.002721641
Pden_1544		domain of unknown function DUF1738	4.07	6.01E-05	9.25E-04
Pden_1545		histone family protein nucleoid-structuring protein H-NS	-3.50	4.56E-06	6.87E-04
Pden_1546		ORF-3 family protein	-3.65	7.96E-05	0.001019694
Pden_1548		HipA domain protein	-1.31	6.05E-04	0.003092881
Pden_1551		protein of unknown function DUF481	-2.31	6.50E-04	0.003223566
Pden_1558		Capsule polysaccharide biosynthesis	2.58	0.001042715	0.0043415
Pden_1559		polysaccharide export protein	-2.07	0.001004826	0.004225445
Pden_1562	*nrdR*	ATP-cone domain protein	-1.69	0.001697839	0.006011301
Pden_1563		TRAP dicarboxylate transporter- DctP subunit	-4.17	3.03E-05	7.09E-04
Pden_1564		TRAP dicarboxylate transporter, DctM subunit	-1.33	6.99E-04	0.003354223
Pden_1569		extracellular solute-binding protein, family 5	-3.90	2.99E-04	0.002061667
Pden_1570	*oppB*	binding-protein-dependent transport systems inner membrane component	-3.49	4.85E-06	6.87E-04
Pden_1571		binding-protein-dependent transport systems inner membrane component	-2.17	5.12E-05	9.00E-04
Pden_1572		oligopeptide/dipeptide ABC transporter, ATPase subunit	-1.39	0.001269456	0.004921331
Pden_1573		oligopeptide/dipeptide ABC transporter, ATPase subunit	-1.44	5.67E-04	0.00297487
Pden_1574		pyridoxamine 5'-phosphate oxidase-related, FMN-binding protein	-1.69	1.08E-04	0.001177715
Pden_1575		conserved hypothetical protein	1.91	0.002949957	0.008874411
Pden_1581		conserved hypothetical protein	1.84	6.87E-04	0.00331005
Pden_1582	*mdoH*	glycosyl transferase, family 2	1.10	0.001950252	0.00664103
Pden_1584		hypothetical protein	2.61	0.002117893	0.007022197
Pden_1590		plasmid segregation oscillating ATPase ParF	-1.87	7.18E-05	9.81E-04
Pden_1591		Antibiotic biosynthesis monooxygenase	-3.32	2.36E-05	6.88E-04
Pden_1595		ABC transporter related protein	2.91	6.19E-05	9.33E-04
Pden_1596		ABC-3 protein	2.43	9.65E-04	0.004115048
Pden_1603		Dihydrolipoyllysine-residue succinyltransferase	1.44	0.002794862	0.00852433
Pden_1604		Transketolase, central region	1.07	0.003454511	0.00996592
Pden_1605		flavoprotein	-1.29	0.002705269	0.008311152
Pden_1610		binding-protein-dependent transport systems inner membrane component	2.68	8.60E-04	0.003825716
Pden_1611		ABC transporter related protein	2.52	0.001650172	0.005905195
Pden_1620		flagellin domain protein	-3.35	0.002125117	0.007041535
Pden_1624		transposase IS3/IS911 family protein	2.02	0.001612076	0.005813604
Pden_1629		RNA polymerase, sigma-24 subunit, ECF subfamily	2.68	3.47E-05	7.44E-04
Pden_1630		putative FecR	1.91	9.90E-05	0.001137092
Pden_1631		TonB-dependent receptor, plug	2.84	2.54E-05	7.00E-04
Pden_1632		hypothetical protein	3.45	0.001625795	0.005840986
Pden_1633		uncharacterized enzyme	2.28	3.17E-04	0.002148938
Pden_1636		isochorismatase hydrolase	2.98	6.20E-04	0.003143656
Pden_1640	*pta*	phosphotransacetylase	2.16	0.001423591	0.005327157
Pden_1641		conserved hypothetical protein 698	2.14	9.48E-04	0.004070411
Pden_1653		lytic murein transglycosylase	-1.93	3.25E-04	0.002189096
Pden_1657	*panB*	ketopantoate hydroxymethyltransferase	-1.02	0.001822465	0.006319958
Pden_1662		Hemolysin-type calcium-binding region	-2.34	3.71E-04	0.002309828
Pden_1666		L-lysine 2,3-aminomutase	1.70	1.16E-04	0.001218765
Pden_1667		ABC transporter related protein	-1.77	1.73E-04	0.001509512

Pden_1668		ABC transporter related protein	-2.04	0.001140538	0.004578915
Pden_1669		binding-protein-dependent transport systems inner membrane component	-2.37	4.90E-04	0.002725905
Pden_1670		binding-protein-dependent transport systems inner membrane component	-2.85	2.55E-05	7.00E-04
Pden_1671		extracellular solute-binding protein, family 5	-5.62	2.64E-04	0.00192913
Pden_1672	*iolB*	5-deoxyglucuronate isomerase	-1.84	1.60E-04	0.001489115
Pden_1673		conserved hypothetical protein	-2.15	7.15E-04	0.00340186
Pden_1674	*iolE*	2-keto-myo-inositol dehydratase	-2.14	6.58E-04	0.003245718
Pden_1675	*iolD*	3D-(3,5/4)-trihydroxycyclohexane-1,2-dione hydrolase	-1.79	0.0020993	0.006983413
Pden_1678	*iolG*	myo-inositol 2-dehydrogenase	-2.36	2.62E-04	0.001926306
Pden_1680		oxidoreductase domain protein	-2.35	2.48E-04	0.00184162
Pden_1681		monosaccharide ABC transporter substrate-binding protein, CUT2 family	-3.76	2.97E-04	0.002051463
Pden_1682		monosaccharide ABC transporter membrane protein, CUT2 family	-3.81	2.05E-05	6.87E-04
Pden_1683	*rbsA*	monosaccharide ABC transporter ATP-binding protein, CUT2 family	-4.05	1.11E-04	0.00118365
Pden_1684		monosaccharide ABC transporter substrate-binding protein, CUT2 family	-3.72	4.62E-05	8.76E-04
Pden_1688		conserved hypothetical protein	1.77	0.001555936	0.005687871
Pden_1692		creatinase	-1.51	0.003159321	0.009336478
Pden_1693		major facilitator superfamily MFS_1	-3.27	2.27E-04	0.001730572
Pden_1695	*fbp*	D-fructose 1,6-bisphosphatase	-2.99	9.36E-04	0.004043846
Pden_1697	*tkt*	transketolase	-2.31	3.27E-04	0.002198565
Pden_1698		fructose-bisphosphate aldolase	-3.10	9.30E-04	0.004030546
Pden_1699	*cbbL*	ribulose-1,5-bisphosphate carboxylase/oxygenase large subunit	-3.44	2.64E-04	0.001931095
Pden_1700		ribulose 1,5-bisphosphate carboxylase small subunit	-3.08	0.002722227	0.008342998
Pden_1701	*cbbX*	AAA ATPase, central domain protein	-1.45	0.001283293	0.004945029
Pden_1710		Betaine-aldehyde dehydrogenase	-5.98	0.002896496	0.008755198
Pden_1712		glucose-methanol-choline oxidoreductase	-2.60	6.97E-05	9.69E-04
Pden_1713		amino acid/amide ABC transporter membrane protein 2, HAAT family	-3.31	1.52E-05	6.87E-04
Pden_1714		amino acid/amide ABC transporter membrane protein 1, HAAT family	-3.38	4.65E-05	8.76E-04
Pden_1715		amino acid/amide ABC transporter ATP-binding protein 2, HAAT family	-2.92	1.07E-04	0.001176884
Pden_1716		amino acid/amide ABC transporter ATP-binding protein 1, HAAT family	-3.33	2.48E-05	6.90E-04
Pden_1717		amino acid/amide ABC transporter substrate-binding protein, HAAT family	-3.16	3.47E-05	7.44E-04
Pden_1718		transcriptional regulator, LysR family	-1.56	2.02E-04	0.001645714
Pden_1719		AMP-dependent synthetase and ligase	-2.67	6.47E-06	6.87E-04
Pden_1721		transporter, UIT1 family	-1.92	3.71E-04	0.002309828
Pden_1725		hypothetical protein	-2.14	2.25E-04	0.001721674
Pden_1727		conserved hypothetical protein	-2.25	2.87E-04	0.002014555
Pden_1734		protein of unknown function DUF451	-2.77	5.29E-06	6.87E-04
Pden_1735	*efeB*	Dyp-type peroxidase family	-3.50	1.82E-06	6.87E-04
Pden_1736	*efeO*	protein of unknown function DUF451	-5.52	1.62E-06	6.87E-04
Pden_1741		conserved hypothetical protein	2.39	8.61E-04	0.003825716
Pden_1742	*dtd*	D-tyrosyl-tRNA(Tyr) deacylase	2.29	3.32E-04	0.002212274
Pden_1744	*tdk*	thymidine kinase	2.28	1.96E-04	0.001631407
Pden_1747		ABC transporter related protein	2.15	4.47E-04	0.002574525
Pden_1753		conserved hypothetical protein	-1.70	0.001402322	0.005267032
Pden_1754		short-chain dehydrogenase/reductase SDR	1.42	9.58E-04	0.004097585
Pden_1757		Serine-type D-Ala-D-Ala carboxypeptidase	-1.82	2.01E-04	0.0016447
Pden_1761		protein of unknown function DUF6, transmembrane	1.87	1.02E-04	0.00115165
Pden_1774		periplasmic binding protein	-1.58	0.00278277	0.008507948
Pden_1778		drug resistance transporter, Bcr/CflA subfamily	1.08	0.002235753	0.007265479
Pden_1780		efflux transporter, RND family, MFP subunit	-1.86	7.89E-05	0.001019694
Pden_1781		transporter, hydrophobe/amphiphile efflux-1 (HAE1) family	-1.74	8.72E-05	0.001065159
Pden_1783		signal transduction histidine kinase, glucose-6-phosphate specific	-2.15	4.51E-04	0.00259101
Pden_1784		two component transcriptional regulator, LuxR family	-2.47	5.61E-05	9.01E-04
Pden_1785		helix-turn-helix domain protein	-1.91	0.001365353	0.005166504
Pden_1791	*maf*	maf protein	1.15	8.89E-04	0.00389767
Pden_1792		ribonuclease	2.14	2.05E-05	6.87E-04
Pden_1793	*yacG*	protein of unknown function DUF329	2.53	3.08E-05	7.09E-04
Pden_1795		Allergen V5/Tpx-1 family protein	-1.66	3.75E-04	0.002309828
Pden_1798		sulfate ABC transporter, periplasmic sulfate-binding protein	-1.36	0.002309806	0.007415694
Pden_1807		extracellular solute-binding protein, family 5	-1.83	5.90E-04	0.003052963
Pden_1810		conserved hypothetical protein	1.86	0.003048021	0.009099135
Pden_1811		5'-Nucleotidase domain protein	1.55	0.001003093	0.004221661
Pden_1814		Patatin	3.93	7.08E-05	9.77E-04
Pden_1815	*prfB*	bacterial peptide chain release factor 2 (bRF-2)	1.69	1.07E-04	0.001177715
Pden_1818		aminotransferase	1.27	0.002871511	0.008705666
Pden_1823		peptidase M20	-1.97	5.03E-04	0.002762958
Pden_1830		Sterol-binding domain protein	-1.63	0.001241705	0.004844257
Pden_1831		alpha/beta hydrolase fold protein	-1.40	0.002889802	0.008745409
Pden_1832		oligoendopeptidase, pepF/M3 family	-1.15	0.003346613	0.00974908
Pden_1834		Glutathione S-transferase, C-terminal domain	-2.73	7.42E-04	0.003490386
Pden_1838		phosphoesterase, PA-phosphatase related protein	2.42	0.003369582	0.009799076
Pden_1839	*lolD*	ABC transporter related protein	1.88	0.001106291	0.004479442
Pden_1840		lipoprotein releasing system, transmembrane protein, LolC/E family	1.25	0.003182421	0.0093951
Pden_1845	*ccoP*	cytochrome c oxidase, cbb3-type, subunit III	-1.98	7.83E-04	0.003626579
Pden_1846		Cbb3-type cytochrome oxidase component	-1.99	0.001179375	0.004668627
Pden_1847	*ccoO*	cytochrome c oxidase, cbb3-type, subunit II	-1.59	0.002570443	0.00802847
Pden_1856		histidine triad (HIT) protein	-1.95	3.12E-04	0.002120675
Pden_1860	*plsX*	phosphate:acyl-[acyl carrier protein] acyltransferase	1.72	6.59E-04	0.003246886
Pden_1861	*fabH*	3-oxoacyl-[acyl-carrier-protein] synthase III	2.45	1.58E-04	0.00147988
Pden_1862		transcriptional regulator, TraR/DksA family	1.80	8.89E-04	0.00389767
Pden_1863	*ihfA*	integration host factor, alpha subunit	-1.64	2.15E-04	0.001694513
Pden_1879	*carB*	carbamoyl-phosphate synthase large subunit	2.10	8.29E-05	0.00104193
Pden_1884	*hisH*	imidazole glycerol phosphate synthase subunit hisH	1.39	0.00105658	0.004364031
Pden_1885		conserved hypothetical protein	-3.07	0.001611899	0.005813604
Pden_1886	*hisA*	1-(5-phosphoribosyl)-5-[(5-phosphoribosylamino)methylideneamino] imidazole-4-carboxamide isomerase	2.01	0.003118551	0.009239266
Pden_1889		MgtC/SapB transporter	1.36	0.002011227	0.006788563
Pden_1898	*betI*	transcriptional regulator, TetR family	1.58	6.41E-04	0.003194596

Pden_1899	*choX*	Substrate-binding region of ABC-type glycine betaine transport system	-1.93	3.99E-04	0.002394246
Pden_1900	*choW*	binding-protein-dependent transport systems inner membrane component	-2.59	1.36E-04	0.001338383
Pden_1901	*choV*	ABC transporter related protein	-2.73	2.82E-05	7.03E-04
Pden_1903		Lysine exporter protein (LYSE/YGGA)	3.44	4.21E-04	0.002481719
Pden_1919		hypothetical protein	2.05	0.001430053	0.005343439
Pden_1920		fructose-bisphosphate aldolase	-1.62	1.73E-04	0.001509649
Pden_1922		hypothetical protein	2.34	2.07E-05	6.87E-04
Pden_1923		ATP phosphoribosyltransferase catalytic subunit	2.58	3.40E-04	0.002230025
Pden_1924		ATP phosphoribosyltransferase regulatory subunit	2.13	1.05E-04	0.001166968
Pden_1925	*hisS*	histidyl-tRNA synthetase	1.46	9.30E-04	0.004030546
Pden_1933		O-acetylhomoserine sulfhydrolase	-2.50	7.65E-05	0.001002704
Pden_1934		translation initiation factor 2, gamma subunit, GTPase	4.04	2.24E-04	0.00171547
Pden_1937		cytochrome c, class I	-2.86	4.13E-04	0.002451367
Pden_1938	*ctaD*	Cytochrome-c oxidase	-2.66	0.001533422	0.005621808
Pden_1939		conserved hypothetical protein	-1.20	0.001840961	0.006366658
Pden_1940		FAD dependent oxidoreductase	1.15	0.002219728	0.007231971
Pden_1941		Glyoxylate reductase	-1.47	4.19E-04	0.002476529
Pden_1945	*moeB*	UBA/THIF-type NAD/FAD binding protein	2.99	1.77E-04	0.001524591
Pden_1946	*dut*	deoxyuridine 5'-triphosphate nucleotidohydrolase	1.10	0.001448421	0.005395971
Pden_1948		conserved hypothetical protein	-1.21	0.002259698	0.00731094
Pden_1949		putative L-sorbosone dehydrogenase	-1.63	3.64E-04	0.002303161
Pden_1953		conserved hypothetical protein	2.92	8.52E-04	0.003818535
Pden_1954		SAM-dependent methyltransferase	2.31	1.99E-04	0.001641009
Pden_1955	*edd*	6-phosphogluconate dehydratase	2.31	0.001862483	0.00642561
Pden_1956		putative PAS/PAC sensor protein	2.92	1.83E-05	6.87E-04
Pden_1962		hypothetical protein	3.98	0.001388292	0.005229852
Pden_1973		hypothetical protein	1.95	2.74E-04	0.001979409
Pden_1974		Mu-like prophage major head subunit gpT	7.10	0.001989825	0.006743296
Pden_1975		hypothetical protein	4.80	2.71E-06	6.87E-04
Pden_1984	*ilvD*	dihydroxyacid dehydratase	-1.46	9.67E-04	0.00411907
Pden_1985		pterin-4-alpha-carbinolamine dehydratase	-2.38	1.02E-04	0.00115165
Pden_1986	*cls*	cardiolipin synthetase 2	-1.95	5.79E-05	9.13E-04
Pden_1991		aldehyde dehydrogenase	-1.67	2.75E-04	0.001980264
Pden_1993		hypothetical protein	4.03	5.58E-05	9.01E-04
Pden_1994		hypothetical protein	1.60	9.72E-04	0.004133467
Pden_2001		protein of unknown function DUF55	-1.40	6.27E-04	0.003151095
Pden_2004	*lysM*	Peptidoglycan-binding LysM	-2.43	5.20E-04	0.002814686
Pden_2005		AMP-dependent synthetase and ligase	-2.94	1.46E-05	6.87E-04
Pden_2013		ABC-2 type transporter	-1.40	3.93E-04	0.002373286
Pden_2020	*lysA*	diaminopimelate decarboxylase	1.05	0.001278181	0.004938294
Pden_2025		diguanylate phosphodiesterase	1.23	0.001141337	0.004578915
Pden_2026		acetyl-CoA acetyltransferase	-2.78	5.36E-05	9.01E-04
Pden_2027		3-oxoacyl-[acyl-carrier-protein] reductase	-3.70	8.04E-04	0.003682344
Pden_2028		protein of unknown function DUF465	-4.80	0.001626662	0.005840986
Pden_2034		toluene tolerance family protein	-2.59	1.36E-05	6.87E-04
Pden_2039	*argF*	ornithine carbamoyltransferase	-1.30	8.73E-04	0.003853738
Pden_2040		GCN5-related N-acetyltransferase	2.18	1.61E-04	0.001492398
Pden_2041	*argD*	acetylornithine aminotransferase apoenzyme	1.96	6.26E-05	9.34E-04
Pden_2046		TonB-dependent receptor	-1.82	1.35E-04	0.001332887
Pden_2047	*rocF*	arginase	-2.53	2.60E-05	7.00E-04
Pden_2052		hypothetical protein	-2.37	3.73E-04	0.002309828
Pden_2053		hypothetical protein	-2.16	7.70E-05	0.001002704
Pden_2056		hypothetical protein	2.06	5.32E-04	0.002866564
Pden_2063		conserved hypothetical phage terminase large subunit	2.78	6.22E-04	0.003147257
Pden_2064		phage terminase, small subunit, putative, P27 family	3.23	6.96E-05	9.69E-04
Pden_2068		ParB domain protein nuclease	3.64	8.34E-06	6.87E-04
Pden_2072		hypothetical protein	3.21	8.73E-05	0.001065159
Pden_2076		RNA polymerase, sigma-24 subunit, ECF subfamily	1.95	0.003126823	0.009257955
Pden_2078		hypothetical protein	-2.77	4.22E-04	0.002481719
Pden_2082		conserved hypothetical protein	2.08	0.001950467	0.00664103
Pden_2085		conserved hypothetical protein	-1.65	0.003406586	0.009867016
Pden_2087		conserved hypothetical protein	-1.45	6.76E-04	0.003305876
Pden_2088		endonuclease	-2.40	7.56E-04	0.0035394
Pden_2089		conserved hypothetical protein	-1.89	2.31E-04	0.001743752
Pden_2090		conserved hypothetical protein	-2.39	0.001277162	0.004938294
Pden_2098		alkane 1-monooxygenase	1.46	7.84E-04	0.003626579
Pden_2101		DNA methylase N-4/N-6 domain protein	-2.02	2.09E-04	0.001676926
Pden_2125		ABC-2 type transporter	1.48	0.003015167	0.009033013
Pden_2127		ABC transporter related protein	1.67	0.001241921	0.004844257
Pden_2130		Nitrilase/cyanide hydratase and apolipoprotein N-acyltransferase	2.03	0.001603336	0.005802495
Pden_2131		GCN5-related N-acetyltransferase	2.56	1.35E-04	0.001332887
Pden_2143		Serine-type D-Ala-D-Ala carboxypeptidase	-1.59	3.67E-04	0.002309564
Pden_2149		binding-protein-dependent transport systems inner membrane component	-2.58	2.80E-04	0.001999931
Pden_2150		binding-protein-dependent transport systems inner membrane component	-2.55	2.91E-04	0.002029518
Pden_2151		polyamine ABC trasnporter, periplasmic polyamine-binding protein	-2.19	0.00188353	0.006469312
Pden_2152		extracellular solute-binding protein, family 1	-2.59	6.46E-04	0.003213642
Pden_2164		GatB/Yqey domain protein	1.18	9.93E-04	0.004204018
Pden_2166		General secretory system II, protein E domain protein	2.91	6.16E-05	9.33E-04
Pden_2172		peptidase M48, Ste24p	-1.56	2.86E-04	0.002014555
Pden_2173		conserved hypothetical protein	-1.89	1.22E-04	0.00126941
Pden_2182		conserved hypothetical protein	1.54	7.64E-04	0.003564054
Pden_2189	*cobS*	cobaltochelatase CobS subunit	-1.40	0.001573969	0.005733096
Pden_2190		hypothetical protein	-1.00	0.002184219	0.007167014
Pden_2193		peptidase M24	-1.31	3.36E-04	0.002227917
Pden_2199		conserved hypothetical protein	-1.94	8.57E-04	0.003825716
Pden_2204		conserved hypothetical protein	-1.73	8.98E-05	0.001079381
Pden_2219	*upp*	uracil phosphoribosyltransferase	-1.02	0.003090137	0.009203155

Pden_2224		Malate dehydrogenase (oxaloacetate-decarboxylating) (NADP(+))., Phosphate acetyltransferase	-2.11	1.59E-04	0.001488419
Pden_2225		propionyl-CoA synthetase	-3.05	5.73E-05	9.13E-04
Pden_2226	*aroA*	3-phosphoshikimate 1-carboxyvinyltransferase	1.47	0.001167673	0.004646598
Pden_2247	*nuoD (nqo4)*	NADH dehydrogenase subunit D	-1.13	0.002132296	0.00705609
Pden_2248	*nuoC*	NADH dehydrogenase subunit C	-1.25	6.35E-04	0.0031761
Pden_2249	*nuoB (nqo6)*	NADH dehydrogenase subunit B	-1.05	0.001830636	0.00634395
Pden_2253		glutathione-dependent formaldehyde-activating, GFA	-2.21	8.59E-05	0.001060694
Pden_2255		conserved hypothetical protein	-2.30	2.13E-05	6.88E-04
Pden_2257		succinate semialdehyde dehydrogenase	-3.27	1.04E-05	6.87E-04
Pden_2262	*pheT*	phenylalanyl-tRNA synthetase beta subunit	1.38	8.35E-04	0.003775583
Pden_2265	*mscL*	large conductance mechanosensitive channel protein	-3.22	0.003307025	0.009695118
Pden_2272		cytochrome P450	3.42	2.42E-05	6.88E-04
Pden_2275		hypothetical protein	1.09	0.003333896	0.009734438
Pden_2276	*pyk*	pyruvate kinase	1.37	6.55E-04	0.003235458
Pden_2278		hypothetical protein	-1.78	1.60E-04	0.001489115
Pden_2280		hypothetical protein	-1.46	4.40E-04	0.00254936
Pden_2281		Arc domain protein DNA binding domain protein	-2.45	0.001428881	0.005343002
Pden_2285	*choX*	Substrate-binding region of ABC-type glycine betaine transport system	-3.69	6.47E-05	9.41E-04
Pden_2286	*betC*	sulfatase	-1.58	1.49E-04	0.001431346
Pden_2287		membrane protein of uknown function UCP014873	1.60	0.002009075	0.006788563
Pden_2291		protein of unknown function DUF6, transmembrane	1.27	0.002463045	0.007779412
Pden_2292		conserved hypothetical protein	1.73	0.001103082	0.004476884
Pden_2293		glycosyl transferase, family 14	3.47	4.23E-06	6.87E-04
Pden_2294		conserved hypothetical protein	1.98	1.08E-04	0.00118008
Pden_2297		UDP-glucose pyrophosphorylase	-2.09	8.12E-05	0.00102897
Pden_2302	*dnaK*	chaperone protein DnaK	-2.09	0.00205743	0.006885889
Pden_2313		conserved hypothetical protein	-2.14	0.003336044	0.009735094
Pden_2314		conserved hypothetical protein	-1.85	1.92E-04	0.001609815
Pden_2327		protein of unknown function DUF1428	-2.86	5.16E-04	0.002802086
Pden_2328		Glyoxalase/bleomycin resistance protein/dioxygenase	-2.38	4.07E-05	8.15E-04
Pden_2332		cobalamin (vitamin B12) biosynthesis CbiX protein	-1.66	5.85E-04	0.003035437
Pden_2333		conserved hypothetical protein	-2.21	7.89E-04	0.003638028
Pden_2338		4-aminobutyrate aminotransferase apoenzyme	-3.58	3.32E-05	7.22E-04
Pden_2349		short-chain dehydrogenase/reductase SDR	-1.87	3.15E-04	0.002139228
Pden_2351		conserved hypothetical protein	-1.25	0.001140209	0.004578915
Pden_2353		domain of unknown function DUF1745	-2.12	0.002381669	0.007583468
Pden_2354		two component transcriptional regulator, LuxR family	-3.04	3.36E-04	0.002227917
Pden_2359	*pqqE*	coenzyme PQQ biosynthesis protein E	-1.81	0.00187867	0.006466844
Pden_2360	*pqqD*	coenzyme PQQ synthesis D	-2.22	1.71E-04	0.001509512
Pden_2361	*pqqC*	coenzyme PQQ biosynthesis protein C	-2.06	2.16E-04	0.001694513
Pden_2362	*pqqB*	coenzyme PQQ biosynthesis protein B	-2.40	3.41E-04	0.002237339
Pden_2366	*adh*	Aldehyde dehydrogenase (NAD(+))	-5.40	4.86E-04	0.0027113
Pden_2367	*adhP*	Alcohol dehydrogenase GroES domain protein	-4.03	8.70E-05	0.001065159
Pden_2368		protein of unknown function DUF779	-4.01	6.80E-04	0.003306024
Pden_2374		two component transcriptional regulator, winged helix family	-1.90	0.002277333	0.00735009
Pden_2378		putative RNA methylase	1.30	6.79E-04	0.003306024
Pden_2380		metallophosphoesterase	1.56	2.70E-04	0.001969565
Pden_2386		2,3-dihydroxybenzoate-AMP ligase	-2.19	1.53E-04	0.001451477
Pden_2393	*gndA*	6-phosphogluconate dehydrogenase (decarboxylating)	2.28	0.001594182	0.005776476
Pden_2394		hypothetical protein	4.86	7.55E-06	6.87E-04
Pden_2395		hypothetical protein	2.56	2.39E-05	6.88E-04
Pden_2406	*ddl*	D-alanine--D-alanine ligase	-3.91	1.85E-05	6.87E-04
Pden_2410		conserved hypothetical protein	-1.29	0.001263209	0.004904621
Pden_2412		type I secretion membrane fusion protein, HlyD family	-1.14	0.002378318	0.00757799
Pden_2413		ABC transporter related protein	-1.18	0.003088017	0.009202254
Pden_2414		hypothetical protein	-1.85	0.003422509	0.009890503
Pden_2417	*tnpB*	IS66 Orf2 family protein	2.73	3.70E-04	0.002309828
Pden_2419		phage integrase family protein	-4.07	9.99E-04	0.004214886
Pden_2423		hypothetical protein	2.34	4.65E-05	8.76E-04
Pden_2428		hypothetical protein	2.93	1.75E-04	0.001512937
Pden_2436		hypothetical protein	-2.63	0.002658519	0.008227434
Pden_2437		hypothetical protein	-1.65	9.34E-04	0.004038014
Pden_2443	*tssB*	Uncharacterized conserved protein UCP028301	-3.42	6.51E-05	9.41E-04
Pden_2444	*tssC*	protein of unknown function DUF877	-3.91	5.38E-05	9.01E-04
Pden_2445		protein of unknown function DUF796	-3.23	9.91E-04	0.004196995
Pden_2450	*tssJ*	conserved hypothetical protein	-4.03	4.97E-04	0.002744854
Pden_2451	*tssK*	protein of unknown function DUF876	-3.03	0.002113209	0.007015853
Pden_2457		conserved hypothetical protein	1.21	0.003031062	0.009075263
Pden_2459		OmpA/MotB domain protein	-2.25	0.00141898	0.005313828
Pden_2462		peptidase C14, caspase catalytic subunit p20	1.67	0.001962766	0.006668949
Pden_2465		Hemolysin-type calcium-binding region	1.58	5.95E-04	0.003066191
Pden_2468		cation diffusion facilitator family transporter	2.51	5.70E-04	0.002982562
Pden_2469		carbohydrate ABC transporter ATP-binding protein, CUT1 family	-2.78	1.69E-05	6.87E-04
Pden_2470		carbohydrate ABC transporter membrane protein 2, CUT1 family	-3.69	9.66E-05	0.001123928
Pden_2471		carbohydrate ABC transporter membrane protein 1, CUT1 family	-4.12	1.62E-05	6.87E-04
Pden_2472		carbohydrate ABC transporter substrate-binding protein, CUT1 family	-3.32	1.09E-05	6.87E-04
Pden_2476		glutamine--fructose-6-phosphate transaminase	-1.80	0.003207439	0.00945793
Pden_2479		hypothetical protein	-1.55	0.001878872	0.006466844
Pden_2480		cytochrome c oxidase, subunit III	-1.72	9.13E-04	0.003980302
Pden_2483		nitric oxide reductase, NorB subunit apoprotein	-4.22	7.55E-07	6.87E-04
Pden_2484		nitric oxide reductase, NorC subunit apoprotein	-4.00	5.39E-05	9.01E-04
Pden_2486	*nirI*	FMN-binding domain protein	3.42	5.72E-04	0.002990456
Pden_2497	*katG*	catalase/peroxidase HPI	-2.91	2.10E-04	0.001676926
Pden_2502		protein of unknown function DUF81	2.92	1.27E-04	0.001290694
Pden_2506	*gpmI*	phosphoglycerate mutase	1.31	9.05E-04	0.003955843
Pden_2507		peptidase M23B	1.50	0.002446791	0.007737728
Pden_2508		carboxyl-terminal protease	-1.14	0.001085567	0.004436017

Pden_2510		lytic murein transglycosylase	-2.03	0.001109926	0.004488461
Pden_2512		YbaK/prolyl-tRNA synthetase associated region	-1.71	0.001082248	0.004432197
Pden_2518	*rsfS*	iojap-like protein	-1.67	0.001055549	0.004364031
Pden_2524		conserved hypothetical protein	-2.31	5.75E-05	9.13E-04
Pden_2525		cobalt transporter, subunit CbtA	-1.86	1.78E-04	0.001530905
Pden_2526		conserved hypothetical protein	-1.33	0.002443814	0.007733144
Pden_2528		conserved hypothetical protein	-2.56	2.18E-04	0.001706774
Pden_2529	*cobO*	cob(I)yrinic acid a,c-diamide adenosyltransferase	-1.86	1.22E-04	0.001269567
Pden_2537		precorrin-6A reductase	-2.04	6.48E-04	0.003219899
Pden_2544		cytochrome c, class II	-2.18	2.00E-04	0.001641009
Pden_2547		short-chain dehydrogenase/reductase SDR	2.57	1.31E-04	0.001314735
Pden_2550		electron transfer flavoprotein beta-subunit	-1.43	0.001626141	0.005840986
Pden_2560		Enoyl-CoA hydratase/isomerase	-1.27	9.88E-04	0.004188746
Pden_2565		putative esterase	-1.41	0.001726481	0.006069778
Pden_2566		hypothetical protein	-3.52	7.12E-05	9.80E-04
Pden_2572	*rpe*	ribulose-5-phosphate 3-epimerase	-1.05	0.003047267	0.009099135
Pden_2578		conserved hypothetical protein	-1.50	3.64E-04	0.002303161
Pden_2591	*rimP*	protein of unknown function DUF150	2.34	1.96E-05	6.87E-04
Pden_2594	*infB*	bacterial translation initiation factor 2 (bIF-2)	1.37	7.85E-04	0.003626579
Pden_2596	*mutT*	mutator MutT protein	-1.14	0.001995376	0.006753066
Pden_2598		PpiC-type peptidyl-prolyl cis-trans isomerase	-1.64	1.08E-04	0.001177715
Pden_2602	*ilvE*	branched chain amino acid aminotransferase apoenzyme	-2.04	1.95E-04	0.001628537
Pden_2604	*raiA*	SSU ribosomal protein S30P / sigma 54 modulation protein	-2.88	0.002555576	0.008001781
Pden_2610		TonB-dependent receptor	-4.52	1.54E-04	0.001458681
Pden_2626		type III secretion exporter	1.33	0.0018847	0.006469312
Pden_2627		flagellar biosynthetic protein FliR	1.65	0.002171816	0.007144838
Pden_2629		hypothetical protein	3.59	6.77E-04	0.003306024
Pden_2630		flagellar motor protein	2.76	3.73E-04	0.002309828
Pden_2643		hypothetical protein	-4.09	6.12E-05	9.31E-04
Pden_2644		phage integrase family protein	-4.67	3.20E-04	0.002159975
Pden_2649	*fliF*	flagellar M-ring protein FliF	1.79	5.56E-04	0.002936497
Pden_2659	*mepA*	murein endopeptidase, Metallo peptidase, MEROPS family M74	-1.31	0.001259646	0.004894542
Pden_2663		acetyl-CoA acetyltransferase	-3.67	1.68E-06	6.87E-04
Pden_2664		putative transcriptional regulator, ModE family	-2.35	1.09E-04	0.001182514
Pden_2678		protein of unknown function DUF490	-1.61	0.001562613	0.005704043
Pden_2679		surface antigen (D15)	-1.18	0.001241864	0.004844257
Pden_2680		glucokinase	3.45	1.32E-05	6.87E-04
Pden_2681		hypothetical protein	2.16	4.97E-04	0.002744854
Pden_2684		conserved hypothetical protein	-4.25	8.04E-04	0.003682344
Pden_2688		inositol monophosphatase	1.36	0.001318598	0.005034736
Pden_2699	*flgI*	flagellar P-ring protein	-1.60	0.00260659	0.008101389
Pden_2703		transposase, IS4 family	1.51	7.16E-04	0.003403276
Pden_2704		transposase-like protein	1.45	0.001011269	0.004248664
Pden_2709		saccharopine dehydrogenase (NAD+, L-lysine-forming)	-1.83	8.92E-05	0.001075115
Pden_2711		conserved hypothetical protein	-1.56	4.37E-04	0.0025457
Pden_2718	*purH*	IMP cyclohydrolase	1.28	0.002701697	0.008311152
Pden_2732		protein of unknown function DUF81	-1.80	1.04E-04	0.001161644
Pden_2733		conserved hypothetical protein	-1.66	7.94E-04	0.003647678
Pden_2735		glycerophosphoryl diester phosphodiesterase	-1.67	2.81E-04	0.002001287
Pden_2741		hypothetical protein	2.84	0.001754427	0.006137651
Pden_2743	*efp*	translation elongation factor P (EF-P)	2.35	4.13E-04	0.002453976
Pden_2745		multiple monosaccharide-binding protein	-4.82	0.002557977	0.008004349
Pden_2746		multiple monosaccharide ABC transporter ATP-binding protein	-4.25	0.002448629	0.007738707
Pden_2747		multiple monosaccharide ABC transporter membrane protein	-3.87	0.001614671	0.005818563
Pden_2754		short-chain dehydrogenase/reductase SDR	-1.20	0.002108383	0.007005405
Pden_2755		aldehyde dehydrogenase	-1.90	1.91E-04	0.001609111
Pden_2761		type I secretion target repeat protein	2.51	6.31E-04	0.003164466
Pden_2763		outer membrane porin	-2.16	7.42E-04	0.003490386
Pden_2768		HI0933 family protein	3.35	0.002569863	0.00802847
Pden_2770		major facilitator superfamily MFS_1	-1.52	4.91E-04	0.002726957
Pden_2781		two component transcriptional regulator, Fis family	-1.82	1.01E-04	0.001137092
Pden_2782		histone family protein nucleoid-structuring protein H-NS	-3.77	0.003403129	0.009867016
Pden_2793	*trxA*	thioredoxin	-1.70	1.80E-04	0.001538995
Pden_2800		conserved hypothetical protein	1.27	7.17E-04	0.003406925
Pden_2801	*truB*	tRNA pseudouridine synthase B	1.91	9.67E-04	0.00411907
Pden_2802		conserved hypothetical protein	-2.06	0.001299508	0.004984399
Pden_2803		beta-lactamase domain protein	-3.33	1.79E-04	0.001533089
Pden_2811		UDP-sulfoquinovose synthase	-1.60	9.77E-04	0.004148874
Pden_2819		signal transduction histidine kinase	1.36	9.51E-04	0.00407864
Pden_2828	*fdxH*	formate dehydrogenase (quinone-dependent) iron-sulfur subunit	-1.63	0.003227469	0.009483852
Pden_2829	*fdnG*	formate dehydrogenase (quinone-dependent) catalytic subunit	-2.33	6.19E-04	0.003143078
Pden_2830		formate dehydrogenase (quinone-dependent) catalytic subunit	-3.58	4.07E-05	8.15E-04
Pden_2831		ferredoxin	-1.37	0.002652684	0.008219791
Pden_2832		Rieske (2Fe-2S) domain protein	-1.65	0.002286165	0.007363138
Pden_2835	*recF*	DNA replication and repair protein RecF	1.78	3.66E-04	0.002309564
Pden_2842	*dapE*	succinyldiaminopimelate desuccinylase	1.51	0.001106811	0.004479442
Pden_2843		GCN5-related N-acetyltransferase	1.37	7.68E-04	0.003578439
Pden_2847		transcriptional regulator, TetR family	2.61	1.06E-05	6.87E-04
Pden_2849		PTS system fructose subfamily IIA component	1.74	0.001117876	0.004513404
Pden_2852	*pckA*	Phosphoenolpyruvate carboxykinase (ATP)	-5.26	2.35E-05	6.88E-04
Pden_2856	*fdhF*	formate dehydrogenase alpha subunit	-1.02	0.003412034	0.009872234
Pden_2870		acetyl-CoA acetyltransferase	-2.91	0.002241761	0.007272039
Pden_2871		3-hydroxyacyl-CoA dehydrogenase	-2.55	2.06E-04	0.001670021
Pden_2872		PfkB domain protein	1.91	0.001713644	0.006047193
Pden_2878	*atpE*	ATP synthase F0 subcomplex C subunit	1.06	0.003104384	0.009239266
Pden_2880		conserved hypothetical protein	1.87	0.002342698	0.007497521
Pden_2881		transcriptional regulator, ArsR family	2.23	3.46E-04	0.002238614

Pden_2883		protein of unknown function DUF952		1.86	0.001938934	0.006619569
Pden_2886		conserved hypothetical protein	3	2.85	6.40E-04	0.003193022
Pden_2887		putative phage host specificity protein	3	3.47	0.002264863	0.007322477
Pden_2890		conserved hypothetical protein	3	3.01	8.85E-04	0.003887375
Pden_2896		head-tail adaptor, putative	3	3.21	0.001708381	0.006035961
Pden_2897		conserved hypothetical protein	3	2.67	6.91E-04	0.003324257
Pden_2898		phage major capsid protein, HK97 family	3	1.86	5.98E-04	0.003074387
Pden_2901		phage portal protein, HK97 family	3	5.72	8.87E-06	6.87E-04
Pden_2902		protein of unknown function DUF264		5.34	7.85E-04	0.003626579
Pden_2906		acyl-CoA dehydrogenase domain protein		-1.79	0.001595006	0.005776476
Pden_2908		short-chain dehydrogenase/reductase SDR		-1.79	0.001700567	0.006016749
Pden_2913		Cobyrinic acid a,c-diamide synthase		-1.72	5.27E-04	0.002843404
Pden_2914		conserved hypothetical protein		2.63	2.10E-04	0.001676926
Pden_2915		conserved hypothetical protein		2.04	1.57E-04	0.00147988
Pden_2922		transcriptional regulator, GntR family		2.06	0.001522235	0.00560347
Pden_2924		protein of unknown function DUF162		-1.92	0.001220147	0.00478884
Pden_2925		iron-sulfur cluster binding protein		-1.95	0.002600709	0.008099224
Pden_2926		protein of unknown function DUF224, cysteine-rich region domain protein		-3.81	0.001518839	0.005602074
Pden_2927		L-lactate transport		-3.67	4.86E-04	0.0027113
Pden_2928	*dld*	D-lactate dehydrogenase		1.84	1.49E-04	0.001431346
Pden_2939		hypothetical protein		2.79	1.99E-05	6.87E-04
Pden_2940		transcriptional regulator, TetR family		2.45	2.47E-04	0.001838028
Pden_2941		efflux transporter, RND family, MFP subunit		3.43	1.66E-04	0.001500534
Pden_2942		transporter, hydrophobe/amphiphile efflux-1 (HAE1) family		2.59	1.09E-04	0.00118008
Pden_2943		RND efflux system, outer membrane lipoprotein, NodT family		3.06	9.36E-05	0.001107509
Pden_2944	*thiC*	hydroxymethylpyrimidine synthase		2.80	1.69E-04	0.001507924
Pden_2945		Exopolysaccharide synthesis, ExoD		2.47	6.19E-04	0.003143078
Pden_2946		PfkB domain protein		1.70	6.43E-04	0.003202373
Pden_2947		conserved hypothetical protein		-1.87	8.63E-04	0.003825716
Pden_2948		antifreeze protein, type I		-2.53	1.26E-04	0.001288234
Pden_2954		protein of unknown function UPF0118		1.76	3.77E-04	0.002311838
Pden_2968		hypothetical protein		2.70	9.87E-04	0.004188561
Pden_2970	*rpsI*	SSU ribosomal protein S9P		2.16	8.43E-04	0.003795331
Pden_2972		hypothetical protein		2.98	4.69E-04	0.002650816
Pden_2984		Methyltransferase type 11		1.85	6.74E-04	0.003302397
Pden_2985		transcriptional regulator, XRE family		3.37	1.63E-04	0.001496408
Pden_2986		PEP phosphonomutase and related enzymes-like protein		5.07	2.25E-05	6.88E-04
Pden_2988		hypothetical protein		-2.13	0.001470022	0.005462966
Pden_2990		two component transcriptional regulator, LuxR family		-2.71	1.67E-04	0.001503857
Pden_2998		conserved hypothetical protein		3.48	2.32E-05	6.88E-04
Pden_2999		MxaA protein, putative		4.13	7.94E-05	0.001019694
Pden_3000		von Willebrand factor, type A		4.12	1.44E-04	0.001397768
Pden_3001		conserved hypothetical protein		3.91	3.75E-04	0.002309828
Pden_3002		von Willebrand factor, type A		3.84	3.48E-05	7.44E-04
Pden_3009		chorismate mutase related enzyme		2.04	1.79E-04	0.001533089
Pden_3030		Methyltransferase type 12		3.15	3.99E-05	8.15E-04
Pden_3031		transport system permease protein		3.74	6.12E-05	9.31E-04
Pden_3032		ABC transporter related protein		3.60	2.94E-06	6.87E-04
Pden_3036		sulphate transporter		3.47	9.27E-06	6.87E-04
Pden_3037		Cl- channel, voltage-gated family protein		-1.16	0.00166131	0.005923996
Pden_3038		choline/carnitine/betaine transporter		-2.11	7.08E-04	0.003381724
Pden_3039		hypothetical protein		-2.12	3.48E-04	0.002243683
Pden_3046		Alcohol dehydrogenase, zinc-binding domain protein		1.36	5.84E-04	0.003033472
Pden_3049		Transketolase, central region		-1.48	3.77E-04	0.002311838
Pden_3050		regulatory protein, IclR		-2.52	0.003309041	0.009695414
Pden_3051		Endoribonuclease L-PSP		-2.31	0.001684712	0.005980919
Pden_3052		aminotransferase		-1.62	1.81E-04	0.001543104
Pden_3053		Amidase		-1.48	4.06E-04	0.002419718
Pden_3057		amino acid/amide ABC transporter membrane protein 1, HAAT family		-1.84	0.002404502	0.007632598
Pden_3058		amino acid/amide ABC transporter membrane protein 2, HAAT family		-2.48	6.53E-04	0.003231196
Pden_3059	*iaaH*	Amidase		-2.60	0.001391354	0.0052336
Pden_3061		outer membrane autotransporter barrel domain		1.98	2.91E-04	0.00202724
Pden_3067		conserved hypothetical protein		2.77	5.84E-05	9.13E-04
Pden_3069		transcriptional regulator, LysR family		2.25	4.73E-04	0.002669604
Pden_3071		aconitase		-2.85	2.09E-05	6.87E-04
Pden_3073		protein of unknown function DUF112, transmembrane		-4.23	1.38E-05	6.87E-04
Pden_3074		putative tricarboxylic transport membrane protein		-5.23	0.001139941	0.004578915
Pden_3075		Uncharacterized protein UPF0065		-4.22	1.12E-04	0.001190496
Pden_3077		gene transfer agent (GTA) like protein		4.33	3.87E-05	7.97E-04
Pden_3078		conserved hypothetical protein		2.30	0.00172509	0.006069582
Pden_3080		conserved hypothetical protein		-2.00	8.41E-04	0.003795331
Pden_3081		hypothetical protein		-4.63	0.003389514	0.009845731
Pden_3082		outer membrane autotransporter barrel domain		-4.03	2.48E-05	6.90E-04
Pden_3083		putative outer membrane protein		-2.68	8.68E-04	0.003833364
Pden_3086		hypothetical protein		-3.26	0.002027235	0.006816224
Pden_3092		conserved hypothetical protein		1.27	0.003324695	0.009722909
Pden_3099		HupE/UreJ protein		-1.94	2.01E-04	0.001641009
Pden_3108	*hypB*	hydrogenase accessory protein HypB		-1.22	0.001727543	0.006069778
Pden_3109		two component, sigma54 specific, transcriptional regulator, Fis family		-1.08	0.003217907	0.009468264
Pden_3110	*hypC*	hydrogenase assembly chaperone hypC/hupF		-1.77	0.002412303	0.007652563
Pden_3111	*hypD*	hydrogenase expression/formation protein HypD		-2.09	6.99E-05	9.69E-04
Pden_3113		hypothetical protein		2.80	1.46E-05	6.87E-04
Pden_3122		phage integrase family protein		-3.82	8.30E-04	0.003764313
Pden_3125		conserved hypothetical protein		1.26	5.33E-04	0.002866564
Pden_3131	*chrA*	chromate transporter, chromate ion transporter (CHR) family		2.96	2.31E-04	0.001743752
Pden_3132		Rhodanese domain protein		3.09	4.24E-05	8.32E-04
Pden_3133	*arsH*	NADPH-dependent FMN reductase		1.41	4.69E-04	0.002650816

Pden_3134	*arsB*	arsenical-resistance protein	2.05	2.31E-04	0.001746437
Pden_3135	*arsC*	arsenate reductase	2.70	6.27E-05	9.34E-04
Pden_3136		transcriptional regulator, ArsR family	2.27	5.40E-04	0.002881826
Pden_3148		protease Do	-1.60	1.77E-04	0.001524591
Pden_3149		Phytanoyl-CoA dioxygenase	3.53	3.74E-04	0.002309828
Pden_3153		Cupin 2, conserved barrel domain protein	1.56	0.001142772	0.004581039
Pden_3155		Methyltransferase type 12	2.74	0.001264372	0.004905378
Pden_3160		transposase IS116/IS110/IS902 family protein	-2.43	9.47E-04	0.004070411
Pden_3163		conserved hypothetical protein	2.91	8.45E-04	0.003801247
Pden_3164		DNA methylase N-4/N-6 domain protein	4.90	1.10E-04	0.001182514
Pden_3165		hypothetical protein	3.11	8.59E-05	0.001060694
Pden_3166		protein of unknown function DUF81	2.08	2.14E-04	0.001694513
Pden_3167		single-strand binding protein	4.21	5.83E-05	9.13E-04
Pden_3169		hypothetical protein	3.17	6.62E-06	6.87E-04
Pden_3170		hypothetical protein	3.73	1.59E-05	6.87E-04
Pden_3171		transcriptional regulator, XRE family	1.82	0.002793347	0.00852433
Pden_3172		hypothetical protein	1.53	2.19E-04	0.001708297
Pden_3173		hypothetical protein	3.64	6.23E-04	0.003147257
Pden_3174		conserved hypothetical protein	4.09	1.46E-05	6.87E-04
Pden_3176		conserved hypothetical protein	1.92	0.002359484	0.00753695
Pden_3178		putative transcriptional regulator	3.91	1.93E-04	0.00161701
Pden_3179		replication protein A	2.96	0.001369625	0.005178798
Pden_3181		conserved hypothetical protein	3.50	1.34E-05	6.87E-04
Pden_3182		C-5 cytosine-specific DNA methylase	4.42	4.19E-06	6.87E-04
Pden_3183		conjugation peptidase TraF, Serine peptidase, MEROPS family S26C	3.79	9.25E-05	0.001100653
Pden_3187		conserved hypothetical protein	2.51	1.51E-04	0.001444647
Pden_3188		TRAG family protein	1.53	0.001306954	0.005005376
Pden_3190		conserved hypothetical protein	2.60	3.49E-04	0.002248218
Pden_3191		hypothetical protein	1.97	6.24E-04	0.003147257
Pden_3192	*vapC*	PilT protein domain protein	2.44	0.00165802	0.005923996
Pden_3193		putative plasmid stabilization protein	2.29	0.002291506	0.007366284
Pden_3194	*trbB*	type II secretion system protein E	2.26	3.85E-04	0.00233751
Pden_3202	*trbG*	Conjugal transfer protein TrbG/VirB9/CagX	1.60	3.46E-04	0.002238614
Pden_3203		conjugation TrbI family protein	1.45	4.79E-04	0.002686576
Pden_3219	*dctP*	TRAP dicarboxylate transporter- DctP subunit	-1.63	0.002018207	0.006807584
Pden_3221		Uncharacterized protein UPF0065	-2.03	0.001612147	0.005813604
Pden_3230		AMP-dependent synthetase and ligase	1.15	0.001090488	0.004446969
Pden_3233		transposase IS116/IS110/IS902 family protein	-1.33	0.001340281	0.005102136
Pden_3235		conserved hypothetical protein	-2.97	8.35E-04	0.003775583
Pden_3245		conserved hypothetical protein	-1.68	0.003430816	0.009903205
Pden_3247		transposase	2.07	7.07E-04	0.003380658
Pden_3260		aldehyde dehydrogenase	-1.20	0.001169112	0.004646598
Pden_3266		Uncharacterized protein UPF0065	-2.49	2.24E-04	0.00171547
Pden_3267		amidohydrolase 2	-2.47	2.74E-05	7.03E-04
Pden_3268		protein of unknown function DUF112, transmembrane	-1.74	8.62E-04	0.003825716
Pden_3280		Integrase, catalytic region	2.84	0.002536431	0.007976368
Pden_3284		two component transcriptional regulator, winged helix family	1.38	9.13E-04	0.003980302
Pden_3294		Heavy metal transport/detoxification protein	-1.71	1.69E-04	0.001507924
Pden_3295		heavy metal translocating P-type ATPase	-2.40	3.80E-05	7.91E-04
Pden_3297	*cadA*	heavy metal translocating P-type ATPase	1.94	6.74E-04	0.003302397
Pden_3298		conserved hypothetical membrane protein	1.54	9.31E-04	0.004030546
Pden_3299		ABC transporter related protein	2.48	3.99E-04	0.002394246
Pden_3300		protein of unknown function DUF214	2.35	1.24E-04	0.001274131
Pden_3301		efflux transporter, RND family, MFP subunit	2.73	1.79E-04	0.001533089
Pden_3302		transcriptional regulator, TetR family	2.99	7.13E-04	0.00340186
Pden_3306		conserved hypothetical protein	3.56	8.60E-05	0.001060694
Pden_3308	*tnpB*	IS66 Orf2 family protein	3.22	6.54E-04	0.003232982
Pden_3309		transposase IS3/IS911 family protein	3.80	2.23E-04	0.00171547
Pden_3310		hypothetical protein	4.80	0.002554418	0.008001781
Pden_3311		response regulator receiver protein	1.49	0.001674186	0.005952232
Pden_3312		integral membrane sensor signal transduction histidine kinase	1.73	2.97E-04	0.002053033
Pden_3327		hypothetical protein	5.05	2.25E-05	6.88E-04
Pden_3334		hypothetical protein	3.98	0.002321676	0.007449077
Pden_3337	*chrA*	chromate transporter, chromate ion transporter (CHR) family	2.96	2.30E-04	0.001743752
Pden_3338		Rhodanese domain protein	3.09	4.24E-05	8.32E-04
Pden_3339	*arsH*	NADPH-dependent FMN reductase	1.49	2.89E-04	0.002019349
Pden_3340	*arsB*	arsenical-resistance protein	1.66	1.04E-04	0.001161644
Pden_3341		arsenate reductase	3.01	2.83E-05	7.03E-04
Pden_3342		transcriptional regulator, ArsR family	2.77	1.46E-05	6.87E-04
Pden_3344		sulphate transporter	4.74	6.43E-07	6.87E-04
Pden_3345		protein of unknown function DUF1127	2.69	5.02E-04	0.002762958
Pden_3346		hypothetical protein	2.60	4.16E-04	0.002464843
Pden_3347		Glyoxalase/bleomycin resistance protein/dioxygenase	2.30	4.81E-05	8.86E-04
Pden_3348		Methyltransferase type 12	2.63	4.33E-04	0.002538599
Pden_3351		Methyltransferase type 11	2.08	7.70E-05	0.001002704
Pden_3352		protein of unknown function DUF156	2.41	7.32E-05	9.81E-04
Pden_3353	*dmeF*	cation diffusion facilitator family transporter	2.15	5.48E-05	9.01E-04
Pden_3354		hypothetical protein	1.51	0.00151569	0.005597329
Pden_3357		DNA methylase N-4/N-6 domain protein	5.01	1.57E-06	6.87E-04
Pden_3358		hypothetical protein	4.60	5.57E-05	9.01E-04
Pden_3359		GCN5-related N-acetyltransferase	1.68	5.50E-04	0.002920173
Pden_3360		hypothetical protein	3.57	0.002186885	0.007171114
Pden_3361		single-strand binding protein	3.71	5.66E-04	0.00297487
Pden_3364		hypothetical protein	2.23	2.90E-05	7.04E-04
Pden_3365		conserved hypothetical protein	3.99	5.12E-04	0.002788261
Pden_3366		hypothetical protein	2.16	1.00E-04	0.001137092
Pden_3367		conserved hypothetical protein	1.62	0.002550477	0.007995707

Pden_3368		transcriptional regulator, XRE family	1.65	0.001351901	0.005130941
Pden_3369		hypothetical protein	5.03	7.26E-06	6.87E-04
Pden_3370		putative replication protein A	3.62	2.63E-05	7.00E-04
Pden_3371		conserved hypothetical protein	2.95	1.96E-05	6.87E-04
Pden_3372		C-5 cytosine-specific DNA methylase	2.70	6.45E-05	9.41E-04
Pden_3373		conjugation peptidase TraF, Serine peptidase, MEROPS family S26C	2.69	2.64E-04	0.00192913
Pden_3374		protein of unknown function DUF736	2.16	1.17E-04	0.00122534
Pden_3375		Lytic transglycosylase, catalytic	2.17	4.70E-05	8.81E-04
Pden_3376		hypothetical protein	2.05	1.10E-04	0.001182514
Pden_3377		conserved hypothetical protein	3.12	4.39E-05	8.44E-04
Pden_3378		prevent-host-death family protein	2.07	9.96E-04	0.004207138
Pden_3379	*vapC*	PilT protein domain protein	2.27	1.41E-04	0.001376063
Pden_3385		Tripartite ATP-independent periplasmic transporter, DctQ component	-1.36	0.003341303	0.009739216
Pden_3386		cyclase family protein	-2.12	2.21E-04	0.001708297
Pden_3387		TRAP dicarboxylate transporter, DctP subunit	-2.56	8.83E-04	0.003885314
Pden_3399		Ferric reductase domain protein transmembrane component, N-terminal domain	2.58	0.001833622	0.00634995
Pden_3400		molybdenum cofactor synthesis domain	4.13	3.15E-06	6.87E-04
Pden_3401		transposase, IS4 family	2.39	0.002178103	0.007156221
Pden_3402		integral membrane sensor signal transduction histidine kinase	1.79	0.002342293	0.007497521
Pden_3410		hypothetical protein	3.26	9.49E-05	0.001117559
Pden_3411		hypothetical protein	4.15	3.31E-06	6.87E-04
Pden_3412		hypothetical protein	1.76	2.10E-04	0.001676926
Pden_3415		transcriptional regulator, LysR family	4.30	2.47E-04	0.001836606
Pden_3423		transcriptional regulator, LysR family	3.03	7.35E-04	0.003469659
Pden_3424		dihydrodipicolinate synthetase	-2.27	2.67E-05	7.01E-04
Pden_3425		branched chain amino acid: 2-keto-4-methylthiobutyrate aminotransferase	-2.84	1.69E-05	6.87E-04
Pden_3426	*pxpA*	LamB/YcsF family protein	-2.40	6.36E-04	0.003177863
Pden_3427		TRAP dicarboxylate transporter- DctP subunit	-3.40	0.001863084	0.00642561
Pden_3428		Tripartite ATP-independent periplasmic transporter, DctQ component	-2.56	2.13E-04	0.001694513
Pden_3429		TRAP dicarboxylate transporter, DctM subunit	-1.95	1.62E-04	0.001494676
Pden_3447		efflux transporter, RND family, MFP subunit	-1.29	8.81E-04	0.003878754
Pden_3448		transporter, hydrophobe/amphiphile efflux-1 (HAE1) family	-1.73	2.07E-04	0.00167015
Pden_3450		beta-lactamase	-2.97	4.80E-05	8.86E-04
Pden_3452		hypothetical protein	1.18	0.003160719	0.009336478
Pden_3453		hypothetical protein	2.25	4.88E-05	8.89E-04
Pden_3457		FAD dependent oxidoreductase	1.55	0.001528994	0.005613704
Pden_3458	*argE*	acetylornithine deacetylase ArgE, Metallo peptidase, MEROPS family M20A	2.46	4.86E-05	8.89E-04
Pden_3461		binding-protein-dependent transport systems inner membrane component	-2.55	2.15E-04	0.001694513
Pden_3462		binding-protein-dependent transport systems inner membrane component	-2.56	2.89E-04	0.002020686
Pden_3463		FAD-dependent pyridine nucleotide-disulfide oxidoreductase	1.65	1.93E-04	0.001613699
Pden_3465		Rieske (2Fe-2S) domain protein	-2.30	6.31E-04	0.003164466
Pden_3467		proline racemase	2.35	2.13E-04	0.001694513
Pden_3469		hypothetical protein	3.10	9.89E-06	6.87E-04
Pden_3473		adenylyl cyclase class-3/4/guanylyl cyclase	2.28	1.24E-04	0.001274131
Pden_3474		protein of unknown function DUF839	2.03	5.05E-05	9.00E-04
Pden_3476		NADH:flavin oxidoreductase/NADH oxidase	1.16	0.002980214	0.008949481
Pden_3477		putative transcriptional regulator, MerR family	1.32	0.00271666	0.008330981
Pden_3481		Tripartite ATP-independent periplasmic transporter, DctQ component	-1.26	0.001526695	0.005613405
Pden_3489	*pcaQ*	transcriptional regulator, LysR family	2.89	0.001160055	0.004631986
Pden_3494		protocatechuate 3,4-dioxygenase, alpha subunit	-1.33	0.002096468	0.006983169
Pden_3497		TRAP dicarboxylate transporter- DctP subunit	-2.26	9.26E-05	0.001100653
Pden_3501		Glyoxalase/bleomycin resistance protein/dioxygenase	2.22	9.44E-04	0.004070411
Pden_3507		Amidohydrolase 3	1.78	3.21E-04	0.002168465
Pden_3508		protein of unknown function DUF894, DitE	2.33	9.14E-04	0.003980302
Pden_3512		transcriptional regulator, MarR family	1.48	0.001083495	0.004432197
Pden_3513		transcriptional regulator, LysR family	1.66	3.38E-04	0.002227917
Pden_3514		Allantoinase	3.34	8.41E-06	6.87E-04
Pden_3515		amidase, hydantoinase/carbamoylase family	3.37	1.66E-04	0.001500534
Pden_3516		protein of unknown function DUF917	1.48	0.001528157	0.005613704
Pden_3517	*hutU*	urocanate hydratase	1.99	5.60E-05	9.01E-04
Pden_3525		ABC transporter related protein	1.66	0.002639881	0.008199827
Pden_3526		conserved hypothetical protein	2.29	3.19E-05	7.09E-04
Pden_3528		transcriptional regulator, IclR family	1.21	0.003363561	0.009787189
Pden_3529		ABC transporter related protein	3.21	0.002288358	0.007365516
Pden_3533		transport system permease protein	2.26	1.10E-04	0.001182514
Pden_3534		transport system permease protein	3.96	6.35E-04	0.0031761
Pden_3535		Glutathione S-transferase, N-terminal domain	-2.43	9.26E-04	0.004022714
Pden_3536		transcriptional regulator, GntR family	-1.70	0.002468688	0.007792374
Pden_3538		TRAP-T family transporter, DctQ (4 TMs) subunit	-2.39	0.002512912	0.007922088
Pden_3540		TRAP dicarboxylate transporter- DctP subunit	-4.97	2.19E-04	0.001708297
Pden_3541		short-chain dehydrogenase/reductase SDR	-3.98	1.29E-04	0.001304482
Pden_3542		short-chain dehydrogenase/reductase SDR	-3.22	4.95E-04	0.002741428
Pden_3543		fumarate reductase/succinate dehydrogenase flavoprotein domain protein	-3.04	7.01E-04	0.003359157
Pden_3544		hypothetical protein	-2.16	0.00224208	0.007272039
Pden_3550	*pseF*	acylneuraminate cytidylyltransferase	1.64	4.42E-04	0.00255247
Pden_3551		flagellin modification protein FlmD	1.76	1.89E-04	0.00159937
Pden_3553		NAD-dependent epimerase/dehydratase	1.09	0.001348411	0.005121532
Pden_3557	*pseC*	DegT/DnrJ/EryC1/StrS aminotransferase	1.16	0.002886005	0.008739141
Pden_3561		transposase IS116/IS110/IS902 family protein	-2.43	9.58E-04	0.004097585
Pden_3566		two component transcriptional regulator, winged helix family	2.16	2.57E-05	7.00E-04
Pden_3574		amino acid/amide ABC transporter substrate-binding protein, HAAT family	-2.58	0.002228419	0.007255299
Pden_3578		protein of unknown function DUF849	-2.66	0.001912924	0.006548435
Pden_3579	*hisD*	histidinol dehydrogenase	-1.97	0.002163163	0.007125631
Pden_3583		short-chain dehydrogenase/reductase SDR	-2.19	3.39E-04	0.002230025
Pden_3584		FAD-linked oxidoreductase	-3.06	2.35E-05	6.88E-04
Pden_3585	*eutH*	Ethanolamine utilization protein, EutH	-1.81	0.002369436	0.007559204
Pden_3586		transglutaminase domain protein	-2.70	1.60E-05	6.87E-04

Pden_3587		conserved hypothetical protein		-1.91	0.001278709	0.004938294
Pden_3595	*tnpB*	IS66 Orf2 family protein		3.59	3.45E-04	0.002238614
Pden_3596		phage tape measure protein		4.36	6.17E-05	9.33E-04
Pden_3597		hypothetical protein		4.65	6.89E-05	9.64E-04
Pden_3598		hypothetical protein		4.05	2.19E-05	6.88E-04
Pden_3600		hypothetical protein		4.40	5.00E-04	0.002752653
Pden_3601		Fibronectin, type III domain protein		3.30	1.42E-05	6.87E-04
Pden_3604		hypothetical protein		2.77	2.35E-05	6.88E-04
Pden_3608		hypothetical protein		4.86	4.73E-06	6.87E-04
Pden_3611		conserved hypothetical protein		1.69	0.001768755	0.006171016
Pden_3612	*ileS*	Isoleucyl-tRNA synthetase		1.67	2.01E-04	0.001641009
Pden_3614		conserved hypothetical protein		2.04	5.51E-04	0.002920173
Pden_3615		Glyoxalase/bleomycin resistance protein/dioxygenase		2.32	0.001283383	0.004945029
Pden_3617	*rnhA*	ribonuclease H		2.02	0.002108678	0.007005405
Pden_3618		Glutathione S-transferase, C-terminal domain		2.39	0.001732105	0.006081587
Pden_3619	*ispH*	4-hydroxy-3-methylbut-2-enyl diphosphate reductase		1.57	0.001123258	0.004527912
Pden_3622		GCN5-related N-acetyltransferase		2.64	0.001549955	0.005670103
Pden_3624		conserved hypothetical protein		1.59	5.81E-04	0.003020797
Pden_3625		AAA ATPase		1.52	7.88E-04	0.003635439
Pden_3627		Lysine exporter protein (LYSE/YGGA)		3.33	6.76E-05	9.57E-04
Pden_3631	*cobD*	adenosylcobinamide-phosphate synthase		2.71	0.002793401	0.00852433
Pden_3637		Propionyl-CoA carboxylase		-1.46	0.00225985	0.00731094
Pden_3643		major facilitator superfamily MFS_1		4.77	7.20E-04	0.003412359
Pden_3644		conserved hypothetical protein		-1.44	0.001094758	0.00445637
Pden_3651	*aqpZ*	MIP family channel protein		-1.78	4.11E-04	0.002443868
Pden_3652		ABC transporter related protein		2.43	1.00E-04	0.001137092
Pden_3662		FMN adenylyltransferase / riboflavin kinase		2.37	9.60E-05	0.001121864
Pden_3667	*ispDF*	2-C-methyl-D-erythritol 4-phosphate cytidylyltransferase / 2-C-methyl-D-erythritol 2,4-cyclodiphosphate synthase		1.68	0.001724282	0.006069582
Pden_3674		NUDIX hydrolase		3.06	9.02E-06	6.87E-04
Pden_3675		conserved hypothetical protein		1.51	0.001961772	0.006668949
Pden_3678		homoserine dehydrogenase		1.68	2.92E-04	0.002033768
Pden_3679		hypothetical protein		-1.50	7.18E-04	0.003408842
Pden_3684		biotin carboxyl carrier protein / biotin carboxylase		-1.27	0.001936225	0.006614782
Pden_3688		propionyl-CoA carboxylase carboxyltransferase subunit		-2.06	0.002383033	0.007583468
Pden_3690		protein of unknown function DUF140		2.67	0.001689597	0.005990496
Pden_3691		ABC transporter related protein		3.27	0.001000095	0.004216054
Pden_3695		GCN5-related N-acetyltransferase		-1.28	6.75E-04	0.003302397
Pden_3698	*trmJ*	RNA methyltransferase, TrmH family, group 1		2.99	6.86E-05	9.62E-04
Pden_3701	*miaB*	tRNA-i(6)A37 thiotransferase enzyme MiaB		2.83	5.40E-05	9.01E-04
Pden_3702		glutamate--putrescine ligase		-1.94	2.21E-04	0.001708297
Pden_3704		amino acid ABC transporter membrane protein 1, PAAT family		-2.27	1.85E-05	6.87E-04
Pden_3705		amino acid ABC transporter substrate-binding protein, PAAT family		-3.04	3.67E-04	0.002309564
Pden_3710		hypothetical protein		1.58	4.30E-04	0.002528783
Pden_3711	*pyrG*	CTP synthase		1.64	0.002087504	0.006962472
Pden_3712	*secG*	protein translocase subunit secG		2.47	6.60E-05	9.47E-04
Pden_3713	*cobQ*	adenosylcobyric acid synthase (glutamine-hydrolysing)		1.82	0.00266188	0.008227777
Pden_3714		short-chain dehydrogenase/reductase SDR		1.56	0.002210708	0.007225642
Pden_3717	*gltX2*	glutamyl-tRNA synthetase		1.18	0.002930434	0.00883647
Pden_3719		Heme NO binding domain protein		1.99	1.10E-04	0.001182514
Pden_3720		diguanylate cyclase		1.57	4.85E-04	0.002709733
Pden_3721		conserved hypothetical protein		1.89	7.18E-05	9.81E-04
Pden_3722		hypothetical protein		2.99	7.00E-05	9.69E-04
Pden_3727		hypothetical protein		1.69	8.65E-04	0.003825889
Pden_3739		putative phage-related protein	4	1.41	0.00291323	0.008790037
Pden_3742		hypothetical protein	4	2.62	6.60E-04	0.003246886
Pden_3748		hypothetical protein	4	1.91	5.55E-04	0.002935927
Pden_3749		conserved hypothetical protein	4	2.59	0.001132505	0.004557929
Pden_3750		hypothetical protein	4	2.69	6.34E-05	9.35E-04
Pden_3751		conserved hypothetical protein	4	2.82	2.87E-04	0.002014555
Pden_3756		phage terminase, large subunit, PBSX family	4	1.15	0.002946438	0.008870761
Pden_3758		hypothetical protein	4	2.83	2.42E-05	6.88E-04
Pden_3759		putative head-tail connector protein	4	4.30	1.83E-05	6.87E-04
Pden_3760		hypothetical protein	4	4.00	2.87E-04	0.002014555
Pden_3767		conserved hypothetical protein	4	2.48	1.26E-05	6.87E-04
Pden_3768		conserved hypothetical protein	4	3.10	0.001672898	0.005952129
Pden_3769		conserved hypothetical protein		2.08	0.001174601	0.004659024
Pden_3775		hypothetical protein		2.66	7.42E-04	0.003490386
Pden_3776		hypothetical protein		2.87	0.001536336	0.005628414
Pden_3777		hypothetical protein		2.89	0.001487127	0.005520031
Pden_3778		metallophosphoesterase		3.75	1.84E-05	6.87E-04
Pden_3780		Peptidoglycan-binding domain 1 protein		3.49	1.57E-04	0.00147988
Pden_3781		hypothetical protein		3.65	0.002357106	0.007534109
Pden_3784	*cmk*	cytidylate kinase		2.41	0.001012026	0.004248664
Pden_3785		2-nitropropane dioxygenase, NPD		2.27	7.04E-04	0.003368992
Pden_3793	*alkB*	DNA-N1-methyladenine dioxygenase		1.37	0.002143573	0.007074909
Pden_3798		aminotransferase		1.19	0.003118405	0.009239266
Pden_3799		MlrC domain protein		1.37	6.87E-04	0.00331005
Pden_3800		extracellular solute-binding protein, family 5		-1.49	6.02E-04	0.003082284
Pden_3811	*mfd*	transcription-repair coupling factor		2.00	8.69E-05	0.001065159
Pden_3812	*clpA*	ATP-dependent Clp protease, ATP-binding subunit clpA		-2.03	2.58E-04	0.001899163
Pden_3820		conserved hypothetical protein		2.76	0.002651512	0.008219791
Pden_3821		alpha/beta hydrolase fold protein		-1.00	0.002517387	0.007926729
Pden_3828		hypothetical protein		5.37	1.58E-04	0.00147988
Pden_3829		hypothetical protein		5.06	6.53E-07	6.87E-04
Pden_3832		hypothetical protein		2.31	3.45E-04	0.002238614
Pden_3833		conserved hypothetical protein		1.16	0.001567175	0.005716576

Pden_3834		hypothetical protein	4.77	3.50E-05	7.45E-04
Pden_3844		transposase IS116/IS110/IS902 family protein	-2.42	9.71E-04	0.00413176
Pden_3849		phage integrase family protein	-3.79	5.08E-04	0.002784714
Pden_3851		hypothetical protein	4.47	2.80E-05	7.03E-04
Pden_3852		hypothetical protein	5.00	3.45E-06	6.87E-04
Pden_3853		hypothetical protein	3.43	1.77E-05	6.87E-04
Pden_3854		hypothetical protein	3.82	4.31E-04	0.002528783
Pden_3855		hypothetical protein	5.11	4.64E-04	0.002633628
Pden_3856		Substrate-binding region of ABC-type glycine betaine transport system	1.77	1.65E-04	0.001498242
Pden_3857		binding-protein-dependent transport systems inner membrane component	2.52	1.98E-04	0.001638254
Pden_3859		hypothetical protein	1.83	0.001800865	0.006257913
Pden_3860		NUDIX hydrolase	2.17	0.00119233	0.004705196
Pden_3865	*gyrA*	DNA gyrase, A subunit	1.17	0.002709546	0.008319243
Pden_3867		DedA family protein	2.22	0.00215374	0.007101757
Pden_3870		cyclase/dehydrase	1.07	0.001416484	0.005308407
Pden_3871		conserved hypothetical protein	2.09	0.001083049	0.004432197
Pden_3874		conserved hypothetical protein	2.66	3.22E-05	7.09E-04
Pden_3876		protein of unknown function DUF6, transmembrane	-1.40	8.59E-04	0.003825716
Pden_3878	*ffh*	signal recognition particle subunit FFH/SRP54 (srp54)	2.93	7.61E-04	0.003556641
Pden_3879		GCN5-related N-acetyltransferase	2.64	0.002588372	0.008069538
Pden_3887		chromosome partitioning protein	2.19	7.24E-05	9.81E-04
Pden_3890		pyruvate dehydrogenase complex dihydrolipoamide acetyltransferase	-1.58	0.001455555	0.005414752
Pden_3891		Transketolase, central region	-1.80	0.001747949	0.006124474
Pden_3895		conserved hypothetical protein	-1.78	5.36E-04	0.002869505
Pden_3896		protein of unknown function DUF1523	-2.00	0.001532365	0.005621808
Pden_3897	*deoC*	deoxyribose-phosphate aldolase	-1.11	0.003226421	0.009483852
Pden_3911		protein of unknown function DUF81	2.21	7.71E-04	0.003589064
Pden_3912		hypothetical protein	1.52	0.002666452	0.008236879
Pden_3914		protein of unknown function DUF989	-3.14	7.24E-04	0.003427327
Pden_3915	*uraH*	Transthyretin	-2.33	0.001593359	0.005776476
Pden_3916		amidohydrolase	-2.01	3.71E-04	0.002309828
Pden_3917		uracil-xanthine permease	-2.45	0.001083757	0.004432197
Pden_3923		hypothetical protein	2.36	2.41E-04	0.001801579
Pden_3925		transcriptional regulator, SARP family	2.06	0.002653397	0.008219791
Pden_3926	*purM*	phosphoribosylformylglycinamidine cyclo-ligase	1.67	0.001183148	0.004676252
Pden_3927	*purN*	formyltetrahydrofolate-dependent phosphoribosylglycinamide formyltransferase	1.39	3.47E-04	0.002241764
Pden_3928		amino acid/amide ABC transporter membrane protein 2, HAAT family	-2.15	6.84E-04	0.00331005
Pden_3929		amino acid/amide ABC transporter membrane protein 1, HAAT family	-2.64	2.23E-04	0.00171547
Pden_3930		amino acid/amide ABC transporter ATP-binding protein 2, HAAT family	-2.74	6.31E-04	0.003164466
Pden_3931		amino acid/amide ABC transporter ATP-binding protein 1, HAAT family	-3.23	0.001895938	0.006500951
Pden_3932		amino acid/amide ABC transporter substrate-binding protein, HAAT family	-3.79	1.63E-05	6.87E-04
Pden_3936	*gmk*	guanylate kinase	1.60	5.75E-04	0.003000752
Pden_3941	*radA*	DNA replication and repair protein RadA	1.38	3.89E-04	0.002358731
Pden_3946		phospholipase/Carboxylesterase	1.58	6.75E-04	0.003302397
Pden_3948		small multidrug resistance protein	3.45	1.40E-04	0.001373789
Pden_3949		HNH endonuclease	1.96	0.001862602	0.00642561
Pden_3951		Radical SAM domain protein	4.08	2.70E-05	7.01E-04
Pden_3957		amino acid/amide ABC transporter substrate-binding protein, HAAT family	-1.39	7.27E-04	0.003436171
Pden_3961		Mg chelatase, subunit ChII	2.67	3.11E-05	7.09E-04
Pden_3962	*coaBC*	Phosphopantothenoylcysteine decarboxylase / Phosphopantothenate-cysteine ligase	2.08	0.001491023	0.005527823
Pden_3971	*purL*	phosphoribosylformylglycinamidine synthase subunit II	1.35	8.58E-04	0.003825716
Pden_3976		conserved hypothetical protein	4.01	9.52E-06	6.87E-04
Pden_3982		response regulator receiver modulated diguanylate cyclase	2.73	2.06E-04	0.001670021
Pden_3984		aminotransferase	-2.50	2.41E-04	0.001800109
Pden_3985		cytochrome P450	2.09	8.27E-04	0.003758078
Pden_3986		conserved hypothetical protein	2.98	3.98E-04	0.002393936
Pden_3987		peptidase M48, Ste24p	1.88	0.00276867	0.008480205
Pden_3988		VWA containing CoxE family protein	2.43	1.67E-05	6.87E-04
Pden_3989		Ppx/GppA phosphatase	3.24	1.08E-04	0.001177715
Pden_3990	*rlmE (ftsJ rrmJ)*	23S rRNA Um-2552 2'-O-methyltransferase	2.81	3.61E-04	0.002294387
Pden_3997	*dxr*	1-deoxy-D-xylulose 5-phosphate reductoisomerase	2.11	1.73E-04	0.001509649
Pden_3998	*rseP*	site-2 protease, Metallo peptidase, MEROPS family M50B	1.84	0.002811845	0.008560656
Pden_4002		Penicillin amidase	2.43	7.29E-05	9.81E-04
Pden_4006		major facilitator superfamily MFS_1	1.37	8.42E-04	0.003795331
Pden_4015		Linocin_M18 bacteriocin protein	-1.70	4.84E-04	0.002708578
Pden_4016		protein of unknown function UPF0187	-1.74	3.04E-04	0.002090985
Pden_4018	*urtB*	amino acid/amide ABC transporter membrane protein 1, HAAT family	1.86	0.001650375	0.005905195
Pden_4019	*urtC*	amino acid/amide ABC transporter membrane protein 2, HAAT family	2.87	2.84E-04	0.00201284
Pden_4020	*urtD*	ABC transporter related protein	2.32	2.89E-04	0.002019349
Pden_4023	*gph*	phosphoglycolate phosphatase	-1.67	0.00166265	0.005923996
Pden_4025	*typA*	GTP-binding protein TypA	2.77	1.39E-05	6.87E-04
Pden_4027		ferredoxin	1.44	0.002282799	0.007361665
Pden_4029	*purD*	phosphoribosylamine--glycine ligase	2.30	3.78E-04	0.002311838
Pden_4031		conserved hypothetical protein	1.55	9.15E-04	0.003981577
Pden_4032	*mreB*	rod shape-determining protein MreB	2.00	1.14E-04	0.001204078
Pden_4033	*mreC*	rod shape-determining protein MreC	2.42	3.21E-05	7.09E-04
Pden_4034		putative rod shape-determining protein MreD	2.58	0.001302079	0.004990481
Pden_4035	*mrdA*	peptidoglycan glycosyltransferase	1.82	1.17E-04	0.001230568
Pden_4040		protein of unknown function DUF161	1.41	8.76E-04	0.003863525
Pden_4045	*chrA*	chromate transporter, chromate ion transporter (CHR) family	1.30	0.00218415	0.007167014
Pden_4047		NAD(P) transhydrogenase, beta subunit	-1.07	0.002573854	0.008034168
Pden_4048		NAD(P) transhydrogenase, alpha subunit	-1.16	0.001103107	0.004476884
Pden_4049		protein of unknown function DUF1498	-1.27	0.001675278	0.005952232
Pden_4050		Isoprenylcysteine carboxyl methyltransferase	2.97	2.23E-05	6.88E-04
Pden_4051	*glcB*	Malate synthase	1.97	7.31E-05	9.81E-04
Pden_4055	*proB*	glutamate 5-kinase	3.19	2.16E-04	0.001694513
Pden_4056	*obgE*	small GTP-binding protein	3.09	8.52E-04	0.003818535

Pden_4059		Endonuclease/exonuclease/phosphatase	-1.20	0.001201134	0.004728881
Pden_4061		conserved hypothetical protein	2.62	0.001976153	0.006701447
Pden_4062		2-isopropylmalate synthase	1.76	3.11E-04	0.002120136
Pden_4063	*cysS*	cysteinyl-tRNA synthetase	2.26	1.62E-04	0.001494676
Pden_4065		hypothetical protein	-1.50	2.20E-04	0.001708297
Pden_4071		NADPH-dependent FMN reductase	-2.90	0.003111747	0.009239266
Pden_4073	*dnaG*	DNA primase	2.33	5.92E-04	0.00305499
Pden_4074		transcriptional regulator, TetR family	2.96	1.90E-04	0.00159937
Pden_4078	*trmFO*	gid protein	2.95	1.59E-05	6.87E-04
Pden_4079	*gluQRS*	glutamyl-tRNA synthetase, class Ic	2.78	8.82E-05	0.001071072
Pden_4081		NifU-related protein involved in Fe-S cluster formation	2.09	3.78E-04	0.002311838
Pden_4083	*ligA*	DNA ligase, NAD-dependent	1.47	0.00321092	0.009462681
Pden_4093		hypothetical protein	2.65	2.47E-05	6.90E-04
Pden_4094		hypothetical protein	2.47	1.01E-04	0.001137092
Pden_4098	*mnmA*	tRNA (5-methylaminomethyl-2-thiouridylate)-methyltransferase	1.41	0.001120848	0.004521794
Pden_4099		Cysteine desulfuration protein SufE	1.54	6.79E-04	0.003306024
Pden_4101		GCN5-related N-acetyltransferase	2.25	8.38E-05	0.001045151
Pden_4110		HpcH/HpaI aldolase	3.74	2.72E-04	0.001970419
Pden_4111		conserved hypothetical protein	2.41	8.21E-05	0.001034
Pden_4112		L-carnitine dehydratase/bile acid-inducible protein F	2.61	4.07E-05	8.15E-04
Pden_4118		transcriptional regulator, LysR family	-2.06	7.04E-04	0.003368992
Pden_4123	*hflX*	GTP-binding protein HflX	1.67	2.77E-04	0.001988805
Pden_4130		signal transduction histidine kinase, nitrogen specific, NtrB	1.83	1.34E-04	0.001332887
Pden_4131	*dusB*	tRNA-U20-dihydrouridine synthase	2.14	0.001519188	0.005602074
Pden_4136		hypothetical protein	-1.70	9.56E-04	0.004096078
Pden_4140		periplasmic solute binding protein	2.36	1.74E-04	0.001512937
Pden_4141		hypothetical protein	-3.42	2.09E-04	0.001676926
Pden_4142		RNA-binding S4 domain protein	2.18	9.53E-05	0.001120076
Pden_4143	*tadA*	CMP/dCMP deaminase, zinc-binding protein	2.53	2.82E-05	7.03E-04
Pden_4144		pyruvate carboxylase	1.16	0.002215658	0.007228013
Pden_4146		transcriptional regulator, ArsR family	2.22	8.46E-04	0.003801247
Pden_4151	*soxY*	thiosulfate-binding protein SoxY	-1.88	0.002364635	0.007548642
Pden_4153	*soxA*	diheme cytochrome SoxA (sulfur oxidation)	-2.39	5.34E-04	0.002869505
Pden_4160		SoxH protein-like protein	-1.32	0.003041277	0.009096801
Pden_4165	*azoR*	(Acyl-carrier-protein) phosphodiesterase	-2.15	0.00124923	0.004865271
Pden_4166		NnrS family protein	2.47	5.17E-04	0.002804411
Pden_4167		ABC transporter related protein	3.37	2.68E-05	7.01E-04
Pden_4168		binding-protein-dependent transport systems inner membrane component	3.07	4.18E-04	0.002469493
Pden_4169		putative sulfonate/nitrate transport system substrate-binding protein	2.04	0.001071706	0.00441142
Pden_4170		hypothetical protein	2.25	4.02E-05	8.15E-04
Pden_4171		conserved hypothetical protein	2.52	3.73E-04	0.002309828
Pden_4172		transcriptional regulator, BadM/Rrf2 family	2.30	9.31E-04	0.004030546
Pden_4173		TonB-dependent receptor	1.92	0.001164643	0.00464298
Pden_4174		transcriptional regulator, DeoR family	1.16	0.001390664	0.0052336
Pden_4178		mannitol ABC transporter ATP-binding protein / sorbitol ABC transporter ATP-binding protein	-1.73	0.001395511	0.005245341
Pden_4179		sorbitol ABC transporter membrane protein / mannitol ABC transporter membrane protein	-2.09	3.11E-04	0.002120136
Pden_4180		mannitol ABC transporter membrane protein / sorbitol ABC transporter membrane protein	-2.13	0.002189361	0.007174243
Pden_4182	*queF*	GTP cyclohydrolase I	1.80	0.002140403	0.007069055
Pden_4183	*queE*	preQ(0) biosynthesis protein QueE	3.40	1.96E-05	6.87E-04
Pden_4184		6-pyruvoyl tetrahydropterin synthase and hypothetical protein	3.38	8.37E-06	6.87E-04
Pden_4185	*queC*	exsB protein	3.63	4.79E-05	8.86E-04
Pden_4186	*ligD*	conserved hypothetical protein	2.64	5.85E-05	9.13E-04
Pden_4187	*ugpC*	carbohydrate ABC transporter ATP-binding protein, CUT1 family	-1.98	1.88E-04	0.001592668
Pden_4188	*ugpE*	carbohydrate ABC transporter membrane protein 2, CUT1 family	-3.35	2.87E-04	0.002014555
Pden_4189	*ugpA*	carbohydrate ABC transporter membrane protein 1, CUT1 family	-3.56	8.70E-06	6.87E-04
Pden_4190	*ugpB*	carbohydrate ABC transporter substrate-binding protein, CUT1 family	-3.13	6.44E-06	6.87E-04
Pden_4191		hypothetical protein	2.34	1.64E-04	0.001498242
Pden_4192		hypothetical protein	2.54	3.08E-04	0.002109645
Pden_4196		hypothetical protein	2.61	0.001013213	0.004250121
Pden_4202		hemin-degrading family protein	-3.26	2.27E-04	0.001730572
Pden_4210		response regulator receiver protein	-2.41	8.41E-04	0.003795331
Pden_4211		SSS sodium solute transporter superfamily	-3.42	2.26E-04	0.001729094
Pden_4213	*acs*	acetyl-coenzyme A synthetase	-2.16	5.29E-05	9.01E-04
Pden_4217		ABC transporter related protein	1.84	0.001152638	0.00461259
Pden_4223		conserved hypothetical protein	1.67	5.10E-04	0.00278709
Pden_4227		conserved hypothetical protein	2.23	0.003004582	0.009006631
Pden_4231		conserved hypothetical protein	1.38	0.002893516	0.008751417
Pden_4232		PpiC-type peptidyl-prolyl cis-trans isomerase	1.25	8.23E-04	0.003744016
Pden_4233	*narI*	respiratory nitrate reductase gamma subunit	1.38	5.09E-04	0.002784714
Pden_4234	*narJ*	respiratory nitrate reductase chaperone NarJ	-1.11	0.00268689	0.008277841
Pden_4241		drug resistance transporter, Bcr/CflA subfamily	3.05	0.001293285	0.00497183
Pden_4243		GntR domain protein	1.39	4.64E-04	0.002633628
Pden_4249		sodium:dicarboxylate symporter	-1.26	0.002606089	0.008101389
Pden_4254		protein of unknown function DUF81	3.78	1.71E-04	0.001509512
Pden_4255		molecular chaperone, DnaK	5.07	2.40E-05	6.88E-04
Pden_4257	*xdhA*	molybdopterin dehydrogenase, FAD-binding protein	-2.34	0.002169528	0.00714195
Pden_4258	*xdhB*	xanthine dehydrogenase, molybdenum binding subunit apoprotein / Xanthine oxidase	-2.81	8.59E-04	0.003825716
Pden_4259	*xdhC*	molybdenum cofactor sulfurylase	-2.12	6.25E-04	0.003147257
Pden_4260	*guaD*	guanine deaminase	-2.26	0.001580048	0.005746971
Pden_4261		acetone carboxylase, gamma subunit	-6.15	7.53E-04	0.003532514
Pden_4262		acetone carboxylase, alpha subunit	-7.07	6.79E-04	0.003306024
Pden_4263		acetone carboxylase, beta subunit	-6.41	5.04E-04	0.002762958
Pden_4270		amino acid/amide ABC transporter membrane protein 2, HAAT family	-4.72	0.002998236	0.00899293
Pden_4271		amino acid/amide ABC transporter membrane protein 1, HAAT family	-5.25	0.002219671	0.007231971
Pden_4272		amino acid/amide ABC transporter substrate-binding protein, HAAT family	-5.94	0.001195529	0.004714145
Pden_4273	*tpa*	aminotransferase	-5.22	1.60E-04	0.001489115
Pden_4274		transcriptional regulator, GntR family	-2.80	5.98E-04	0.003074387

Pden_4275	*pta*	phosphotransacetylase	2.37	9.24E-04	0.004015527
Pden_4276		conserved hypothetical protein 698	2.08	0.00104852	0.004358501
Pden_4279		major facilitator superfamily MFS_1	2.13	0.002136688	0.007066005
Pden_4281		oligopeptide/dipeptide ABC transporter, ATPase subunit	2.34	4.00E-04	0.002394246
Pden_4289		conserved hypothetical protein	2.27	0.003218424	0.009468264
Pden_4291	*dnaE2*	error-prone DNA polymerase, DnaE-like protein	1.44	9.46E-04	0.004070411
Pden_4293		transcriptional regulator, HxlR family	1.64	0.001812206	0.006293002
Pden_4301	*acnA*	aconitase	-2.29	0.001546117	0.005660151
Pden_4303	*rpsD*	SSU ribosomal protein S4P	2.22	0.002229754	0.007255299
Pden_4305	*tpiA*	triosephosphate isomerase	1.34	0.001659612	0.005923996
Pden_4306	*metN*	ABC transporter related protein	2.12	0.001071673	0.00441142
Pden_4309		Acetyl-CoA hydrolase	1.14	0.001882291	0.006469312
Pden_4312		FAD linked oxidase domain protein	1.77	0.001508609	0.005578741
Pden_4313		GCN5-related N-acetyltransferase	2.43	8.80E-04	0.003878366
Pden_4314		peptidase M16 domain protein	1.30	0.001558081	0.005691604
Pden_4322	*tldD*	microcin-processing peptidase 2, Unknown type peptidase, MEROPS family U62	1.69	0.003118686	0.009239266
Pden_4323	*dprA*	DNA protecting protein DprA	3.48	1.39E-05	6.87E-04
Pden_4329	*pstC*	phosphate ABC transporter membrane protein 1, PhoT family	1.79	3.98E-04	0.002393936
Pden_4330		phosphate ABC transporter substrate-binding protein, PhoT family	3.26	4.22E-06	6.87E-04
Pden_4331		hypothetical protein	2.39	0.001776789	0.006186991
Pden_4333		hypothetical protein	2.64	8.48E-04	0.003806599
Pden_4334		amino acid/polyamine/organocation transporter, APC superfamily	-2.70	1.52E-04	0.001450721
Pden_4335		type I secretion membrane fusion protein, HlyD family	3.21	6.36E-05	9.35E-04
Pden_4336		type I secretion system ATPase	2.95	2.16E-05	6.88E-04
Pden_4337		conserved hypothetical protein	2.88	5.43E-05	9.01E-04
Pden_4345		secretion protein HlyD family protein	2.98	2.60E-05	7.00E-04
Pden_4346		colicin V processing peptidase, Cysteine peptidase, MEROPS family C39	3.15	3.73E-04	0.002309828
Pden_4350		periplasmic glucan biosynthesis protein, MdoG	-1.08	0.001175238	0.004659024
Pden_4351		permease for cytosine/purines, uracil, thiamine, allantoin	1.46	6.83E-04	0.00331005
Pden_4352		5-oxoprolinase (ATP-hydrolyzing)	-2.23	4.38E-05	8.44E-04
Pden_4353		5-oxoprolinase (ATP-hydrolyzing)	-1.44	0.00120508	0.004740731
Pden_4364		ThiJ/PfpI domain protein	3.14	2.06E-04	0.001670021
Pden_4365		transcriptional regulator, AraC family with amidase-like domain	2.11	8.63E-04	0.003825716
Pden_4366		DEAD/DEAH box helicase domain protein	4.89	1.17E-06	6.87E-04
Pden_4368		sodium:dicarboxylate symporter	-4.38	1.66E-04	0.001500534
Pden_4370		conserved hypothetical protein 698	4.15	0.001520498	0.005602824
Pden_4371		conserved hypothetical protein	2.42	5.27E-04	0.002843404
Pden_4373		TonB-dependent siderophore receptor	-1.51	5.38E-04	0.002874906
Pden_4376	*nrdI*	NrdI protein	-1.38	0.002932109	0.00883647
Pden_4379		Bile acid:sodium symporter	2.65	0.003356376	0.009771899
Pden_4380		putative transcriptional regulator, GntR family	1.32	0.00223227	0.007258821
Pden_4381		TrkA-C domain protein	3.07	7.98E-05	0.001019694
Pden_4382		TonB-dependent receptor	1.94	6.58E-05	9.47E-04
Pden_4383		periplasmic binding protein	2.65	4.75E-05	8.86E-04
Pden_4384		transport system permease protein	3.11	3.79E-04	0.002312901
Pden_4385		ABC transporter related protein	3.07	1.73E-04	0.001509512
Pden_4387		carbohydrate ABC transporter substrate-binding protein, CUT1 family	-2.32	1.52E-04	0.001450721
Pden_4393	*glpD*	homodimeric glycerol 3-phosphate dehydrogenase (quinone)	1.63	2.39E-04	0.001791022
Pden_4397	*glcF*	protein of unknown function DUF224, cysteine-rich region domain protein	-3.03	3.00E-05	7.09E-04
Pden_4398		FAD linked oxidase domain protein	-2.08	0.002816558	0.00856985
Pden_4401		Uncharacterized protein UPF0065	-5.80	9.11E-05	0.001090823
Pden_4402		protein of unknown function DUF112, transmembrane	-5.52	1.46E-04	0.001416129
Pden_4403		protein of unknown function DUF1468	-5.33	1.39E-04	0.00136816
Pden_4404		Uncharacterized protein UPF0065	-5.19	3.85E-04	0.00233751
Pden_4417	*lepA*	GTP-binding protein LepA	2.03	3.31E-05	7.22E-04
Pden_4418		protein of unknown function DUF6, transmembrane	2.63	0.00103233	0.004323147
Pden_4421		phosphonate metabolism protein PhnM	1.73	4.00E-04	0.002394246
Pden_4422		alkylhydroperoxidase like protein, AhpD family	2.72	6.73E-05	9.57E-04
Pden_4423		phosphoglucomutase/phosphomannomutase alpha/beta/alpha domain I	-1.39	0.003242166	0.009521511
Pden_4427	*glgC*	glucose-1-phosphate adenylyltransferase	-1.95	1.00E-04	0.001137092
Pden_4430	*nadC*	nicotinate-nucleotide pyrophosphorylase (carboxylating)	1.29	4.77E-04	0.002686491
Pden_4431		L-aspartate oxidase	1.79	0.001435091	0.005357684
Pden_4434		putative aminotransferase, class IV	2.69	5.50E-04	0.002920173
Pden_4435		aminodeoxychorismate synthase, subunit I	2.94	8.05E-04	0.003682344
Pden_4442		carbohydrate ABC transporter substrate-binding protein, CUT1 family	2.84	0.002062811	0.006893739
Pden_4447		extracellular solute-binding protein, family 1	-4.27	1.40E-05	6.87E-04
Pden_4449		assimilatory nitrate reductase (NADH) alpha subunit apoprotein	2.34	0.003405643	0.009867016
Pden_4452		assimilatory nitrite reductase (NAD(P)H) large subunit precursor	2.75	4.28E-05	8.32E-04
Pden_4453		major facilitator superfamily MFS_1	3.25	5.10E-05	9.00E-04
Pden_4454		putative nitrate transport protein	2.90	3.53E-04	0.00226395
Pden_4455		response regulator receiver and ANTAR domain protein	3.31	1.58E-05	6.87E-04
Pden_4458		RNA binding S1 domain protein	2.47	2.99E-05	7.09E-04
Pden_4459		hypothetical protein	3.10	0.001963935	0.006668949
Pden_4461		nitrogen regulatory protein P-II	-1.41	9.48E-04	0.004070411
Pden_4462	*glnA*	L-glutamine synthetase	-2.87	0.002703924	0.008311152
Pden_4463		Adenylosuccinate lyase	1.74	2.73E-04	0.001974225
Pden_4464		flagellar motor switch protein FliG	3.96	1.63E-04	0.001496408
Pden_4469		replicative DNA helicase	1.68	0.001644619	0.005892926
Pden_4479		methylmalonate-semialdehyde dehydrogenase (acylating)	-2.50	7.81E-04	0.00362655
Pden_4480		acyl-CoA dehydrogenase domain protein	-2.41	1.78E-05	6.87E-04
Pden_4481		Enoyl-CoA hydratase/isomerase	-2.22	3.81E-04	0.002323045
Pden_4482	*mmsB*	3-hydroxyisobutyrate dehydrogenase	-2.29	1.26E-04	0.001288234
Pden_4484	*recN*	DNA replication and repair protein RecN	2.42	5.11E-04	0.002788261
Pden_4487	*ftsZ*	cell division protein FtsZ	-1.54	6.64E-04	0.003261511
Pden_4491	*murB*	UDP-N-acetylmuramate dehydrogenase	2.49	2.39E-04	0.001790755
Pden_4502		ABC polyamine transporter, periplasmic substrate-binding protein	-2.83	9.69E-06	6.87E-04
Pden_4503	*proA*	glutamate-5-semialdehyde dehydrogenase	2.56	3.28E-05	7.19E-04

Pden_4506		ornithine-acyl[acyl carrier protein] N-acyltransferase	1.89	4.53E-04	0.002599236
Pden_4507		lyso-ornithine lipid acyltransferase	2.00	0.002082582	0.006950636
Pden_4509		conserved hypothetical protein	1.47	5.03E-04	0.002762958
Pden_4510	*ubiB*	2-octaprenylphenol hydroxylase	1.79	5.74E-05	9.13E-04
Pden_4511	*ubiE*	2-octaprenyl-6-methoxy-1,4-benzoquinone methylase	1.38	4.94E-04	0.002738506
Pden_4515		3-demethylubiquinone-9 3-methyltransferase	-2.69	4.95E-04	0.002741428
Pden_4516		3-demethylubiquinone-9 3-methyltransferase	-1.61	4.21E-04	0.002481719
Pden_4517		transcriptional regulator, MarR family	2.24	0.001713949	0.006047193
Pden_4518		regulatory protein, ArsR	3.10	4.52E-05	8.61E-04
Pden_4522		Glyoxalase/bleomycin resistance protein/dioxygenase	3.23	3.64E-06	6.87E-04
Pden_4523		putative transcriptional regulator, MerR family	1.70	1.41E-04	0.001376063
Pden_4524		hypothetical protein	2.69	5.53E-05	9.01E-04
Pden_4525		transcriptional regulator, MarR family	3.20	4.05E-04	0.002417316
Pden_4526		Methyltransferase type 11	2.00	8.61E-05	0.001060694
Pden_4527		transcriptional regulator, MarR family	2.31	3.16E-05	7.09E-04
Pden_4533		RNA polymerase, sigma subunit, ECF family	4.26	1.68E-04	0.00150607
Pden_4534		putative FecR	3.98	6.25E-04	0.003147257
Pden_4535		TonB-dependent siderophore receptor	3.11	5.94E-06	6.87E-04
Pden_4541		hypothetical protein	2.59	2.56E-05	7.00E-04
Pden_4542		transcriptional regulator, RpiR family	1.87	2.44E-04	0.001819959
Pden_4545		TRAP C4-dicarboxylate transport system permease DctM subunit	-1.62	4.63E-04	0.002631196
Pden_4546	*dctP*	TRAP dicarboxylate transporter- DctP subunit	-3.58	0.001037435	0.00432914
Pden_4547		L-glutamine synthetase	-2.72	2.67E-04	0.001946418
Pden_4549		iron-containing alcohol dehydrogenase	-2.00	9.56E-04	0.004096078
Pden_4550	*acs*	acetyl-coenzyme A synthetase	-2.15	5.76E-05	9.13E-04
Pden_4551		conserved hypothetical protein	-5.76	0.002241435	0.007272039
Pden_4552		Na+/solute symporter	-4.15	1.72E-05	6.87E-04
Pden_4553		cyclic nucleotide-binding protein	-3.70	2.76E-05	7.03E-04
Pden_4558		RNA polymerase, sigma-24 subunit, ECF subfamily	2.03	7.39E-05	9.86E-04
Pden_4560		sodium/sulphate symporter	3.74	0.001707847	0.006035961
Pden_4561		Glycerate kinase	2.85	3.69E-04	0.002309828
Pden_4562		Hemolysin-type calcium-binding region	1.11	0.001164572	0.00464298
Pden_4563		transcriptional regulator, LysR family	1.69	3.14E-04	0.00213146
Pden_4566		protein of unknown function DUF6, transmembrane	4.15	9.46E-07	6.87E-04
Pden_4568		hypothetical protein	3.37	9.34E-06	6.87E-04
Pden_4569		hypothetical protein	3.21	1.72E-05	6.87E-04
Pden_4570		hypothetical protein	1.52	0.001150466	0.004608236
Pden_4572		major facilitator superfamily MFS_1	2.44	1.85E-04	0.001572457
Pden_4573		protein of unknown function DUF6, transmembrane	2.25	2.98E-05	7.09E-04
Pden_4574		aldo/keto reductase	1.41	0.00284294	0.008639738
Pden_4575		transcriptional regulator, LysR family	1.97	0.003312038	0.009698582
Pden_4578		Cupin 2, conserved barrel domain protein	2.66	4.26E-05	8.32E-04
Pden_4579		transcriptional regulator, HxlR family	3.24	5.36E-04	0.002869505
Pden_4581		transcriptional regulator, LysR family	2.16	0.001762835	0.006155335
Pden_4582		conserved hypothetical protein	1.54	4.36E-04	0.002545539
Pden_4584		short-chain dehydrogenase/reductase SDR	2.03	9.62E-04	0.004108812
Pden_4588		putative molecular chaperone small heat shock protein, HSP20 family	2.16	5.42E-04	0.002889217
Pden_4589		hypothetical protein	3.23	6.83E-05	9.60E-04
Pden_4591		Rhodanese domain protein	-1.32	5.11E-04	0.002788261
Pden_4592		uncharacterized peroxidase-related enzyme	-2.50	3.44E-04	0.002238614
Pden_4594		thiamine pyrophosphate enzyme domain protein TPP-binding protein	-1.08	0.002010128	0.006788563
Pden_4599		putative CoA transferase, subunit B	1.20	0.003412287	0.009872234
Pden_4603		putative ABC transporter binding protein component	-2.32	0.001067049	0.004402992
Pden_4605		conserved hypothetical protein	2.22	0.002215414	0.007228013
Pden_4606		Radical SAM domain protein	2.43	6.23E-05	9.34E-04
Pden_4607		Uracil-DNA glycosylase superfamily	3.47	2.05E-05	6.87E-04
Pden_4608		putative general stress protein 26	1.83	1.32E-04	0.001321016
Pden_4609		phospholipase D/Transphosphatidylase	3.33	2.48E-05	6.90E-04
Pden_4610		FAD dependent oxidoreductase	2.75	0.00110356	0.004476884
Pden_4611		Catalase	1.88	5.38E-04	0.002874906
Pden_4612		Peptidoglycan-binding domain 1 protein	1.92	4.05E-04	0.002417316
Pden_4613		conserved hypothetical protein	4.54	3.37E-04	0.002227917
Pden_4615		esterase, PHB depolymerase family	1.68	2.33E-04	0.001755773
Pden_4616	*hydA*	dihydropyrimidinase	-1.62	6.08E-04	0.003101533
Pden_4624		transcriptional regulator, AsnC family	1.50	4.31E-04	0.002528783
Pden_4627		hypothetical protein	3.86	0.00333821	0.009735806
Pden_4628		D-amino-acid dehydrogenase	1.95	2.96E-04	0.002049708
Pden_4629		peptidase S9, prolyl oligopeptidase active site domain protein	2.21	3.86E-05	7.97E-04
Pden_4630		transcriptional regulator, TetR family	1.32	4.34E-04	0.002539593
Pden_4631		major facilitator superfamily MFS_1	1.18	0.001442857	0.005379369
Pden_4632		beta-lactamase	2.10	0.002059529	0.006887314
Pden_4633		Uncharacterized conserved protein UCP012702	2.45	1.26E-04	0.001288342
Pden_4634		Leucyl aminopeptidase	2.24	8.32E-04	0.003768614
Pden_4635		peptidase M42 family protein	2.03	7.44E-05	9.87E-04
Pden_4637		ornithine cyclodeaminase	2.62	1.70E-04	0.001509512
Pden_4638		hypothetical protein	3.26	8.29E-06	6.87E-04
Pden_4641		conserved hypothetical protein	-2.02	0.002773026	0.00848841
Pden_4642		conserved hypothetical protein	-2.39	8.04E-05	0.001023289
Pden_4649		extracellular solute-binding protein, family 1	-1.49	3.59E-04	0.002286436
Pden_4675		transcriptional regulator, RpiR family	2.14	6.04E-05	9.27E-04
Pden_4677		2OG-Fe(II) oxygenase	-1.97	2.17E-04	0.00169785
Pden_4685		secretion protein HlyD family protein	2.27	3.20E-05	7.09E-04
Pden_4686	*rbbA*	ABC transporter related protein	2.57	5.17E-05	9.01E-04
Pden_4688		YbaK/prolyl-tRNA synthetase associated region	3.13	2.85E-05	7.03E-04
Pden_4689		hypothetical protein	2.35	1.36E-04	0.001341221
Pden_4690		phosphatidyl-N-methylethanolamine N-methyltransferase	1.60	2.75E-04	0.001983635
Pden_4691	*pssA*	CDP-diacylglycerol--serine O-phosphatidyltransferase	1.78	1.03E-04	0.001157656

Pden_4692	*psd*	phosphatidylserine decarboxylase related protein	2.23	3.49E-04	0.002245802
Pden_4693		uracil-xanthine permease	2.76	2.26E-05	6.88E-04
Pden_4696		acriflavin resistance protein	1.90	2.10E-04	0.001676926
Pden_4700		cytochrome c, class II	1.62	0.002520892	0.007932429
Pden_4709		conserved hypothetical protein	2.35	4.99E-04	0.002752317
Pden_4713	*minC*	septum site-determining protein MinC	2.32	0.001588279	0.005772762
Pden_4714		phosphoribosyltransferase	3.85	1.12E-04	0.001190496
Pden_4723		periplasmic nitrate reductase subunit NapC	-1.81	0.001787674	0.006220615
Pden_4725		conserved hypothetical protein	4.04	2.18E-05	6.88E-04
Pden_4726		MscS Mechanosensitive ion channel	5.21	9.02E-06	6.87E-04
Pden_4728	*mauR*	transcriptional regulator, LysR family	3.79	5.57E-05	9.01E-04
Pden_4729	*mauF*	conserved hypothetical protein	2.01	8.87E-04	0.003893864
Pden_4736	*mauG*	Di-heme cytochrome c peroxidase	2.13	1.65E-04	0.001498242
Pden_4737	*mauM*	periplasmic nitrate reductase subunit NapG	3.11	1.88E-05	6.87E-04
Pden_4738	*mauN*	periplasmic nitrate reductase subunit NapH	3.14	3.68E-05	7.74E-04
Pden_4742		conserved hypothetical protein	1.40	4.78E-04	0.002686576
Pden_4743		Peptidoglycan-binding domain 1 protein	2.99	0.003041849	0.009096801
Pden_4746		binding-protein-dependent transport systems inner membrane component	1.66	0.001276463	0.004938294
Pden_4752		aldehyde dehydrogenase	-1.50	0.001381861	0.005219358
Pden_4753		dihydrodipicolinate synthetase	-1.47	3.75E-04	0.002309828
Pden_4754		conserved hypothetical protein	3.60	9.05E-06	6.87E-04
Pden_4755		putative carbohydrate binding protein	2.78	8.31E-06	6.87E-04
Pden_4762		conserved hypothetical protein	2.65	4.46E-04	0.002570923
Pden_4763		two component, sigma54 specific, transcriptional regulator, Fis family	1.47	0.002660957	0.008227777
Pden_4772		RNA polymerase, sigma-24 subunit, ECF subfamily	1.55	0.003395161	0.009856478
Pden_4775		protein of unknown function DUF1045	1.44	0.001255229	0.004884876
Pden_4782	*phnG*	phosphonate metabolism PhnG	1.76	4.47E-04	0.002574525
Pden_4783	*phnF*	transcriptional regulator, GntR family	3.42	4.34E-04	0.002539593
Pden_4784	*phnE*	phosphonate ABC transporter, inner membrane subunit	3.96	1.74E-05	6.87E-04
Pden_4785	*phnE*	phosphonate ABC transporter, inner membrane subunit	4.23	6.86E-04	0.00331005
Pden_4786	*phnD*	phosphonate ABC transporter, periplasmic phosphonate-binding protein	4.06	1.25E-05	6.87E-04
Pden_4787	*phnC*	phosphonate ABC transporter, ATPase subunit	4.88	2.87E-05	7.03E-04
Pden_4788		hypothetical protein	2.11	9.18E-04	0.003994968
Pden_4789		NADPH-dependent FMN reductase	2.05	0.001509064	0.005578741
Pden_4793		amino acid/amide ABC transporter ATP-binding protein 2, HAAT family	-3.27	2.36E-05	6.88E-04
Pden_4794		amino acid/amide ABC transporter ATP-binding protein 1, HAAT family	-3.52	3.09E-05	7.09E-04
Pden_4795		amino acid/amide ABC transporter membrane protein 2, HAAT family	-4.30	9.30E-06	6.87E-04
Pden_4796		amino acid/amide ABC transporter membrane protein 1, HAAT family	-5.15	5.47E-05	9.01E-04
Pden_4797		amino acid/amide ABC transporter substrate-binding protein, HAAT family	-5.29	5.58E-05	9.01E-04
Pden_4798	*paaN*	phenylacetic acid degradation protein paaN	-1.81	3.42E-04	0.002238614
Pden_4799	*paaB*	Enoyl-CoA hydratase / short chain enoyl-CoA hydratase	-2.10	7.14E-04	0.00340186
Pden_4800	*paaI*	phenylacetic acid degradation protein PaaD	-4.35	0.001495407	0.005538586
Pden_4801	*paaF*	phenylacetate-CoA ligase	-4.58	2.86E-05	7.03E-04
Pden_4803		adenylyltransferase	1.37	0.001383075	0.005219358
Pden_4804	*paaZ*	phenylacetic acid degradation protein paaN	-1.45	0.003047962	0.009099135
Pden_4805		phenylacetic acid catabolic family protein	-1.79	9.59E-04	0.004097841
Pden_4806	*paaK*	phenylacetate-CoA oxygenase/reductase, PaaK subunit	-2.07	2.98E-04	0.002053033
Pden_4807	*paaJ*	phenylacetate-CoA oxygenase, PaaJ subunit	-2.23	1.31E-04	0.001314735
Pden_4809	*paaB*	phenylacetate-CoA oxygenase, PaaH subunit	-2.85	0.001173841	0.004659024
Pden_4810	*paaA*	phenylacetate-CoA oxygenase, PaaG subunit	-2.32	9.09E-05	0.001090128
Pden_4813		Penicillin amidase	-2.76	2.20E-04	0.001708297
Pden_4814		conserved hypothetical protein	-2.58	6.29E-05	9.34E-04
Pden_4815		conserved hypothetical protein	-2.10	6.82E-05	9.60E-04
Pden_4816		TRAP C4-dicarboxylate transport system permease DctM subunit	-2.08	5.66E-04	0.00297487
Pden_4817		TRAP dicarboxylate transporter, DctP subunit	-3.73	1.11E-05	6.87E-04
Pden_4818		transcriptional regulator, IclR family	-2.75	0.00103422	0.004323912
Pden_4819		Thiolase	-2.79	1.27E-04	0.001289642
Pden_4820		AMP-dependent synthetase and ligase	-2.03	0.001070605	0.00441142
Pden_4821		conserved hypothetical protein	-1.77	0.002438271	0.00772043
Pden_4822		short-chain dehydrogenase/reductase SDR	-2.33	9.95E-04	0.004206003
Pden_4825	*ftsH*	membrane protease FtsH catalytic subunit	2.55	4.27E-05	8.32E-04
Pden_4827		TonB-dependent siderophore receptor	2.08	0.002543326	0.007983175
Pden_4828		ABC transporter related protein	3.04	5.16E-04	0.002802086
Pden_4829		periplasmic binding protein	2.71	1.73E-04	0.001509512
Pden_4830		transport system permease protein	2.85	6.01E-04	0.003082284
Pden_4832		negative transcriptional regulator	1.74	0.00318949	0.009410482
Pden_4833		transcriptional regulator, GntR family	1.62	2.83E-04	0.002009098
Pden_4834		transcriptional regulator, AraC family	2.19	8.55E-04	0.003825716
Pden_4835		major facilitator superfamily MFS_1	2.99	9.99E-05	0.001137092
Pden_4837		protein of unknown function DUF6, transmembrane	2.81	1.64E-04	0.001498242
Pden_4838		conserved hypothetical protein	3.12	1.59E-04	0.001489115
Pden_4848		PfkB domain protein	2.16	2.96E-04	0.002049708
Pden_4850		monosaccharide ABC transporter ATP-binding protein, CUT2 family	2.17	0.00314761	0.009308616
Pden_4852		peptidase C14, caspase catalytic subunit p20	3.31	1.29E-04	0.001309795
Pden_4854		predicted aconitase subunit 1	2.22	3.44E-04	0.002238614
Pden_4859	*hypF*	proline racemase	-1.39	0.00307117	0.009162836
Pden_4862		amino acid ABC transporter membrane protein 2, PAAT family	-1.69	1.68E-04	0.001505274
Pden_4863		amino acid ABC transporter membrane protein 1, PAAT family	-2.76	3.32E-04	0.002212274
Pden_4864		amino acid ABC transporter substrate-binding protein, PAAT family	-2.45	1.57E-05	6.87E-04
Pden_4868		transcriptional regulator, GntR family	-1.20	0.002884602	0.008739141
Pden_4871	*proC*	pyrroline-5-carboxylate reductase	3.37	4.38E-04	0.002548142
Pden_4872		FAD linked oxidase domain protein	2.78	1.09E-04	0.001182514
Pden_4876		dihydrodipicolinate synthetase	1.84	0.001756021	0.006137651
Pden_4877		L-lactate dehydrogenase (cytochrome)	3.12	7.80E-04	0.003624521
Pden_4878		hydroxymethylglutaryl-CoA lyase	2.64	5.45E-05	9.01E-04
Pden_4879		L-carnitine dehydratase/bile acid-inducible protein F	2.54	3.19E-05	7.09E-04
Pden_4882		binding-protein-dependent transport systems inner membrane component	3.99	6.21E-06	6.87E-04

Pden_4883		ABC transporter related protein	5.15	1.63E-04	0.001495531
Pden_4890		acyl-CoA dehydrogenase domain protein	1.55	7.92E-04	0.003644887
Pden_4892		MmgE/PrpD family protein	1.72	9.37E-04	0.004043846
Pden_4894		FAD dependent oxidoreductase	1.69	3.11E-04	0.002120675
Pden_4895		BFD domain protein (2Fe-2S)-binding domain protein	2.64	1.34E-04	0.001332604
Pden_4896		oxidoreductase FAD/NAD(P)-binding domain protein	1.95	3.25E-04	0.002189096
Pden_4900		conserved hypothetical protein	1.31	0.001239463	0.004844257
Pden_4901		luciferase family protein	1.91	0.001246164	0.004857065
Pden_4902		ABC transporter related protein	2.85	7.17E-06	6.87E-04
Pden_4903		binding-protein-dependent transport systems inner membrane component	2.16	3.60E-05	7.62E-04
Pden_4906		Sarcosine oxidase, gamma subunit	1.36	0.00179523	0.006242616
Pden_4907		sarcosine oxidase, alpha subunit family	1.30	0.002687887	0.008277841
Pden_4910		transcriptional regulator, AraC family with amidase-like domain	1.69	0.001896485	0.006500951
Pden_4911		extracellular solute-binding protein	-2.33	0.002022886	0.006814284
Pden_4918		hypothetical protein	3.22	0.001622733	0.005840986
Pden_4919	*map*	methionine aminopeptidase, type I	1.40	5.80E-04	0.003020791
Pden_4920		hypothetical protein	2.54	7.96E-05	0.001019694
Pden_4921		alpha/beta hydrolase fold protein	1.39	4.60E-04	0.002626498
Pden_4922		conserved hypothetical protein	2.93	3.61E-05	7.62E-04
Pden_4923		transcriptional regulator, IclR family	1.35	0.002569375	0.00802847
Pden_4929		2-keto-3-deoxygalactonate kinase	1.44	0.001200498	0.004728881
Pden_4930		2-keto-3-deoxy-phosphogalactonate aldolase	1.76	3.38E-04	0.002227917
Pden_4932		short-chain dehydrogenase/reductase SDR	1.71	5.89E-04	0.003052963
Pden_4933		conserved hypothetical protein	2.60	6.59E-04	0.003246886
Pden_4934		BFD domain protein (2Fe-2S)-binding domain protein	2.71	4.37E-04	0.002545539
Pden_4935		hypothetical protein	1.86	2.34E-04	0.001760831
Pden_4941		Betaine-aldehyde dehydrogenase	-1.74	4.39E-04	0.00254936
Pden_4942		thiamine pyrophosphate enzyme domain protein TPP-binding protein	-1.57	3.58E-04	0.002285725
Pden_4943		extracellular solute-binding protein, family 1	-3.00	5.50E-04	0.002920173
Pden_4947		protein of unknown function DUF1127	2.09	0.002517515	0.007926729
Pden_4948		hypothetical protein	1.68	5.13E-04	0.002791657
Pden_4951		NADH:flavin oxidoreductase/NADH oxidase	2.10	2.65E-05	7.01E-04
Pden_4956		FAD dependent oxidoreductase	2.06	0.00278022	0.008505288
Pden_4987		RNA polymerase, sigma 54 subunit, RpoN/SigL	2.05	3.11E-04	0.002120136
Pden_4988		protein of unknown function UPF0118	1.19	0.00205509	0.006885889
Pden_4996		CoA-binding domain protein	-1.75	0.001387948	0.005229852
Pden_4997		amino acid/amide ABC transporter ATP-binding protein 2, HAAT family	-2.15	8.96E-04	0.003925308
Pden_4999		amino acid/amide ABC transporter membrane protein 2, HAAT family	-2.21	5.36E-04	0.002869505
Pden_5000		amino acid/amide ABC transporter membrane protein 1, HAAT family	-1.96	2.21E-04	0.001709064
Pden_5001		HupE/UreJ protein	1.86	3.81E-04	0.002323045
Pden_5002		Rhodanese domain protein	2.12	4.16E-05	8.27E-04
Pden_5003		Cupin 2, conserved barrel domain protein	1.81	0.001717306	0.006050604
Pden_5004	*ssuB2*	ABC transporter related protein	2.75	7.64E-05	0.001002704
Pden_5005		binding-protein-dependent transport systems inner membrane component	2.91	2.30E-04	0.001743438
Pden_5006		aliphatic sulfonates family ABC transporter, periplasmic ligand-binding protein	2.56	3.72E-05	7.78E-04
Pden_5007		monooxygenase, NtaA/SnaA/SoxA family	3.03	1.95E-05	6.87E-04
Pden_5008		copper resistance protein CopC	4.80	0.001363237	0.005166067
Pden_5009		nuclear export factor GLE1	4.59	0.002671695	0.008248045
Pden_5010		ABC transporter related protein	3.51	5.52E-04	0.002925581
Pden_5011		binding-protein-dependent transport systems inner membrane component	2.83	4.30E-05	8.34E-04
Pden_5012		ABC transporter substrate-binding protein	3.19	3.01E-04	0.002073566
Pden_5013		Alkanesulfonate monooxygenase	2.79	7.70E-05	0.001002704
Pden_5014		Acyl-CoA dehydrogenase, type 2, C-terminal domain	3.63	1.75E-04	0.001517116
Pden_5015		hypothetical protein	3.70	5.94E-05	9.17E-04
Pden_5016		serine O-acetyltransferase	3.37	6.64E-05	9.49E-04
Pden_5017		conserved hypothetical protein	3.20	1.27E-05	6.87E-04
Pden_5018		Hydroxypyruvate isomerase	2.04	3.33E-04	0.002219207
Pden_5019		transcriptional regulator, IclR family	1.75	6.09E-04	0.003105221
Pden_5021		3-oxoadipate CoA-transferase beta subunit	2.19	1.57E-04	0.00147988
Pden_5022	*pcaF*	3-oxoadipyl-CoA thiolase	2.16	7.32E-05	9.81E-04
Pden_5023		NAD-dependent epimerase/dehydratase	2.83	1.75E-05	6.87E-04
Pden_5024		hypothetical protein	3.50	6.50E-05	9.41E-04
Pden_5026		hypothetical protein	2.23	8.86E-05	0.001071179
Pden_5030		hypothetical protein	1.80	1.72E-04	0.001509512
Pden_5034		initiator RepB protein	2.44	1.73E-05	6.87E-04
Pden_5037		ABC transporter related protein	1.85	7.79E-04	0.003619497
Pden_5039		ABC-2 type transporter	1.66	0.00177002	0.006171016
Pden_5040		glycosyl transferase, group 1	1.29	8.22E-04	0.003744016
Pden_5041		hypothetical protein	3.55	6.31E-06	6.87E-04
Pden_5043		UDP-galactopyranose mutase	1.53	0.002290154	0.007366284
Pden_5045		(S)-2-hydroxy-acid oxidase	1.10	0.001182419	0.004676252
Pden_5048		ABC transporter related protein	-1.84	0.00133552	0.005087838
Pden_5049		binding-protein-dependent transport systems inner membrane component	-1.31	0.001923337	0.006575189
Pden_5055		ABC-2 type transporter	2.08	4.42E-04	0.00255247
Pden_5056		ABC transporter related protein	2.71	1.12E-04	0.001190496
Pden_5058		transcriptional regulator, TetR family	2.32	5.34E-04	0.002868014
Pden_5059		2-keto-3-deoxygluconate kinase	2.69	5.04E-05	9.00E-04
Pden_5060		Mannitol dehydrogenase, C-terminal domain	2.50	6.39E-04	0.003190877
Pden_5061	*uxuA*	D-mannonate dehydratase	1.75	1.73E-04	0.001509512
Pden_5083		D-isomer specific 2-hydroxyacid dehydrogenase, NAD-binding protein	1.37	0.003328027	0.009722909
Pden_5084		2-keto-3-deoxygluconate kinase	2.55	1.38E-04	0.001357699
Pden_5085		hydratase/decarboxylase family protein	3.54	2.90E-05	7.04E-04
Pden_5095		amino acid/amide ABC transporter substrate-binding protein, HAAT family	-1.96	0.002861847	0.00868677
Pden_5101		conserved hypothetical protein	1.46	3.72E-04	0.002309828
Pden_5110		putative signal-transduction protein with CBS domains	1.71	1.71E-04	0.001509512
Pden_5115		conserved hypothetical protein	4.02	1.51E-05	6.87E-04
Pden_5117		protein of unknown function DUF1234	3.37	3.34E-04	0.002224238

Pden_5118		conserved hypothetical protein	3.03	1.61E-04	0.001491729
Pden_5119		NADPH-dependent FMN reductase	3.04	1.31E-05	6.87E-04
Pden_5120		hypothetical protein	3.00	2.80E-05	7.03E-04
Pden_5121		hypothetical protein	2.87	4.42E-05	8.44E-04
Pden_5124		hypothetical protein	2.38	0.002704783	0.008311152
Pden_5125		putative monooxygenase protein	2.72	4.00E-05	8.15E-04
Pden_5126		binding-protein-dependent transport systems inner membrane component	2.84	6.03E-04	0.003082284
Pden_5127		transcriptional regulator, Fis family	2.95	2.78E-04	0.001988805
Pden_5128		ABC transporter related protein	3.08	7.55E-04	0.0035394
Pden_5129		RNA polymerase, sigma-24 subunit, ECF subfamily	1.25	0.002426195	0.007691814
Pden_5131		TonB-dependent siderophore receptor	2.42	5.90E-05	9.13E-04
Pden_5132		efflux transporter, RND family, MFP subunit	4.58	4.06E-05	8.15E-04
Pden_5133	*macB3*	ABC transporter related protein	4.75	1.08E-04	0.001177715

Table S5. Protein identification by MALDI-TOF MS with MASCOT search.

Band	Gene	Protein identification	Mass (Da)	E-value	Localization
A	Pden_0569	succinate dehydrogenase flavoprotein subunit	66,031	1.4e-15	IM
B	Pden_0425	electron transfer flavoprotein-ubiquinone oxidoreductase (ETF-QO)	59,659	4.4e-8	IM
C	Pden_3816	ATP synthase F1 subcomplex alpha subunit	55,062	3.5e-10	IM
C	Pden_3818	ATP synthase F1 subcomplex beta subunit	50,308	3.5e-10	IM
D	Pden_2763	outer membrane porin	40,551	4.4e-5	OM

Fig. S1

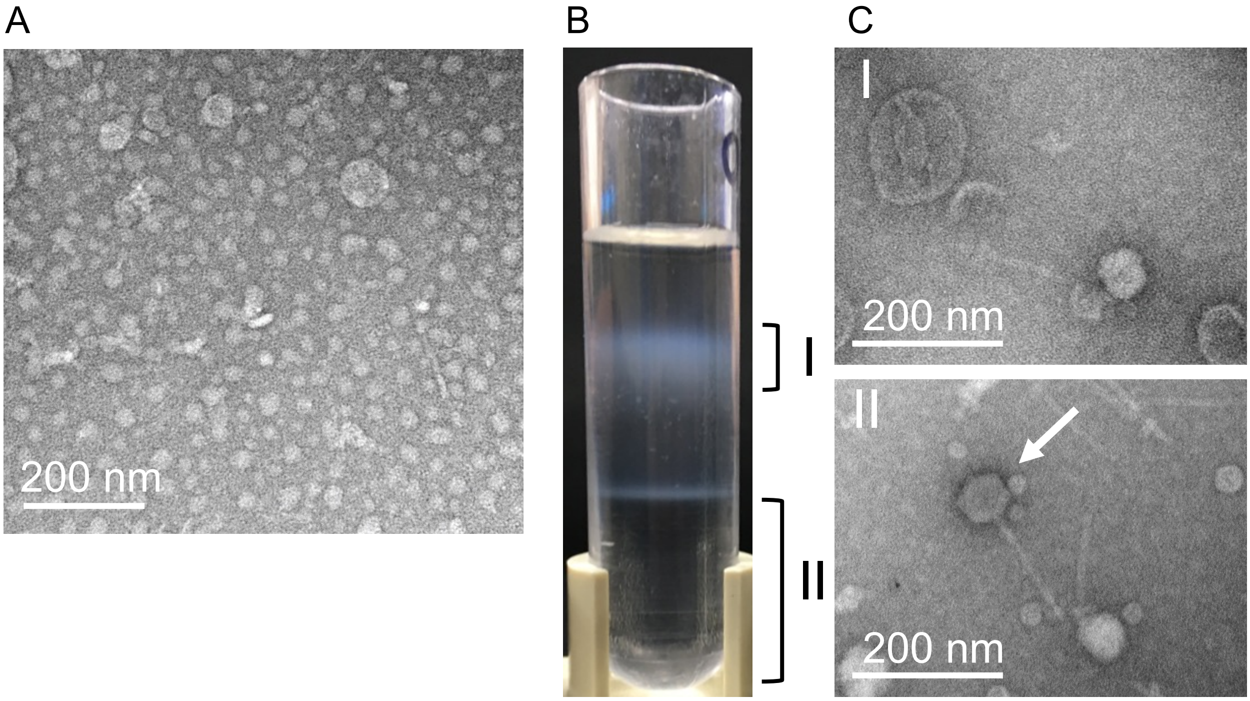

Fig. S1, Yasuda Marina *et al*.

Fig. S2

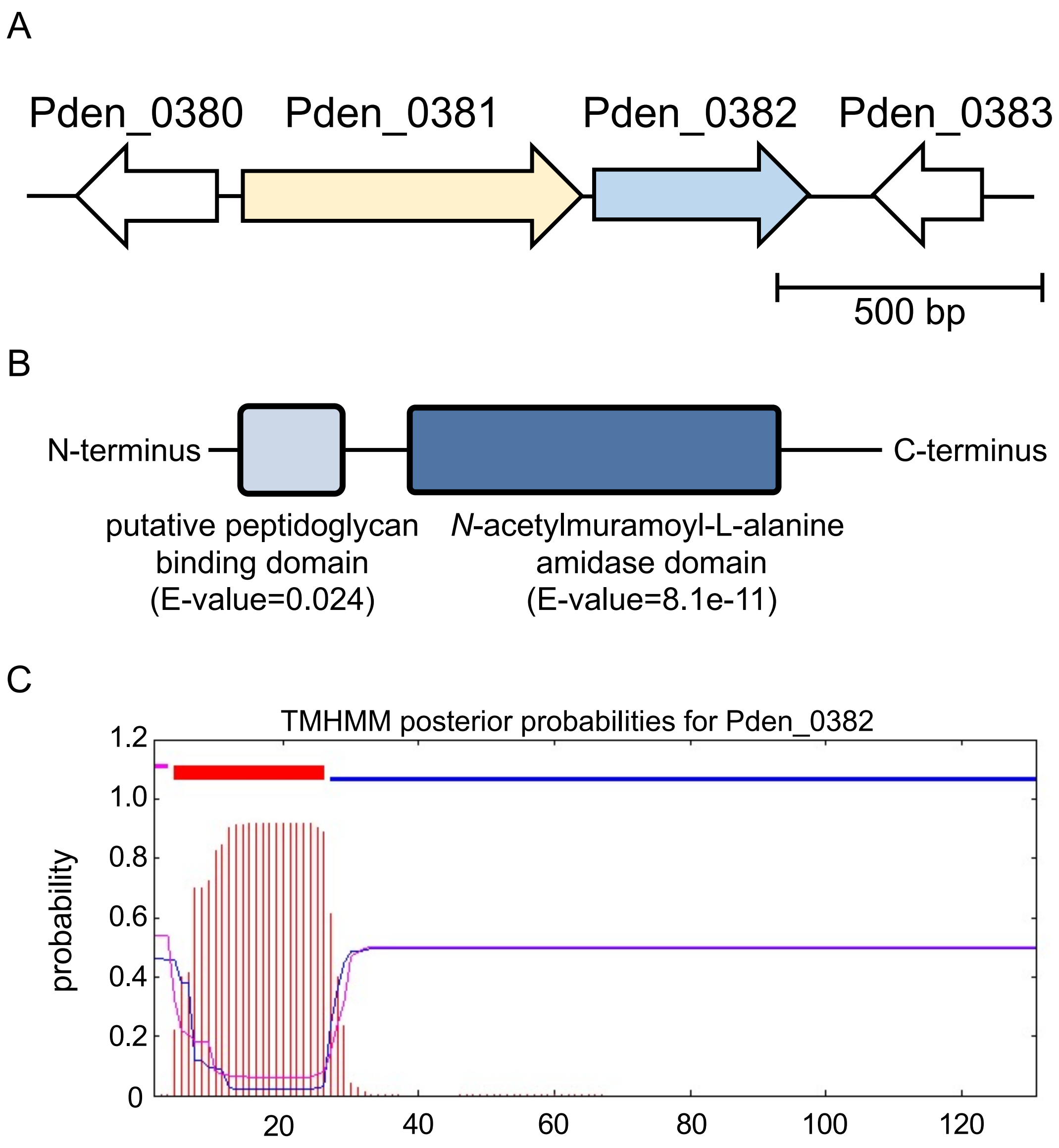

Fig. S2, Yasuda Marina *et al*.

Fig. S3

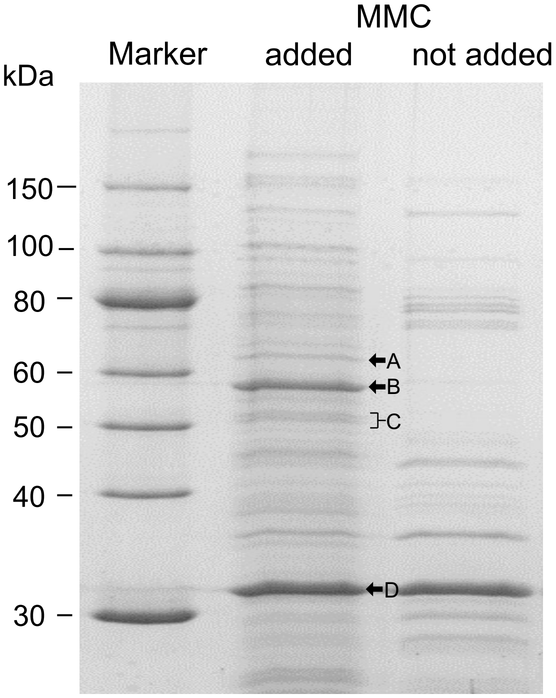

Fig. S3, Yasuda Marina *et al*.

Fig. S4

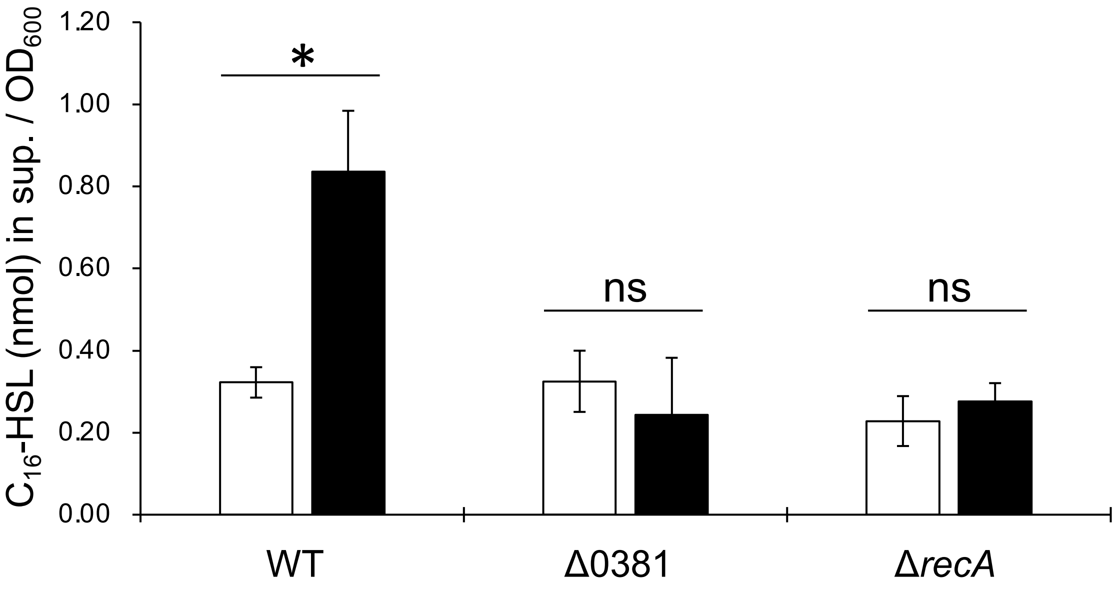

Fig. S4, Yasuda Marina *et al*.

Table S1. List of strains and plasmids used in this study.

Table S2. Sequences of primers used in this study.

Table S3. List of differentially expressed genes between WT with mitomycin C (MMC) *vs.* WT without MMC.

For the differentially expressed genes (logFC>1 or logFC<-1; FDR<0.01) in WT, the following information is given: gene name (based on uniplot or refseq), product, prophage region predicted by *PhiSpy*, log2 fold change (logFC), *p*-value, and false discovery rate (FDR).

Table S4. List of differentially expressed genes between Δ*recA* with MMC *vs.* Δ*recA* without MMC.

For the differentially expressed genes (logFC>1 or logFC<-1; FDR<0.01) in Δ*recA*, the following information is given: gene name (based on uniplot or refseq), product, prophage region predicted by *PhiSpy*, log2 fold change (logFC), *p*-value, and false discovery rate (FDR).

Table S5. Protein identification via MALDI-TOF MS with MASCOT search.

Movie 1. MV release through cell lysis.

Fig. S1. Phage is released with MVs under DNA-damaging stress.

(A) TEM image of the crude MV fraction from MMC-treated WT of *P. denitrificans* Pd1222. (B) TEM image after density gradient centrifugation of the crude MV fraction. The white arrow indicates phage particles.

Fig. S2. Pden_0381 and Pden_0382 are candidates for endolysin and holin in *P. denitrificans* Pd1222.

(A) Gene loci of Pden_0381 and Pden_0382. (B) Motif domain of Pden_0381-coding protein analyzed using KEGG. (C) Transmembrane domain of the Pden_0382-coding protein predicted using the TMHMM Web server.

Fig. S3. Protein profiles of MVs produced with or without MMC.

Approximately 5 μg of protein was separated on a 12% SDS-PAGE gel and stained with Coomassie Brilliant Blue. Band A, succinate dehydrogenase flavoprotein subunit; Band B, electron transfer flavoprotein-ubiquinone oxidoreductase (ETF-QO); Band C, ATP synthase F1 subcomplex alpha subunit and ATP synthase F1 subcomplex beta subunit; Band D, outer membrane porin.

Fig. S4. Pden_0381 allows *P. denitrificans* Pd1222 to release C16-HSL extracellularly in response to MMC.

C16-HSL bioassay using the *C. violaceum* reporter strain in the total supernatant of *P. denitrificans* Pd1222 cultures with or without MMC. C16-HSL concentration in the total supernatant was determined with the aid of *C. violaceum* VIR24/pPROBE-P*vioA*-gfp, without C16-HSL extraction. n=3; mean±s.d. *: $p<0.05$, ns: $p\geq 0.05$ (Welch's t-test).

References

Devries, G.E., Harms, N., Hoogendijk, J., Stouthamer, A.H. (1989). Isolation and characterization of *Paracoccus denitrificans* mutants with increased conjugation frequencies and pleiotropic loss of a (nGATCn) DNA-modifying property. *Arch Microbiol* **152**: 52-57.

Kaczmarczyk, A., Vorholt, J.A., Francez-Charlot, A. (2013). Cumate-inducible gene expression system for sphingomonads and other Alphaproteobacteria. *Appl Environ Microbiol* **79:** 6795-6802.

Schäfer, A., Tauch, A., Jäger, W., Kalinowski, J., Thierbach, G., Pühler, A. (1994). Small mobilizable multi-purpose cloning vectors derived from the *Escherichia coli* plasmids pK18 and pK19: selection of defined deletions in the chromosome of *Corynebacterium glutamicum*. *Gene* **145**: 69-73.

Simon, R., O'Connell, M., Labes, M., Pühler, A. (1986). Plasmid vector for the genetic analysis and manipulation of rhizobia and other gram-negative bacteria. *Methods Enzymol* **118**: 640-659.

Someya, N., Morohoshi, T., Okano, N., Otsu, E., Usuki, K., Sayama, M. *et al.* (2009). Distribution of *N*-Acylhomoserine Lactone-Producing Fluorescent Pseudomonads in the Phyllosphere and Rhizosphere of Potato (Solanum tuberosum L.). *Microbes Environ* **24**:305-314.

Toyofuku, M., Morinaga, K., Hashimoto, Y., Uhl, J., Shimamura, H., Inaba, H. *et al.* (2017). Membrane vesicle-mediated bacterial communication. *ISME J* **11**: 1504-1509.
